# Biological Aging in Combination with Lifestyle Factors in the Blood-Based Methylome: A Biomarker for Colorectal Cancer Susceptibility in African American Women

**DOI:** 10.14336/AD.2025.0099

**Published:** 2025-03-12

**Authors:** Su Yon Jung, Matteo Pellegrini, Herbert Yu

**Affiliations:** ^1^Translational Sciences Section, School of Nursing, University of California, Los Angeles, Los Angeles, CA 90095, USA; ^2^Department of Epidemiology, Fielding School of Public Health, University of California, Los Angeles, Los Angeles, CA 90095, USA; ^3^Jonsson Comprehensive Cancer Center, University of California, Los Angeles, Los Angeles, CA 90095, USA; ^4^Department of Molecular, Cell and Developmental Biology, Life Sciences Division, University of California, Los Angeles, Los Angeles, CA 90095, USA; ^5^Cancer Epidemiology Program, University of Hawaii Cancer Center, Honolulu, HI 96813, USA.

**Keywords:** DNA methylation-based marker for age, epigenetic aging, pre-diagnostic DNA methylation, colorectal cancer, tumorigenesis, exogenous estrogen, diet, African American, postmenopausal women

## Abstract

DNA methylation (DNAm)-based estimators of age are highly accurate for biological aging in multiple tissues, but their functional roles remain poorly understood in colorectal cancer (CRC), an age-related disease whose burden is higher in aged African Americans (AAs) than in whites. A greater rate of epigenetic age deviation from chronologic age was observed in AAs’ colorectal tissues than in whites’, emphasizing AA-specific investigation for epigenetic aging in CRC. Tumor tissue-based DNAm exclusively reflects cancerization, raising a question about its cancer predictability. A prediagnostic peripheral blood leukocyte (PBL)-based DNAm aging marker may thus provide keys to CRC etiology and prevention. From the largest study cohort, we examined 621 AA postmenopausal women 50-79 years old, including a subset of 14 who developed CRC, with their prediagnostic PBL-DNAm. Using three well-known pan-tissue- and blood-based epigenetic clocks, we evaluated correlations with CRC risk and to what degree the cancer risk is modified by lifestyle factors. Epigenetically older age and increased age acceleration were associated with reduced risk for CRC development. Of note, when women had accelerated aging phenotypes at screening, a substantial increased CRC risk was observed in short-term users of exogeneous estrogen, while a profound risk reduction was shown in women eating a healthy diet. Our study contributes to better understanding of the exertion of lifestyle factors in combination with methylome-based aging in colorectal carcinogenesis, detecting a prediagnostic PBL-based aging biomarker that promotes epigenetically targeted strategies tailored to aged AA women at high risk.

## INTRODUCTION

Colorectal cancer (CRC) is a leading cause of cancer incidence and mortality in the U.S.A. and globally [1-3]. CRC is age dependent, with 90% of new cases developing after age 50 [4, 5], highlighting that age is an important risk factor. In particular, African Americans (AAs) experience a greater CRC burden than whites, having an approximately 30% higher incidence rate in those over age 50 than their white counterparts have [6, 7].

Aging is not just referred to as an increase in chronologic age but is also viewed as “biological aging,” which reflects a gradual decline in biological function [8]. Exposures to harmful environments and behavioral factors may affect a variety of biological aging processes at molecular levels, leading to cellular vulnerability, cell senescence, genomic and epigenomic instability, mitochondrial dysfunction, and telomere attrition [9]. Epigenetic modifications—in particular, DNA methylation (DNAm) alterations—have been shown to be the most accurate readouts of aging, referred to as a reduction in the stability of epigenetic marks and the development of epigenetic drift (either negative or positive deviation from chronologic age) [10, 11]. For example, in a monozygotic twin study [12], DNAm at global and regional levels were more different in older than in younger twin pairs, indicating that a reduction in stability of the epigenome is age related. Of note, age-related DNAm modifications frequently occur in the regions of genes shown to be affected by different race [13], suggesting interplay with biological aging and race. Specifically, in colorectal tissues, a different age drift pattern was shown in AAs than in whites: greater DNAm age acceleration (age accel; DNAm age exceeding chronologic age) in whites than in AAs but a greater rate of epigenetic age drift in AAs [14]. This underscores a need for a race-specific investigation into the epigenetic aging process in colorectal diseases such as CRC.

Indeed, DNAm modification is one of the hallmarks of cancer, including CRC [15-17]. At the molecular level, CRC arises largely owing to the lifetime accumulation of genetic and epigenetic alterations in the colon epithelial cells; abnormal DNAm changes often contribute to initiation of irregular stem cell growth in the intestine, mediating field cancerization, and later induce progression of carcinoma [17, 18].

Although chronologic age generally reflects the overall effect of the aging process, the risk of CRC varies substantially between individuals of the same age, indicating heterogeneity in biological aging between people [19]. Thus, there is a need for the development of accurate markers of biological age to catch individuals’ susceptibility to age-related diseases such as CRC. DNAm-based estimators of epigenetic age (“epigenetic clocks”) have been developed [20-22] and are known to capture well the effects of genetic and environmental factors and their interplay across time on cellular function; thus they are highly accurate markers of biological aging, strongly correlating with chronologic age in multiple tissues [20, 23-25]. However, their functional roles in relation to diseases remain poorly understood, particularly in relation to CRC. Related study findings have been inconsistent: mostly positive [26-30], but also negative [19] and null [31] associations between DNAm-based age markers and CRC outcomes. These reported differences can be largely attributable to study designs capturing CRC outcomes (cross-sectional/retrospective vs. prospective); the use of various clocks; blood- or tissue-based DNAm; heterogeneity of age, sex, and race combination in the sample; and population-specific environmental and behavioral profiles.

Of note, cancers do not develop as an isolated phenomenon in their target tissues. Other organ systems are also involved in tumorigenesis through the immune and metabolic systems via the peripheral bloodstream [32]. Also, altered DNAm in peripheral blood leukocytes (PBLs) may reflect genomic interplay with environmental and lifestyle exposures throughout life, causing a disruption of epigenetic balance that increases cancer susceptibility [33]. Meanwhile, tumor tissue-based DNAm may be affected by cancerization in tumor cells, exclusively reflecting the state of differentiation in malignant tissues and expansion of a stem cell pool, raising a question about its cancer predictability. Thus, DNAm age markers, measured in prediagnostic PBLs, the tissue most easily accessible from healthy people, have an important implication in CRC etiology and cancer prediction and prevention.

Because of substantial cancer health disparities in epigenomic science for the AA minorities, it is unclear whether the existing biological aging mechanisms detected from previous studies with a majority of whites are applicable to AAs. In this study, therefore, we focused on AA postmenopausal women, a population highly vulnerable to CRC. We prospectively investigated CRC development and examined a comprehensive set of conventional CRC risk factors in correlation with well-known epigenetic clocks measured via prediagnostic PBL-DNAm. We further examined to what degree CRC risk is modified by selected lifestyle factors. We conducted additional studies with two independent CRC tissue-based DNAm datasets by comparing epigenetic clocks between CRC tissues and normal colon tissues adjacent or those obtained from participants who stayed cancer free. Our study goal was to detect a prediagnostic PBL-based epigenetic aging marker that accounts for lifestyle factors, thus better predicting CRC development; this may contribute to identifying an early-risk group who can benefit from epigenetically informed preventive interventions.

## MATERIALS AND METHODS

### Study population

For the PBL-based genome-wide DNAm data, we obtained from the Women’s Health Initiative Database for Genotypes and Phenotypes (WHI-dbGaP) genetic repository, the largest prospective cohort study of postmenopausal women, ages 50-79 years at enrollment between 1993 and 1998 at > 40 U.S. clinical centers [34, 35]. Within the WHI-dbGaP, we used data from the BAA23, the largest ancillary study (AS) with available PBL-based global-level DNAm by repurposing for our study [36]. DNAm age patterns differ by races [37] and substantial cancer health disparities in epigenomic science exist among minority populations. Thus, our study focused on AA women, a major minority subpopulation in this AS. Among a total of 2,107, 676 AAs were initially included. We excluded women who had been diagnosed with any cancers at enrollment and/or were followed for < 1 year (to minimize reverse causality inference), finally including 621 women, of whom 14 developed primary colorectal carcinoma during a mean 17-year follow-up.

For analyses using CRC tissues, we used two independent datasets with available global-level DNAm from The Cancer Genomic Atlas (TCGA) COADREAD Study [38] and the National Center for Biotechnology Information Gene Expression Omnibus (GEO) database (accession number: GSE199057 [39]). For our study purpose, we included only AAs from each dataset, resulting in totals of 70 tissues (66 CRC and 4 normal adjacent colorectal tissues) from TCGA and 124 tissues (41 CRC, 45 normal adjacent, and 38 normal tissues from participants without CRC) from the GSE199057. Each dataset includes a subset of females (36 CRC and 2 normal adjacent tissues from TCGA; 19 CRC, 19 normal adjacent, and 16 normal tissues from participants without CRC from the GSE199057). The institutional review boards of each WHI clinical center and the University of California, Los Angeles, approved this study.

### Data collection and CRC outcomes

WHI participants had completed self-administered questionnaires at enrollment to provide demographic information (e.g., age, race, ethnicity), comorbid conditions (e.g., ever having been treated for type 2 diabetes [T2DM]), lifestyle factors (e.g., daily diet, including whole fruits, vegetables, and fatty acids intake measured by the Healthy Eating Index [HEI]-2015 [40]; dietary alcohol consumption; years as a regular smoker; and physical activity), and reproductive histories (e.g., a history of ovary removal; use and durations of two types of exogenous estrogen [E] use, including unopposed E-only and opposed E plus progestin [P] from pills or patches). Trained staff obtained their anthropometric measurements (height, weight, and waist and hip circumferences) at screening.

Primary CRC development in the WHI participants was adjudicated by a committee of physicians with a review of the patients’ medical records and pathology/cytology reports and coded into the WHI database according to the National Cancer Institute’s Surveillance, Epidemiology, and End-Results guidelines [41]. The time from enrollment until CRC development, censoring, or study end point was measured as the number of days and converted into years. CRC tissue-based data from TCGA and GSE199057 include information on age, sex, race, and diagnosed tumor type. For our study, we analyzed data from primary colorectal adenocarcinoma tissues, normal adjacent to CRC tissues, and (GEO data only) normal tissues from those who remained cancer free.

### Genome-wide DNAm array and epigenetic clock of aging

Global-level DNAm array of the WHI participants was conducted using their extracted PBL-based DNA via Illumina 450 BeadChip, further beta-mixture quantile (BMIQ)-normalized [42], and batched-adjusted with random intercept for plate and chip and a fixed effect for row [43], resulting in 482,421 CpG dinucleotides (CpGs). To address DNAm stability measured from stored samples [44] in accordance with Horvath’s suggestion [20], we controlled leukocyte heterogeneities in generating DNAm age by employing Houseman’s method [45] for CD4^+^ T cells, natural killer cells, monocytes, and granulocytes, and Horvath’s method [20] for plasma blasts, CD8^+^CD28^-^CD45RA^-^ T cells, and naïve CD8 T cells.

In both TCGA and GSE199057 cohorts, tissue-derived genome-wide DNAm was analyzed by the Illumina Infinium450K and Illumina EPIC, respectively, and using *minfi*, was further normalized via normal-exponential out-of-band (Noob) background correction [46]. Batch effects were corrected using Bland Altman methods for replicate samples [39].

The biological aging clock was measured using an individual’s DNAm level. We employed three well-established epigenetic clocks, including Horvath’s clock [20, 47], a pan-tissue predictor with 353 selected CpGs, Hannum’s PBL-based age with 71 CpGs [21], and Levine’s whole blood and phenotype-based age with 513 CpGs [22]. DNAm age is a composite scale from a linear combination of the weighted CpGs at the individual level. Each clock was generated by an available online tool [20, 47] and the *methylclock* annotation Bioconductor package.

### Statistical analysis

The departure of epigenetic age from chronologic age was calculated by two estimates: 1) AgeAccelDiff, defined by departure of DNAm age from chronologic age, measured by subtracting chronologic age from DNAm age and 2) intrinsic epigenetic age acceleration (IEAA), defined as the residual from regressing DNAm age on chronologic age, which further adjusts for blood cell proportions. The IEAA reflects cell-intrinsic aging effects independent of variations of DNAm levels owing to heterogeneity in cell components between individuals [48].

With each epigenetic clock, we examined the correlation of DNAm age and two epigenetic age-departure estimates (i.e., the AgeAccelDiff and IEAA) with chronologic age by performing linear regression and Spearman’s correlation in all combined and by CRC status. Differences in levels of DNAm age and the two age-departure estimates by selected CRC risk factors were tested via unpaired two-sample *t* or one-way ANOVA tests when applicable. If continuous variables were skewed or had outliers, Mann-Whitney/Wilcoxon’s rank-sum and Kruskal-Wallis tests were used as appropriate. DNAm age and the two age-departure measures were further regressed as continuous and binary outcomes on individual CRC risk factors, referring to a one-unit increase in the risk factor associated with increase in DNAm age/age acceleration (age accel, defined as DNAm age that exceeds chronologic age) in units of years.

In each clock, the distributions of DNAm age and two age-departure estimates by CRC status were examined via unpaired two-sample *t* or Mann-Whitney tests as appropriate. Further, we dichotomized the two age-departure estimates into age accel and age deceleration (age decel, defined as DNAm that falls behind age) and applied the Kaplan-Meier curve and a log rank test. In a multiple Cox proportional hazards regression evaluating the association between DNAm age/age departure and CRC development, we conducted an assumption test via a Schoenfeld residual plot and rho, and adjusted for conventional CRC risk factors [49-52], including age, body mass index (BMI), waist-to-hip ratio (WHR), T2DM, alcohol, smoking, physical activity, and daily fruit, vegetable, and fat intake assessed by HEI-2015, a history of oophorectomy, and hormone replacement therapy. The hazard ratio (HR) from that analysis indicates that a 1-year older DNAm age and age accel increases risk for CRC development. We further examined the association with DNAm age and age accel using a 10-year interval and different segments of the follow-up period. Considering that the testing was derived from our hypothesis-driven questions (i.e., DNAm age is associated with CRC), a two-tailed *p* < 0.05 was considered statistically significant.

With both TCGA and GSE199057 cohorts, we additionally restricted analyses within only females to see whether the results are comparable to those in overall participants. Using only data from the GSE199057, we conducted analyses for epigenetic aging between CRC and normal adjacent CRC tissues and further compared the findings with those from analyses between CRC and normal tissues from participants without CRC.

Lastly, we conducted subset analyses for selected CRC risk factors by stratifying the WHI participants in terms of their epigenetic aging status and examined how DNAm age and age departure estimates modify the effects of risk factors on CRC development.

**Table 1:** Association of DNAmAge in Horvath’s clock with selected CRC risk factors*.

CRC risk factor	Effect size	95% CI	*P*
Age**	1.05	(0.95, 1.14)	8.92E-80
**Years of regular smoking (never vs. < 5 years)**	-1.84	(-4.24, 0.55)	0.131
**5 to < 20 years**	-2.36	(-4.61, -0.12)	0.039
**20 + years**	-1.05	(-3.46, 1.35)	0.390
**Physical activity**	-0.07	(-0.13, -0.01)	0.020
**Physical activity¥ (< 10 MET vs. ≥ 10 MET)**	-1.94	(-3.70, -0.17)	0.031
**Exogenous estrogen only (never use vs. < 5 years)**	-0.09	(-2.34, 2.17)	0.941
**5 to < 10 years**	-3.94	(-7.83, -0.05)	0.047
**10 + years**	0.15	(-2.59, 2.89)	0.913
**Exogenous estrogen plus progestin (never use vs. < 5 years)**	-4.23	(-7.55, -0.92)	0.012
**5 to < 10 years**	0.14	(-5.54, 5.82)	0.961
**10 + years**	-1.81	(-9.02, 5.40)	0.621
**Among only CRC patients**			
**Oophorectomy history (never vs. both ovary removal)**	10.97	(3.31, 18.63)	0.009

CI, confidence interval; CRC, colorectal cancer; DNAmAge, DNA methylation-based marker of aging; MET, metabolic equivalent. Numbers in bold face are statistically significant. * Only factors having a *statistically significant* association with DNAmAge are displayed. ** Age was further significant in a multiple regression model, adjusting for covariates (age, body mass index, waist-to-hip ratio, type 2 diabetes, oophorectomy history, hormone replacement therapy, diet including whole fruits, vegetables, and fatty acids from Healthy Eating Index-2015, alcohol intake, years of regular smoking, and physical activity [except tested variable(s)]). ¥ Physical activity was estimated from recreational physical activity records combining walking and mild, moderate, and strenuous physical activity. Each activity was assigned a MET value corresponding to intensity and the total MET·hours·week per week was stratified into two groups, with 10 METs as the cutoff according to current American College of Sports Medicine and American Health Association recommendations [90].

**Figure 1.**
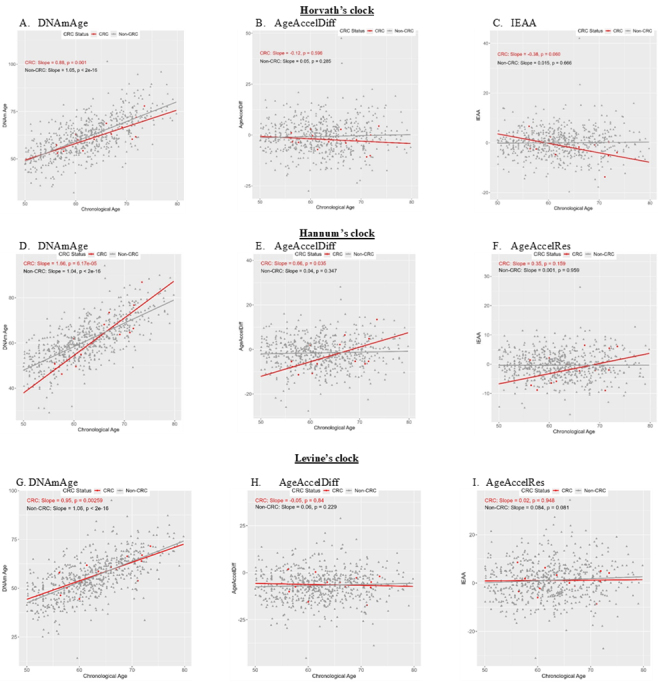
**Correlation between DNAmAge/AgeAccelDiff/IEAA and chronologic age by CRC status**. (AgeAccelDiff, epigenetic age acceleration as departure of DNAmAge from chronologic age; CRC, colorectal cancer; DNAmAge, DNA methylation-based marker of aging; IEAA, intrinsic epigenetic age acceleration as residuals adjusted for cell composition).

## RESULTS

### Correlation between DNAm age/age accel and chronologic age

In all three biologic clocks’ estimates (Fig. 1), DNAm age was positively correlated with chronologic age (age, hereafter) in women who developed CRC as well as in those who did not. For AgeAccelDiff and IEAA, Horvath’s clock revealed a slight negative correlation with age in those with CRC development, but Hannum’s clock showed the opposite, a positive correlation. Levine’s clock showed no substantial correlation with age.

**Figure 2.**
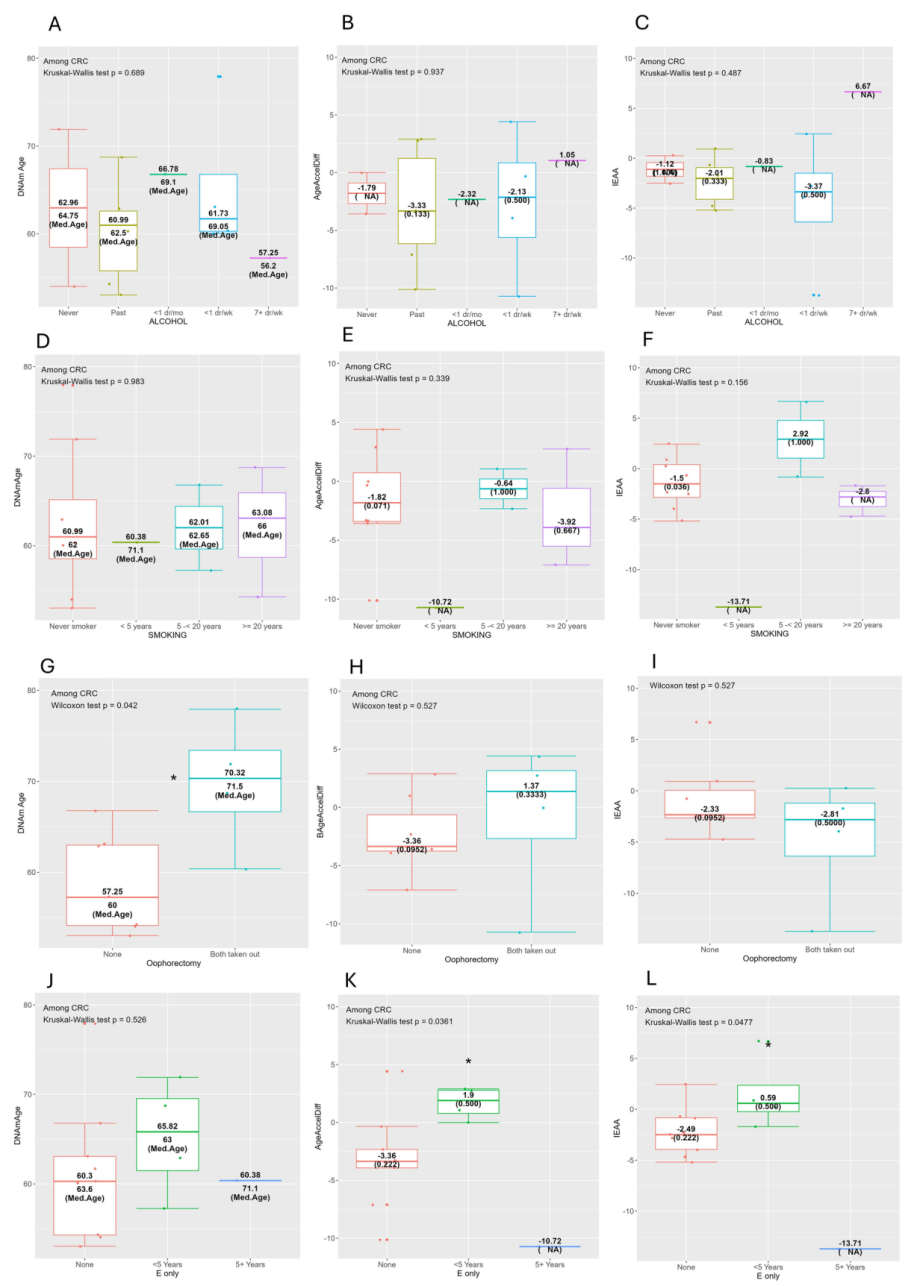
**Horvath’s clock: distribution of DNAmAge/AgeAccelDiff/IEAA by selected CRC risk factors among only CRC patients**. (AgeAccelDiff, epigenetic age acceleration as departure of DNAmAge from chronologic age; CRC, colorectal cancer; DNAmAge, DNA methylation-based marker of aging; E only, exogenous estrogen only; IEAA, intrinsic epigenetic age acceleration as residuals adjusted for cell composition, Med.Age, median chronologic age.) Alcohol: A. DNAmAge, B. AgeAccelDiff, C. IEAA. Years of regular smoking: D. DNAmAge, E. AgeAccelDiff, F. IEAA. Oophorectomy: G. DNAmAge, H. AgeAccelDiff, I. IEAA. E-only: J. DNAmAge, K. AgeAccelDiff, L. IEAA.

**Table 2:** Association of AgeAccelDiff in Horvath’s clock with selected CRC risk factors*.

CRC risk factor	Effect size	95% CI	*P*
**BMI**	0.10	(0.01, 0.19)	0.039
**Physical activity**	-0.05	(-0.10, -0.01)	0.018
**Physical activity¥ (< 10 MET vs. ≥ 10 MET)**	-1.74	(-3.06, -0.42)	0.010
**Exogenous estrogen only (never use vs. < 5 years)**	4.97	(0.31, 9.63)	0.039
**5 + years**	-7.43	(-15.60, 0.75)	0.071
**Among only CRC patients**			
**BMI (normal weight vs. overweight, BMI ≥ 25 to < 30)**	3.78	(-3.06, 10.63)	0.249
**Obesity, BMI ≥ 30 to < 40**	6.71	(0.53, 12.90)	0.036

AgeAccelDiff, epigenetic age acceleration measured as departure of DNAmAge from chronologic age; BMI, body mass index; CI, confidence interval; CRC, colorectal cancer; MET, metabolic equivalent. Numbers in bold face are statistically significant. * Only factors having a *statistically significant* association with AgeAccelDiff are displayed. ¥ Physical activity was estimated from recreational physical activity records combining walking and mild, moderate, and strenuous physical activity. Each activity was assigned a MET value corresponding to intensity and the total MET·hours·week per week was stratified into two groups, with 10 METs as the cutoff according to current American College of Sports Medicine and American Health Association recommendations [90].

### Associations of traditional CRC risk factors with biological aging markers

In Horvath’s clock (Tables 1-3, Supplementary Table 1 and 2; Fig. 2 and Supplementary Fig. 1), several CRC risk factors displayed stronger patterns in women who developed CRC than were apparent in the women overall. Among women with CRC development, those whose alcohol intake was more than 14 g daily had greater age accel in the IEAA than those with moderate and less alcohol intake (≤ 14 g/day) (Table 3). In the breakdown of alcohol use duration, this increased age accel pattern was shown to have a dose-response relationship (Fig. 2B and C): the highest age accel in the AgeAccelDiff and IEAA observed in women with 7+ drinks per week vs. lower age accel observed in those with fewer drinks per week and in never/past drinkers. This pattern was not substantial in overall participants (Supplementary Fig. 1, A-C). Also, among those who developed CRC, relatively short-term regular smokers (< 5 years), compared with never smokers, had decreased age accel in the IEAA but with more years as a regular smoker, age accel was increased, resulting in comparable or greater age-accel level than that of never smokers (Table 3 and Fig. 2D-F). This pattern was not apparent in overall participants (Table 1 and Supplementary Fig. 1D-F).

**Table 3:** Association of IEAA in Horvath’s clock with selected CRC risk factors*.

CRC risk factor	Effect size	95% CI	*P*
**Physical activity**	-0.04	(-0.07, -0.01)	0.019
**Physical activity¥ (< 10 MET vs. ≥ 10 MET)**	-1.23	(-2.18, -0.29)	0.011
**Among only CRC patients**			
**Healthy Eating Index-2015, fatty acids§ (≤ 5.58 vs. > 5.58)**	4.79	(0.08, 9.50)	0.047
**Dietary alcohol intake (g/d)**	0.16	(0.002, 0.32)	0.047
**Dietary alcohol intake (g/d)¶ (≤ 14g vs. > 14g)**	9.34	(0.34, 18.34)	0.043
**Years of regular smoking (never vs. < 5 years)**	-12.33	(-18.98, -5.69)	0.002
**5 to < 20 years**	4.29	(-0.66, 9.25)	0.082
**20 + years**	-1.70	(-5.94, 2.54)	0.394
**Exogenous estrogen only (never use vs. < 5 years)**	3.82	(0.18, 7.46)	0.041
**5 + years**	-11.43	(-17.81, -5.05)	0.002

IEAA, intrinsic epigenetic age acceleration as residuals adjusted for cell composition; CI, confidence interval; CRC, colorectal cancer; MET, metabolic equivalent. Numbers in bold face are statistically significant. * Only factors having a *statistically significant* association with IEAA are displayed. ¥ Physical activity was estimated from recreational physical activity records combining walking and mild, moderate, and strenuous physical activity. Each activity was assigned a MET value corresponding to intensity and the total MET·hours·week per week was stratified into two groups, with 10 METs as the cutoff according to current American College of Sports Medicine and American Health Association recommendations [90]. § Healthy Eating Index-2015, fatty acids, was dichotomized by the median value of 5.58. ¶ Dietary alcohol intake (g/d) was dichotomized by 14g as a moderate drink for women.

Whereas greater intake of whole fruit had a decelerated association with the AgeAccelDiff and IEAA (Supplementary Table 1 and Supplementary Fig. 1G-I) in all participants combined, intake of fatty acids showed complex patterns that differed by CRC status. Among those who remained CRC free, a higher intake of fatty acids (measured as the ratio of poly- and monounsaturated fatty acids [PUFAs and MUFAs] to saturated fatty acids [SFAs]) was associated with younger DNAm age and decreased age accel (Supplementary Table 1 and Supplementary Fig. 1J-L). However, this pattern was the opposite in those who developed CRC: older DNAm age and increased age accel associated with higher fat intake (Table 3 and Supplementary Fig. 1M-O). In addition, women who were physically active, compared with those who were not, had younger DNAm age and decreased age accel measured in the AgeAccelDiff and IEAA; this pattern was consistent across both overall participants and CRC patients (Tables 1-3, Supplementary Table 1 and 2 and Supplementary Fig. 1P-R).

**Table 4:** Risk for CRC development with exogenous estrogen-only use and Healthy Eating Index-2015 whole fruits intake, stratified by biological age

DNAm Age clock	HR	95% CI	*P*	HR†	95% CI	*P*
**Exogenous estrogen-only use for < 5 years compared with never use**						
**Horvath’s clock**						
**AgeAccelDiff negatives**	0.48	(0.06, 3.80)	0.483	0.46	(0.06, 3.75)	0.471
**AgeAccelDiff positives**	17.20	(1.79, 165.48)	0.014	17.78	(1.81, 174.86)	0.014
**IEAA negatives**	0.50	(0.06, 3.96)	0.508	0.53	(0.07, 4.27)	0.550
**IEAA positives**	15.83	(1.65, 152.32)	0.017	16.48	(1.70, 159.61)	0.016
**Hannum’s clock**						
**AgeAccelDiff negatives**	0.67	(0.08, 5.44)	0.707	0.70	(0.08, 5.71)	0.735
**AgeAccelDiff positives**	6.33	(1.06, 37.88)	0.043	6.10	(0.98, 37.92)	0.053
**IEAA negatives**	0.60	(0.07, 4.84)	0.628	0.65	(0.08, 5.35)	0.693
**IEAA positives**	7.26	(1.21, 43.49)	0.030	7.47	(1.18, 47.16)	0.032
**Healthy Eating Index-2015 whole fruit intake > mean 3.73 compare with ≤ mean 3.73**						
**Levine’s clock**						
**DNAmAge ≤ median 55.34 years**	2.72	(0.30, 24.35)	0.371	2.50	(0.27, 23.05)	0.420
**DNAmAge > median 55.34 years**	0.28	(0.07, 1.14)	0.075	0.24	(0.06, 0.97)	0.046

AgeAccelDiff, epigenetic age acceleration measured as departure of DNAmAge from chronologic age; CI, confidence interval; CRC, colorectal cancer; DNAmAge, DNA methylation-based marker of aging; IEAA, intrinsic epigenetic age acceleration as residuals adjusted for cell composition; HR, hazard ratio. Numbers in bold face are statistically significant. † Analyses were further adjusted for obesity variables (body mass index and waist-to-hip ratio).

In terms of reproductive history, women with both ovary removal had older DNAm age and increased age accel in both the AgeAccelDiff and IEAA than women with intact ovaries only in women who developed CRC (Table 1 and Fig. 2G-I); this pattern was not distinct in the women overall (Supplementary Fig. 1S-U). Of note, both unopposed and opposed E-users showed a non-linear association with epigenetic aging but displaying contrary patterns. For instance, among unopposed E-only users who developed CRC, relatively short-term (< 5 years) users had older DNAm age and increased age accel in the AgeAccelDiff and IEAA, compared with never users, but longer-term (5+ years) users had younger DNAm age and decreased age accel (Λ shape) (Tables 3 and Supplementary Table 1 and Fig. 2J-L). However, this pattern was not evident in women who remained CRC free (Tables 1 and 2 and Figures S1, V-X). Opposed E plus P users had a different pattern (V shape), in which the short-term (< 5 years) users had significantly younger DNAm age, and a slight increase in DNAm age/AgeAccelDiff/IEAA was observed in longer-term (5+ years) users (Table 1 and Supplementary Fig. 1Y-AA).

The Hannum’s clock measures (Supplementary Table 3 and Supplementary Fig. 2) showed patterns similar to those from Horvath’s clock, particularly for alcohol use, fatty acid intake, and physical activity. In addition, a higher intake of vegetables yielded age decel in the IEAA in women overall. The same patterns as those in Horvath’s clock were also shown for the reproductive history variables, in that woman who had undergone bilateral oophorectomy and further developed CRC had increased age accel. In Hannum’s clock, all women combined had the Λ-shaped pattern for E-only users in relation to biological aging, showing longer-term users (5+ years) with declined DNAm age, AgeAccelDiff, and IEAA and the V-shaped pattern for E plus P users, displaying short-term users (< 5 years) with younger DNAm age. However, the pattern for smokers differed from that found with Horvath’s clock. In the Hannum’s DNAm age and IEAA measures, the number of years of regular smoking had a negative dose-response relationship: the longer the years as a regular smoker, the steeper the age decel that was observed (Supplementary Fig. 2E-F).

**Figure 3.**
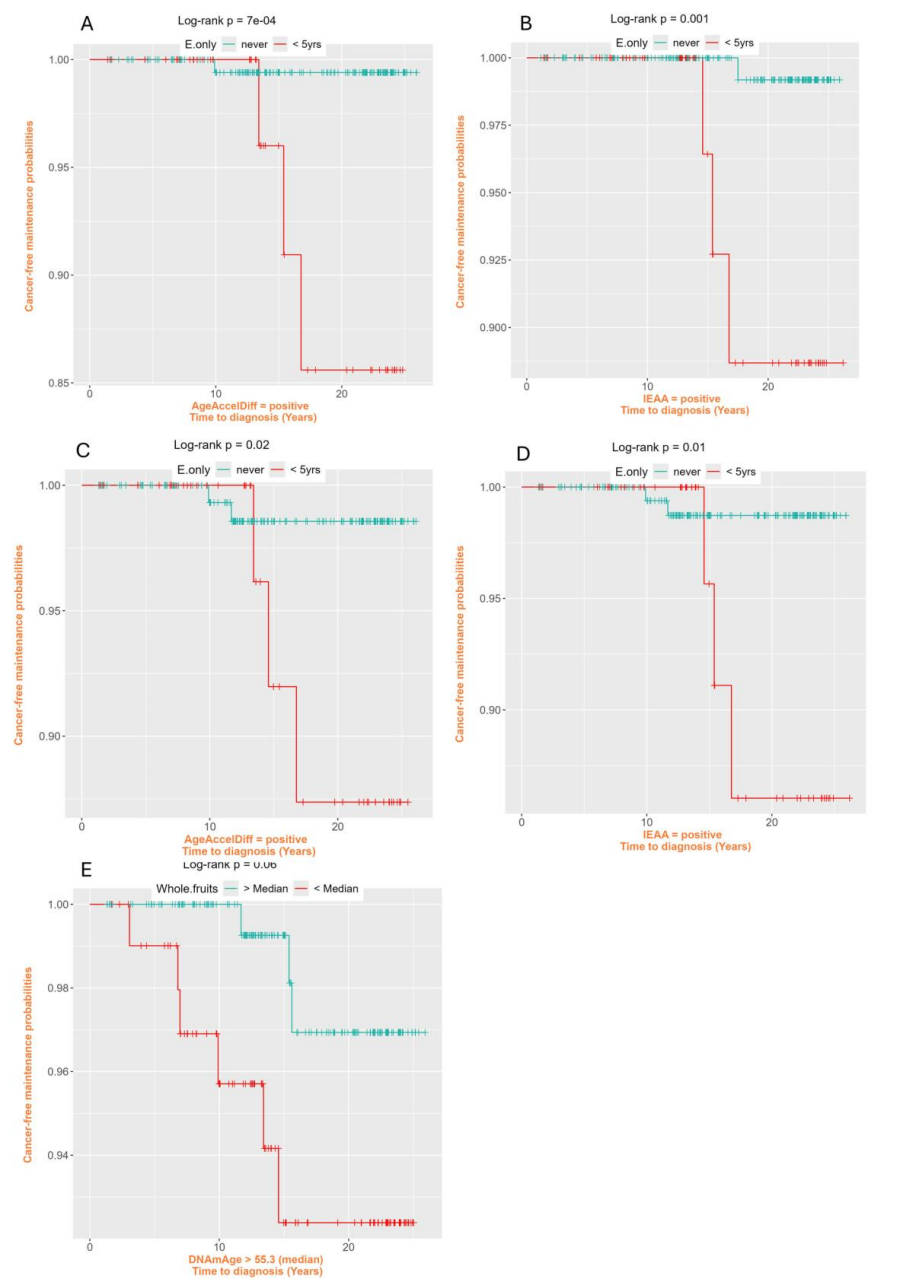
**Cancer-free probability curves of exogenous E-only use and Healthy Eating Index-2015 whole fruits intake among women with older biological ages**. (AgeAccelDiff, epigenetic age acceleration as departure of DNAmAge from chronologic age; DNAmAge; DNA methylation-based marker of aging; E-only, endogenous estrogen only; IEAA, intrinsic epigenetic age acceleration as residuals adjusted for cell composition.) Exogenous E-only use for < 5 years compared with never use: A. Horvath’s clock: Among AgeAccelDiff positives, B. Horvath’s clock: Among IEAA positives, C. Hannum’s clock: AmongAgeAccelDiff positives, D. Hannum’s clock: Among IEAA positives. Healthy Eating Index-2015 whole fruit intake, > mean 3.73 compare with ≤ mean 3.73: E. Levine’s clock: among DNAmAge > median 55.34 years.

Lastly, the analysis of Levine’s clock demonstrated patterns more closely matched to those of Horvath’s clock (Supplementary Table 4 and Supplementary Fig. 3) across all CRC risk factors. Of note, an association between higher WHR and T2DM and significantly increased age accel in the IEAA were found only with Levine’s clock.

### DNAm age and epigenetic age-departure with prospective development of CRC

All three clocks’ patterns were consistent during a 15-year follow-up. A 1-year older DNAm age and age accel was associated with approximately 10% less risk for CRC development (Supplementary Table 5), and this magnitude was profound in Hannum’s clock when evaluated as a 10-year interval: a 10-year increase in age accel was associated with 70% less risk for developing CRC. Similarly, when the AgeAccelDiff and IEAA were categorized into accelerated age (ACC, positive deviation of DNAm age from age) and decelerated age (DCC, negative deviation of DNAm age from age), women with ACC had longer cancer-free intervals than did those with DCC (Supplementary Fig. 4).

### Biological aging in correlation with CRC tissues using TCGA and GSE199057

In TCGA females (Supplementary Tables 6-8 and Supplementary Fig. 5-7), Horvath’s and Levine’s clocks were positively associated with CRC tissues, compared with adjacent normal tissues, whereas the Hannum’s clock showed a negative association with CRC, a similar pattern to that observed in the WHI. These patterns in females, despite lacking statistical significance, were similar to those in both sexes combined. In GSE199057 on females only (Supplementary Tables 9-11 and Supplementary Fig. 8-10), similar to the findings from TCGA females, Levine’s clock was positively associated with CRC tissues, compared with adjacent normal tissues, while both Horvath’s and Hannum’s clocks were negatively correlated with CRC tissues, compared with adjacent normal tissues and normal tissues from women who remained cancer free, respectively.

### Subset analyses by biological aging status for selected risk factors predicting CRC

Given that short-term (< 5 years) E-only users had profoundly older DNAm age and age accel, we next investigated, among older biological ages, how this short-term use further influenced CRC development. As demonstrated with both Horvath’s and Hannum’s age accel measures in the AgeAccelDiff and IEAA, E-only short-term users with positive age accel at baseline had 7-15 times higher risk for developing CRC during the follow-up period (Table 4 and Fig. 3A-3D); these results were consistent after adjustment for obesity. Also, considering the finding that high intake of whole fruits lowered DNAm age, we examined the effect of the high fruit intake on CRC development by biological aging. In Levine’s DNAm age estimate, among older DNAm ages (older than a median of 55.3 years), women with higher intake of whole fruits had about 80% less risk for CRC development (Table 4 and Fig. 3E).

## DISCUSSION

Racial disparities in cancer health are mirrored in genomic science. An inadequate number of omics studies have been conducted among AAs, especially the aged [14], resulting in the severely limited scope of subsequent findings about cancer, an age-related disease. To address this, our study examined AA postmenopausal women, investigating their biological aging with prospective development of CRC, one major cancer burden. Aging involves an accumulation of aberrant DNAm, deregulating the genome and disrupting cell homeostasis [53], which is frequently found in aging tissues, including the colon [54, 55]; it constitutes both driver and passenger events of tumorigenesis. Of note, DNAm alterations reflect the contribution of both physiological and environmental factors. Although the molecular pathways connecting biological aging and CRC is unclear, some studies characterized the underlying mechanisms. For example, CRC with a specific CpG island methylator phenotype (CIMP) represents a high-grade molecular and clinicopathologic feature, hypermethylation of the genes involved in controlling cell growth [56]. This CIMP is more often found in older patients, indicating age-dependent methylation changes in CRC risk and progression [57]. Also, an *in vivo* study [58] reported that the hypermethylation of the promoter in genes of the *Wnt* pathway arose in mouse colon-derived organoids, mimicking the human aging phenotype. The silencing of these genes, in turn, results in a stem-like state and differentiation defects in colonic mucosa. Given the existing variation in CRC prediction even after accounting for age and conventional CRC risk factors, understating the interplay between physiological and systemic dysregulation in epigenetic aging and lifestyle exposures could provide an important clue preceding CRC development and yield accurate risk prediction at the individual level.

We found that all three clocks in PBLs were correlated with age. Considering that Hannum’s clock estimate was derived from the peripheral blood [21], its strongest association with age in our PBL-based DNAm data is not surprising. Also, PBL-based DNAm age and age accel were associated with reduced risk for CRC development with all three clocks; this was largely replicated in our analyses with CRC tissue-level datasets (especially with the GEO cohort), where a 1-year older DNAm age/age accel was significantly associated with about a 10% decrease in CRC. This tissue-based result is consistent with those of previous studies [19, 48, 59]. The decelerated aging in CRC tissues may indicate disruption of the epigenome, rather than only acceleration of the genomic system, by reflecting expansion of the stem cell pool in the tissue which further contributes to cancer aggravation [19, 60]. Of note, limited CRC studies have been performed with PBL-based epigenetic aging, yielding inconsistent reports: two studies [28, 60] with age acceleration in CRC development, in contrast with our results, and one study [26] with age deceleration in those who developed CRC. Also, one recent study [27] using blood samples found no significant association between DNAm age and CRC risk and death. Given that our study results were apparent even after adjusting comprehensive CRC lifestyle factors within 15 years, no significant confounding effect of lifestyles was caught in the evolution of the CRC methylome, but potential reverse causation cannot be completely ruled out, and further validation study is warranted.

According to the multifactorial nature of CRC, several conventional risk factors influence CRC development, including alcohol intake, cigarette smoking, certain dietary patterns, exercise, and obesity-related diseases [51, 61-63]. A growing body of methylome and epigenetic aging research on these lifestyles generally reflects multiple recommendations of the current Dietary Guidelines [64], showing that adhering to healthy lifestyles lowers the rate of biological aging. This can be postulated by their effects on multilevel aging processes, including metabolic regulation and immune response or inflammation [65]. For example, alcohol intake and smoking are associated with aging accel in blood [66, 67], which is in line with our findings, particularly with Horvath’s and Levine’s clocks. Whereas moderate intake of alcohol (≤ 14 g daily or 3-7 drinks/week for women [68]) is reported to be a protective factor against cardiovascular disease (CVD) [69], our finding among women who developed CRC showed increased age accel in those who consumed moderate amounts of alcohol; further, a dose-response relationship was observed: more frequent drinkers (7+ drinks/week) had the highest age accel rate. This indicates that even moderate drinking alcohol has an adverse effect on the development of CRC.

Also, high in fat and low in fiber, vegetables, and fruits, reflecting a Western-style diet, were associated with increased methylation in *p*16, a tumor suppressor, in an *in vivo* study [70], and with shorter leukocyte telomere length [71] and acceleration of aging [72]. Of particular interest, PUFAs, such as omega-3 FAs, have been associated with deceleration of aging [72], which may be through their anti-inflammatory or metabolic effects. Our findings in the women overall agree with those of that study, but among those of our women who developed CRC, the high intake of PUFAs plus MUFAs relative to SFAs has an increased effect on epigenetic aging, although it did not modify the effect of aging on CRC. This warrants future replication and functional studies among a larger CRC cohort with paired blood and tissue samples obtained before and after cancer development.

Our findings that higher intake of fruits and vegetables is associated with lower epigenetic aging are supported by those of a previous study [72] that reported a high blood level of carotenoid, a quantitative surrogate indicator of greater intake of fruit and vegetable, associated with aging decel. This supports their anti-aging effect through the immune system, partially mediated by anti-inflammatory and cardiometabolic exertions, resulting in protection against aging-related diseases, including CVD [73], stroke [74], and T2DM [75]. Whereas our analyses did not show any significant confounding effect of these dietary factors on the methylome-based aging in CRC, we observed a strong modification impact of fruit intake with greater than 80% risk reduction in CRC development among those who consumed more whole fruits, although their baseline epigenetic age was older than the average; but no apparent risk reduction was found in the group with younger epigenetic aging. This finding could suggest that whole fruit intake above a certain threshold is a critical protecting factor for people who have accelerated aging phenotypes; thus, if validated, the methylome-based age could be a useful marker for targeting a dietary intervention against CRC risk.

Exogenous E use among postmenopausal women, in both unopposed and opposed forms, has been found to be protective against CRC development in many observational [76], case-control [77-79], and clinical trial studies [80, 81]; this is also true across different CRC molecular subtypes [82]. These studies generally reported the greatest anti-tumor effect during long-term (5 + or 10 + years) use of unopposed E-only and short-term (< 5 years) use of opposed E plus P. For short-term users, there was a slight, though not statistically significant, increase of CRC risk among E-only users in the WHI trial [80]. These reports robustly support our findings that short-term E-only use was correlated with accelerated aging, but that after 5 years and longer use, age decel was observed. Users of E plus P showed the opposite effect, displaying that short-term use decelerated aging, which is also consistent with the aforementioned previous findings. Exogenous E and P reduce bile acid production [83] and serum levels of insulin, glucose, and insulin-like growth factors [84, 85], all of which have been postulated to enhance colorectal carcinogenesis. This hormonal therapy also modulates estrogen receptors’ intracellular signaling cascades in the intestine epithelium [86], where these nuclear receptors exert an anti-tumor effect on the colonic epithelium by selectively activating pro-apoptotic signaling, increasing DNA repair, and inhibiting oncogene expression [87].

Among our short-term E-only users, the CRC-promoting effect was not observed in the decelerated aging group, but a profound increased risk of CRC was shown when their baseline epigenetic aging was accelerated. This reflects a deviation from the previously accepted mechanism of exogenous E’s exertion on CRC risk, particularly when physiological and systemic aging processes are involved. It is noteworthy that another estrogen binding receptor, the G protein-coupled estrogen receptor, plays an essential role in colorectal carcinogenesis by elevating the expression and activity of several oncogenes such as *FASN* in the colon through *EGFR/ERK/c-Fos/AP-1* signaling, which subsequently results in increased CRC cell growth, invasion, and migration [87, 88]. These tumorigenic pathways are postulated to be exacerbated during the early period of E use, specifically in those who have accelerated aging phenotypes. Overall, our findings have an important clinical implication concerning the role of epigenetic aging markers in early identification of a group at risk with tailored intensive screening as a CRC preventive strategy.

One of our tissue-based cohorts had undergone an EPIC array, differing from the other cohorts, which used an HM450 array. We confirmed that the missing CpGs on the EPIC did not significantly affect the accuracy of the epigenetic age determination [89] and that the sensitivity testing results with common CpGs across the two arrays were nearly identical [19]. Also, our analysis of TCGA and GEO data lacked an extensive set of covariates, leading to a lack of confirmatory findings; this deserves a future independent, large replication study. Our data has limited clinical information available, such as CRC molecular subtypes and location, which reduces our ability to account for several characteristics that are related to the CRC methylome. Also, we cannot completely rule out residual confounding, especially about self-reported lifestyle factors. Our focus on only AA elderly women limits our statistical power and the generalizability of our findings to other populations. Our observational study yielded purely preliminary pilot results owing to the relatively small sample size, and multiple combinations of lifestyle factors in the analyses resulted in several extreme ranges of risk magnitudes, cautioning about false positives and warranting a further replication study with a larger independent dataset. However, our study has the potential to promote the development of an epigenetically guided decision-making process in a race-specific manner and to provide tailored cancer-preventive interventions. Given that the racial disparity in CRC is heavily affected by the location of the cancer [14], a future study for race-specific epigenetic aging by colorectal location could enhance our understanding of the aged methylome and interplay with race in the etiology of CRC. Also, the unique environment of the colon, such as the close interaction with the gut microbiome and digestion products, may result in a colon-specific aging pattern in a tissue-specific way, so developing a colon-specific aging clock by integrating such cumulative microbiome and diet effects is warranted. While epigenetic clocks are promising, more effort is recommended before we consider their application in clinical practice: assessment of their cost-effectiveness, development of standardized research protocols, evaluating their reproducibility across relevant tissue types, and validation in diverse populations.

In summary, epigenetically older age and age accel were associated with reduced risk for CRC development, and among those with accelerated aging phenotypes, a substantially increased risk was observed in short-term users of exogeneous E, whereas a profound risk reduction was shown in those with a healthy diet. Our study contributes to a better understanding of the role of lifestyle factors in combination with methylome-based aging in colorectal carcinogenesis, promoting epigenetically targeted strategies tailored to aged AA women at high risk for CRC.

## Supplementary Materials

The Supplementary data can be found online at: www.aginganddisease.org/EN/10.14336/AD.2025.0099.

## Data Availability

The data that support the findings of this study are available in accordance with policies developed by the NHLBI and WHI in order to protect sensitive participant information and approved by the Fred Hutchinson Cancer Research Center, which currently serves as the IRB of record for the WHI. Data requests may be made by emailing helpdesk@WHI.org.
