# Supplementary material for: Biological Aging in Combination with Lifestyle Factors in the Blood-Based Methylome: A Biomarker for Colorectal Cancer Susceptibility in African American Women
Source: Aging Dis. 2025 Mar 12;17(2):1052–67. doi: 10.14336/AD.2025.0099 (PMC12834397; doi:10.14336/AD.2025.0099)
Supplement: Supplementary file 1 — The Supplementary data can be found online at: www.aginganddisease.org/EN/10.14336/AD.2025.0099. [file AD-17-2-1052-s.pdf]

Supplementary Data

Biological Aging in Combination with Lifestyle Factors in the Blood-Based Methylome: A Biomarker for Colorectal Cancer Susceptibility in African American Women

Su Yon Jung, Matteo Pellegrini, Herbert Yu

© 2025. Jung SY et al. Published online at http://www.aginganddisease.org/EN/10.14336/AD.2025.0099

Supplementary Data

Supplementary Table 1. Association of AgeAccelDiff in Horvath's clock with selected CRC risk factors*; AgeAccelDiff was analyzed as binary outcomes (negatives vs. positives)

CRC risk factor	OR	95% CI	*P*
BMI	**1.03**	**(1.00, 1.05)**	**0.021**
Healthy Eating Index-2015, whole fruits	**0.90**	**(0.81, 0.99)**	**0.037**
Healthy Eating Index-2015, fatty acids**	**0.92**	**(0.86, 0.98)**	**0.010**
Physical activity	**0.98**	**(0.96, 0.99)**	**0.001**
Physical activity¥** (< 10 MET vs. ≥ 10 MET)	**0.53**	**(0.37, 0.76)**	**0.001**
<u>Among only CRC patients</u>			
Exogenous estrogen only (never use vs. < 5 years)	**24.00**	**(1.55, 1035.87)**	**0.043**
5 + years	NA	NA	NA

AgeAccelDiff, epigenetic age acceleration measured as departure of DNAmAge from chronologic age; BMI, body mass index; CI, confidence interval; CRC, colorectal cancer; MET, metabolic equivalent; OR, odds ratio. Numbers in bold face are statistically significant.

* Only factors having a *statistically significant* association with AgeAccelDiff are displayed.

** Variables were further significant in a multiple regression model, adjusting for covariates (age, BMI, waist-to-hip ratio, type 2 diabetes, oophorectomy history, hormone replacement therapy, diet including whole fruits, vegetables, and fatty acids from Healthy Eating Index-2015, alcohol intake, years of regular smoking, and physical activity [except tested variable(s)]).

¥ Physical activity was estimated from recreational physical activity records combining walking and mild, moderate, and strenuous physical activity. Each activity was assigned a MET value corresponding to intensity and the total MET·hours·week per week was stratified into two groups, with 10 METs as the cutoff according to current American College of Sports Medicine and American Health Association recommendations [87].

Supplementary Table 2. Association of IEAA in Horvath's clock with selected CRC risk factors*; IEAA was analyzed as binary outcomes (negatives vs. positives)

CRC risk factor	OR	95% CI	*P*
Physical activity	**0.98**	**(0.97, 1.00)**	**0.007**
Physical activity¥** (< 10 MET vs. ≥ 10 MET)	**0.63**	**(0.44, 0.89)**	**0.008**

CI, confidence interval; CRC, colorectal cancer; IEAA, intrinsic epigenetic age acceleration as residuals adjusted for cell composition; MET, metabolic equivalent; OR odds ratio. Numbers in bold face are statistically significant.

* Only factors having a *statistically significant* association with AgeAccelDiff are displayed.

¥ Physical activity was estimated from recreational physical activity records combining walking and mild, moderate, and strenuous physical activity. Each activity was assigned a MET value corresponding to intensity and the total MET·hours·week per week was stratified into two groups, with 10 METs as the cutoff according to current American College of Sports Medicine and American Health Association recommendations [87].

** Physical activity was further significant in a multiple regression model, adjusting for covariates (age, body mass index, waist-to-hip ratio, type 2 diabetes, oophorectomy history, hormone replacement therapy, diet including whole fruits, vegetables, and fatty acids from Healthy Eating Index-2015, alcohol intake, years of regular smoking, and physical activity [except tested variable(s)]).

© 2025. Jung SY et al. Published online at http://www.aginganddisease.org/EN/10.14336/AD.2025.0099

Supplementary Table 3. Association of DNAmAge/AgeAccelDiff/IEAA in Hannum's clock with selected CRC risk factors*; AgeAccelDiff and IEAA were further analyzed as binary outcomes (negatives vs. positives)

CRC risk factor	Effect size	95% CI	*P*
**A. DNAmAge (Continuous outcomes)**			
Age**	**1.05**	**(0.97, 1.13)**	**4.61E-98**
Healthy Eating Index-2015, fatty acids (≤ 5.58 vs. > 5.58) §¥	**-1.66**	**(-2.85, -0.47)**	**0.006**
Years of regular smoking (never vs. < 5 years)	-0.14	(-2.36, 2.09)	0.903
5 to < 20 years	**-2.20**	**(-4.28, -0.11)**	**0.039**
20 + years	-2.11	(-4.35, 0.12)	0.064
Exogenous estrogen only (never use vs. < 5 years)	0.08	(-2.01, 2.18)	0.940
5 to < 10 years	**-3.88**	**(-7.50, -0.26)**	**0.035**
10 + years	0.24	(-2.31, 2.78)	0.854
Exogenous estrogen plus progestin (never use vs. < 5 years)	**-5.23**	**(-8.30, -2.17)**	**0.001**
5 to < 10 years	-1.66	(-6.92, 3.60)	0.536
10 + years	-1.92	(-8.59, 4.76)	0.573
<u>Among only CRC patients</u>			
Oophorectomy history (never vs. both ovary removal)	**17.01**	**(3.80, 30.22)**	**0.016**
**B. AgeAccelDiff (Continuous outcomes)**			
BMI	**0.09**	**(0.01, 0.17)**	**0.025**
Healthy Eating Index-2015, fatty acids (≤ 5.58 vs. > 5.58) **¥	**-1.21**	**(-2.31, -0.11)**	**0.031**
Oophorectomy history (never vs. both ovary removal)	**1.66**	**(0.31, 3.01)**	**0.016**
Exogenous estrogen only (never use vs. < 5 years)	0.24	(-1.46, 1.95)	0.779
5 + years§	**-1.85**	**(-3.69, -0.01)**	**0.049**
<u>Among only CRC patients</u>			
Age	**0.66**	**(0.06, 1.26)**	**0.035**
**C. AgeAccelDiff (Binary outcomes)**	**OR**	**95% CI**	***p***
Years of regular smoking (never vs. < 5 years)	**1.63**	**(1.02, 2.61)**	**0.040**
5 to < 20 years	1.06	(0.67, 1.65)	0.807
20 + years	1.13	(0.70, 1.81)	0.617
Physical activity¥ (< 10 MET vs. ≥ 10 MET)	**0.68**	**(0.48, 0.97)**	**0.034**
**D. IEAA (Continuous outcomes)**			
Years a regular smoker (never vs. < 5 years)	0.77	(-0.29, 1.83)	0.155

© 2025. Jung SY et al. Published online at http://www.aginganddisease.org/EN/10.14336/AD.2025.0099

5 to < 20 years	-0.87	(-1.87, 0.12)	0.084
20 + years**	**-1.34**	**(-2.41, -0.28)**	**0.013**
Oophorectomy history (never vs. both ovary removal)	**1.02**	**(0.09, 1.96)**	**0.032**
**E. IEAA (Binary outcomes)**	**OR**		
BMI (normal weight vs. overweight, BMI ≥ 25 to < 30)	1.11	(0.59, 2.12)	0.739
Obesity, BMI ≥ 30 to < 40§	**1.85**	**(1.01, 3.45)**	**0.049**
Extreme obesity, BMI ≥ 40	1.31	(0.62, 2.77)	0.481
Healthy Eating Index-2015, vegetables¥ (≤ 3.49 vs. > 3.49)§	**0.66**	**(0.45, 0.97)**	**0.035**
Exogenous estrogen only (never use vs. < 5 years)	0.95	(0.61, 1.48)	0.835
5 to < 10 years	1.25	(0.59, 2.68)	0.561
10 + years	**0.54**	**(0.30, 0.94)**	**0.033**

AgeAccelDiff, epigenetic age acceleration measured as departure of DNAmAge from chronologic age; BMI, body mass index; CI, confidence interval; CRC, colorectal cancer; DNAmAge, DNA methylation–based marker of aging; IEAA, intrinsic epigenetic age acceleration as residuals adjusted for cell composition; MET, metabolic equivalent; OR, odds ratio. Numbers in bold face are statistically significant.

* Only factors having a *statistically significant* association with DNAmAge/AgeAccelDiff/IEAA are displayed.

** Variables were further significant in a multiple regression model, adjusting for covariates (age, BMI, waist-to-hip ratio, type 2 diabetes, oophorectomy history, hormone replacement therapy, diet including whole fruits, vegetables, and fatty acids from Healthy Eating Index-2015, alcohol intake, years of regular smoking, and physical activity [except tested variable(s)]).

§ Variables were significant only in a multiple regression model.

¥ Healthy Eating Index-2015, fatty acids, was dichotomized by the median 5.58; Healthy Eating Index-2015, vegetables, was dichotomized by the median 3.49; Physical activity was estimated from recreational physical activity records combining walking and mild, moderate, and strenuous physical activity. Each activity was assigned a MET value corresponding to intensity and the total MET·hours·week per week was stratified into two groups, with 10 METs as the cutoff according to current American College of Sports Medicine and American Health Association recommendations [87].

Supplementary Table 4. Association of DNAmAge/AgeAccelDiff/IEAA in Levine's clock with selected CRC risk factors*; AgeAccelDiff and IEAA were further analyzed as binary outcomes (negatives vs. positives)

CRC risk factor	Effect size	95% CI	*P*
**A. DNAmAge (Continuous outcomes)**			
Age**	**1.06**	**(0.96, 1.16)**	**3.22E-74**
Healthy Eating Index-2015, fatty acids**	**-0.34**	**(-0.67, -0.01)**	**0.045**
Years of regular smoking (never vs. < 5 years)	-1.34	(-3.82, 1.14)	0.288
5 to < 20 years	**-2.63**	**(-4.95, -0.31)**	**0.026**
20 + years	-0.42	(-2.91, 2.06)	0.738
Alcohol intake (never vs. past drinker)	0.41	(-1.82, 2.64)	0.719
<1 drink per month	-0.74	(-3.53, 2.05)	0.602
<1 drink per week	-0.19	(-2.59, 2.21)	0.875

© 2025. Jung SY et al. Published online at http://www.aginganddisease.org/EN/10.14336/AD.2025.0099

1 to <7 drinks per week	1.01	(-1.88, 3.90)	0.494
7+ drinks per week§	**3.96**	**(0.13, 7.79)**	**0.043**
Physical activity¥ (< 10 MET vs. ≥ 10 MET)	**-1.91**	**(-3.75, -0.07)**	**0.042**
Exogenous estrogen plus progestin (never use vs. < 5 years)	**-3.46**	**(-6.92, 0.002)**	**0.050**
5 to < 10 years	-0.75	(-6.68, 5.18)	0.804
10 + years	1.89	(-5.63, 9.41)	0.622
Among only CRC patients			
Healthy Eating Index-2015, whole fruits¥ (≤ 3.73 vs. > 3.73)	**-8.53**	**(-16.46, -0.61)**	**0.037**
**B. AgeAccelDiff (Continuous outcomes)**			
BMI	**0.10**	**(0.004, 0.20)**	**0.040**
Healthy Eating Index-2015, fatty acids	**-0.26**	**(-0.51, -0.004)**	**0.047**
Healthy Eating Index-2015, fatty acids¥ (≤ 5.58 vs. > 5.58)**	**-1.38**	**(-2.72, -0.04)**	**0.044**
Dietary alcohol intake (g/d)	**0.09**	**(0.02, 0.15)**	**0.007**
Alcohol intake (never vs. past drinker)	0.76	(-1.10, 2.61)	0.423
<1 drink per month	-0.05	(-2.53, 2.43)	0.967
<1 drink per week	-0.71	(-2.77, 1.35)	0.498
1 to <7 drinks per week	0.86	(-1.61, 3.33)	0.493
7+ drinks per week**	**4.84**	**(1.34, 8.34)**	**0.007**
Physical activity¥ (< 10 MET vs. ≥ 10 MET)	**-1.72**	**(-3.13, -0.30)**	**0.018**
Among only CRC patients			
Years of regular smoking (never vs. < 5 years)	**-9.79**	**(-19.17, -0.42)**	**0.042**
5 to < 20 years	**7.01**	**(0.02, 13.99)**	**0.050**
20 + years	3.36	(-2.63, 9.34)	0.240
Exogenous estrogen only (never use vs. < 5 years)	-0.50	(-6.73, 5.73)	0.864
5 + years	**-11.80**	**(-22.73, -0.87)**	**0.037**
**C. AgeAccelDiff (Binary outcomes)**	**OR**	**95% CI**	***p***
Healthy Eating Index-2015, fatty acids	**0.92**	**(0.85, 1.00)**	**0.042**
Dietary alcohol intake (g/d)	**1.04**	**(1.01, 1.07)**	**0.007**
Dietary alcohol intake (g/d)¥ (≤ 14g vs. > 14g)	**4.13**	**(1.62, 10.25)**	**0.002**
Alcohol intake (never vs. past drinker)	1.54	(0.83, 2.95)	0.179
<1 drink per month	1.42	(0.61, 3.20)	0.404
<1 drink per week	1.04	(0.50, 2.17)	0.909
1 to <7 drinks per week	1.25	(0.53, 2.86)	0.597
7+ drinks per week**	**5.46**	**(2.14, 14.07)**	**0.0004**
Physical activity¥ (< 10 MET vs. ≥ 10 MET)	**0.59**	**(0.36, 0.94)**	**0.032**
**D. IEAA (Continuous outcomes)**			
Age§	**0.12**	**(0.01, 0.22)**	**0.031**

© 2025. Jung SY et al. Published online at http://www.aginganddisease.org/EN/10.14336/AD.2025.0099

BMI	**0.09**	**(0.002, 0.19)**	**0.045**
Waist-to-hip ratio¥ (≤ 0.85 vs. > 0.85)	**1.33**	**(0.09, 2.57)**	**0.036**
Type 2 diabetes	**2.10**	**(0.47, 3.72)**	**0.011**
Physical activity¥ (< 10 MET vs. ≥ 10 MET)	**-1.51**	**(-2.83, -0.18)**	**0.026**
Among only CRC patients			
Years of regular smoking (never vs. < 5 years)	**-9.03**	**(-17.93, -0.14)**	**0.047**
5 to < 20 years	**7.66**	**(1.03, 14.29)**	**0.028**
20 + years	1.31	(-4.37, 6.98)	0.619
**E. IEAA (Binary outcomes)**	**OR**	**95% CI**	***p***
Age**	**1.03**	**(1.01, 1.06)**	**0.015**
Waist-to-hip ratio	**9.06**	**(1.35, 69.21)**	**0.029**
Type 2 diabetes	**1.55**	**(1.01, 2.42)**	**0.049**
Physical activity	**0.99**	**(0.97, 1.00)**	**0.024**
Physical activity¥ (< 10 MET vs. ≥ 10 MET)	**0.63**	**(0.45, 0.89)**	**0.009**
Exogenous estrogen only (never use vs. < 5 years)	1.54	(0.98, 2.45)	0.062
5 to < 10 years	0.65	(0.30, 1.37)	0.257
10 + years**	**0.52**	**(0.30, 0.89)**	**0.019**

AgeAccelDiff, epigenetic age acceleration measured as departure of DNAmAge from chronologic age; BMI, body mass index; CI, confidence interval; CRC, colorectal cancer; DNAmAge, DNA methylation– based marker of aging; IEAA, intrinsic epigenetic age acceleration as residuals adjusted for cell composition; MET, metabolic equivalent; OR, odds ratio. Numbers in bold face are statistically significant.

* Only factors having a *statistically significant* association with DNAmAge/AgeAccelDiff/IEAA are displayed.

** Variables were further significant in a multiple regression model, adjusting for covariates (age, BM, waist-to-hip ratio, type 2 diabetes, oophorectomy history, hormone replacement therapy, diet including whole fruits, vegetables, and fatty acids from Healthy Eating Index-2015, alcohol intake, years of regular smoking, and physical activity [except tested variable(s)]).

§ Variables were statistically significant only in a multiple regression model.

¥ Physical activity was estimated from recreational physical activity records combining walking and mild, moderate, and strenuous physical activity. Each activity was assigned a MET value corresponding to intensity and the total MET·hours·week per week was stratified into two groups, with 10 METs as the cutoff according to current American College of Sports Medicine and American Health Association recommendations [87]; Healthy Eating Index-2015, whole fruits, was dichotomized by the mean 3.73; Healthy Eating Index-2015, fatty acids, was dichotomized by the median 5.58; dietary alcohol intake (g/d) was dichotomized by 14g as a moderate drink for women; waist-to-hip ratio was categorized using 0.85 as the cutoff, at which higher values fall into the viscerally obese range (ref. Waist circumference and waist– hip ratio: report of a WHO expert consultation. Geneva: World Health Organization; 2008).

© 2025. Jung SY et al. Published online at http://www.aginganddisease.org/EN/10.14336/AD.2025.0099

Supplementary Table 5. Multiple Cox regression for DNAmAge/AgeAccelDiff/IEAA predicting CRC development within 15 years

DNAm clock	HR†	95% CI	*P*
**Horvath's clock***			
DNAmAge	**0.89**	**(0.82, 0.97)**	**0.011**
AgeAccelDiff	0.97**	(0.89, 1.06)	0.544
IEAA	0.92**	(0.81, 1.04)	0.178
**Hannum's clock***			
DNAmAge	0.95	(0.90, 1.01)	0.114
DNAmAge, 10-year interval	0.57	(0.31, 1.05)	0.073
AgeAccelDiff	**0.88**	**(0.79, 0.98)**	**0.024**
AgeAccelDiff, 10-year interval	**0.36**	**(0.14, 0.92)**	**0.032**
IEAA	**0.83**	**(0.71, 0.99)**	**0.033**
IEAA, 10-year interval	**0.11**	**(0.02, 0.74)**	**0.023**
**Levine's clock***			
DNAmAge	0.96	(0.90, 1.02)	0.174
AgeAccelDiff	**0.87**	**(0.80, 0.95)**	**0.002**
IEAA	**0.81**	**(0.72, 0.91)**	**0.0003**

AgeAccelDiff, epigenetic age acceleration measured as departure of DNAmAge from chronologic age; CI, confidence interval; CRC, colorectal cancer; DNAmAge, DNA methylation–based marker of aging; HR, hazard ratio; IEAA, intrinsic epigenetic age acceleration as residuals adjusted for cell composition. Numbers in bold face are statistically significant.

* DNAmAge/AgeAccelDiff/IEAA were each analyzed as a continuous variable.

† HR adjusted for all covariates (age, body mass index, waist-to-hip ratio, type 2 diabetes, oophorectomy history, hormone replacement therapy, diet including whole fruits, vegetables, and fatty acids from Healthy Eating Index-2015, alcohol intake, years of regular smoking, and physical activity).

** Analyses were adjusted for only obesity variables (body mass index and waist-to-hip ratio).

© 2025. Jung SY et al. Published online at http://www.aginganddisease.org/EN/10.14336/AD.2025.0099

Supplementary Table 6. TCGA Horvath' clock: Validation tests. Logistic regression results for the DNAmAge/AgeAccelDiff AgeAccelRes predicting CRC

< Overall >				< Females >			
**Analysis**	**OR†**	**95% CI**	***P***	**Analysis**	**OR†**	**95% CI**	***P***
**DNAmAge**	0.97	(0.92, 1.04)	0.418	**DNAmAge**	1.03	(0.93, 1.12)	0.586
**AgeAccelDiff**	1.00	(0.95, 1.07)	0.971	**AgeAccelDiff**	1.03	(0.92, 1.17)	0.651
**AgeAccelRes**	0.99	(0.93, 1.06)	0.682	**AgeAccelRes**	1.04	(0.91, 1.19)	0.534

AgeAccelDiff, epigenetic age acceleration measured as departure of DNAmAge from chronologic age; AgeAccelRes, epigenetic age acceleration as residuals by regressing DNAmAge on chronologic age; CI, confidence interval; CRC, colorectal cancer; DNAmAge, DNA methylation–based marker of aging; OR, odds ratio; TCGA, The Cancer Genomic Atlas.
† ORs obtained from univariate analyses.

Supplementary Table 7. TCGA Hannum's clock: Validation tests. Logistic regression results for the DNAmAge/AgeAccelDiff AgeAccelRes predicting CRC

< Overall >				< Females >			
**Analysis**	**OR†**	**95% CI**	***P***	**Analysis**	**OR†**	**95% CI**	***P***
**DNAmAge**	0.98	(0.93, 1.04)	0.414	**DNAmAge**	0.98	(0.92, 1.06)	0.578
**AgeAccelDiff**	1.00	(0.96, 1.06)	0.958	**AgeAccelDiff**	0.97	(0.89, 1.06)	0.447
**AgeAccelRes**	0.95	(0.90, 1.01)	0.087	**AgeAccelRes**	0.91	(0.79, 1.01)	0.115

AgeAccelDiff, epigenetic age acceleration measured as departure of DNAmAge from chronologic age; AgeAccelRes, epigenetic age acceleration as residuals by regressing DNAmAge on chronologic age; CI, confidence interval; DNAmAge, CRC, colorectal cancer; DNA methylation–based marker of aging; OR, odds ratio; TCGA, The Cancer Genomic Atlas.
† ORs obtained from univariate analyses.

© 2025. Jung SY et al. Published online at http://www.aginganddisease.org/EN/10.14336/AD.2025.0099

Supplementary Table 8. TCGA Levine's clock: Validation tests. Logistic regression results for the DNAmAge/AgeAccelDiff AgeAccelRes predicting CRC

< Overall >				< Females >			
**Analysis**	**OR†**	**95% CI**	***P***	**Analysis**	**OR†**	**95% CI**	***P***
**DNAmAge**	**1.06**	**1.01, 1.12**	**0.037**	**DNAmAge**	1.07	1.01, 1.24	0.129
**AgeAccelDiff**	**1.27**	**1.09, 1.85**	**0.049**	**AgeAccelDiff**	1.18	1.04, 1.67	0.101
**AgeAccelRes**	**1.17**	**1.06, 1.41**	**0.022**	**AgeAccelRes**	1.18	1.03, 1.64	0.120

AgeAccelDiff, epigenetic age acceleration measured as departure of DNAmAge from chronologic age; AgeAccelRes, epigenetic age acceleration as residuals by regressing DNAmAge on chronologic age; CI, confidence interval; CRC, colorectal cancer; DNAmAge, DNA methylation–based marker of aging; OR, odds ratio; TCGA, The Cancer Genomic Atlas. Numbers in bold face are statistically significant.
† ORs obtained from univariate analyses.

Supplementary Table 9. GSE199057 Horvath's clock: Validation tests. Logistic regression results for the DNAmAge/AgeAccelDiff AgeAccelRes predicting CRC.

**Tumor tissues vs. adjacent normal tissues**							
< Overall >				< Females >			
**Analysis**	**OR†**	**95% CI**	***P***	**Analysis**	**OR†**	**95% CI**	***P***
**DNAmAge**	**0.90**	**(0.85, 0.95)**	**0.0002**	**DNAmAge**	**0.87**	**(0.77, 0.96)**	**0.013**
**AgeAccelDiff**	**0.90**	**(0.85, 0.95)**	**0.001**	**AgeAccelDiff**	**0.91**	**(0.82, 0.98)**	**0.031**
**AgeAccelRes**	**0.87**	**(0.80, 0.93)**	**0.0001**	**AgeAccelRes**	**0.84**	**(0.72, 0.94)**	**0.011**
**Tumor tissues vs. normal tissues from patients without cancer**							
< Overall >				< Females >			
**Analysis**	**OR†**	**95% CI**	***P***	**Analysis**	**OR†**	**95% CI**	***P***
**DNAmAge**	**0.91**	**(0.86, 0.96)**	**0.002**	**DNAmAge**	0.94	(0.86, 1.02)	0.170
**AgeAccelDiff**	**0.92**	**(0.87, 0.97)**	**0.004**	**AgeAccelDiff**	0.93	(0.85, 1.00)	0.084
**AgeAccelRes**	**0.89**	**(0.83, 0.95)**	**0.001**	**AgeAccelRes**	0.92	(0.82, 1.00)	0.096

AgeAccelDiff, epigenetic age acceleration measured as departure of DNAmAge from chronologic age; AgeAccelRes, epigenetic age acceleration as residuals by regressing DNAmAge on chronologic age; CI, confidence interval; CRC, colorectal cancer; DNAmAge, DNA methylation–based marker of aging; OR, odds ratio. Numbers in bold face are statistically significant.
† ORs obtained from univariate analyses.

© 2025. Jung SY et al. Published online at http://www.aginganddisease.org/EN/10.14336/AD.2025.0099

Supplementary Table 10. GSE199057 Hannum's clock: Validation tests. Logistic regression results for the DNAmAge/AgeAccelDiff AgeAccelRes predicting CRC.

**Tumor tissues vs. adjacent normal tissues**							
**< Overall>**				**< Females >**			
**Analysis**	**OR†**	**95% CI**	***P***	**Analysis**	**OR†**	**95% CI**	***P***
**DNAmAge**	1.03	(1.00, 1.06)	0.065	**DNAmAge**	1.01	(0.96, 1.07)	0.578
**AgeAccelDiff**	1.02	(1.00, 1.05)	0.066	**AgeAccelDiff**	1.01	(0.97, 1.06)	0.623
**AgeAccelRes**	1.03	(1.00, 1.06)	0.057	**AgeAccelRes**	1.02	(0.96, 1.07)	0.571
**Tumor tissues vs. normal tissues from patients without cancer**							
**< Overall >**				**< Females >**			
**Analysis**	**OR†**	**95% CI**	***P***	**Analysis**	**OR†**	**95% CI**	***P***
**DNAmAge**	**0.95**	**(0.91, 0.99)**	**0.012**	**DNAmAge**	**0.90**	**(0.81, 0.97)**	**0.022**
**AgeAccelDiff**	**0.96**	**(0.92, 0.99)**	**0.016**	**AgeAccelDiff**	**0.89**	**(0.80, 0.96)**	**0.016**
**AgeAccelRes**	**0.95**	**(0.91, 0.98)**	**0.010**	**AgeAccelRes**	**0.87**	**(0.77, 0.96)**	**0.016**

AgeAccelDiff, epigenetic age acceleration measured as departure of DNAmAge from chronologic age; AgeAccelRes, epigenetic age acceleration as residuals by regressing DNAmAge on chronologic age; CI, confidence interval; CRC, colorectal cancer; DNAmAge, DNA methylation–based marker of aging; OR, odds ratio. Numbers in bold face are statistically significant.

† ORs obtained from univariate analyses.

Supplementary Table 11. GSE199057 Levine's clock: Validation tests. Logistic regression results for the DNAmAge/AgeAccelDiff

© 2025. Jung SY et al. Published online at http://www.aginganddisease.org/EN/10.14336/AD.2025.0099

Supplementary Data

AgeAccelRes predicting CRC.

Tumor tissues vs. adjacent normal tissues							
**< Overall >**				**< Females >**			
**Analysis**	**OR†**	**95% CI**	***P***	**Analysis**	**OR†**	**95% CI**	***P***
**DNAmAge**	**1.08**	**(1.05, 1.12)**	**1.03E-05**	**DNAmAge**	**1.07**	**(1.03, 1.14)**	**0.008**
**AgeAccelDiff**	**1.09**	**(1.05, 1.14)**	**3.25E-05**	**AgeAccelDiff**	**1.07**	**(1.03, 1.15)**	**0.011**
**AgeAccelRes**	**1.09**	**(1.05, 1.14)**	**2.36E-05**	**AgeAccelRes**	**1.08**	**(1.03, 1.15)**	**0.009**
**Tumor tissues vs. normal tissues from patients without cancer**							
**< Overall >**				**< Females >**			
**Analysis**	**OR†**	**95% CI**	***P***	**Analysis**	**OR†**	**95% CI**	***P***
**DNAmAge**	**1.06**	**(1.03, 1.11)**	**0.0005**	**DNAmAge**	**1.06**	**(1.01, 1.13**	**0.033**
**AgeAccelDiff**	**1.07**	**(1.04, 1.12)**	**0.001**	**AgeAccelDiff**	1.05	(1.00, 1.12)	0.074
**AgeAccelRes**	**1.08**	**(1.04, 1.13)**	**0.001**	**AgeAccelRes**	1.06	(1.01, 1.14)	0.050

AgeAccelDiff, epigenetic age acceleration measured as departure of DNAmAge from chronologic age; AgeAccelRes, epigenetic age acceleration as residuals by regressing DNAmAge on chronologic age; CI, confidence interval; CRC, colorectal cancer; DNAmAge, DNA methylation–based marker of aging; OR, odds ratio. Numbers in bold face are statistically significant.

† ORs obtained from univariate analyses.

© 2025. Jung SY et al. Published online at http://www.aginganddisease.org/EN/10.14336/AD.2025.0099

Supplementary Data

Overall

A. Alcohol: DNAmAge, *p* = 0.966

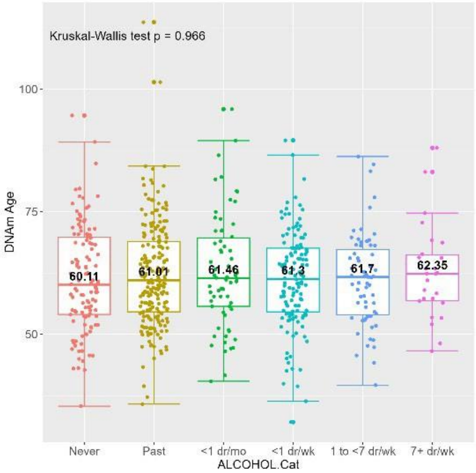

B. Alcohol: AgeAccelDiff, *p* = 0.328

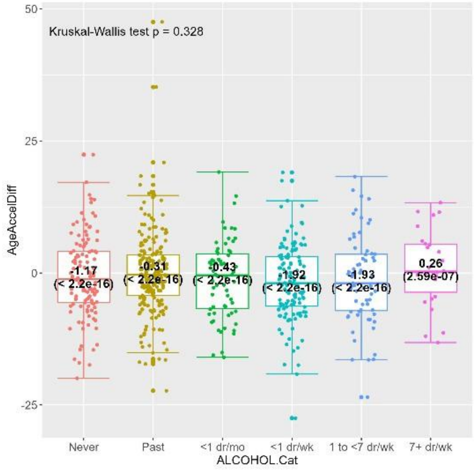

C. Alcohol: IEAA *p* = 0.908

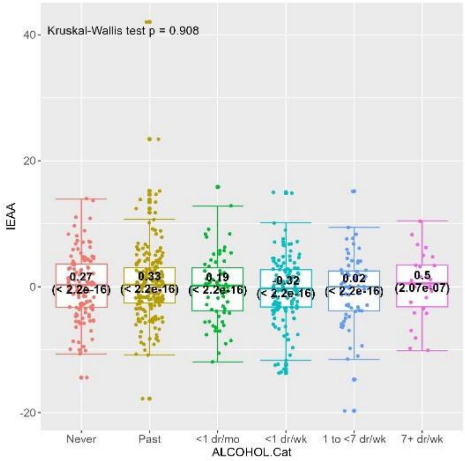

Overall

D. Years of regular smoking: DNAmAge, *p* = 0.329

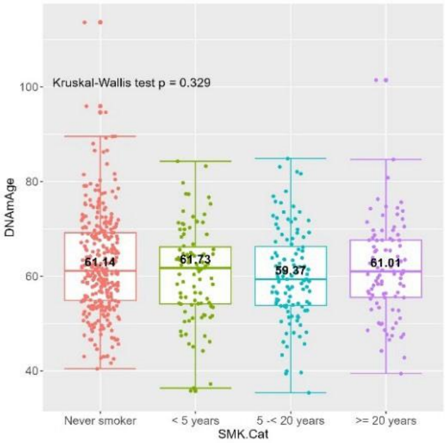

E. Years of regular smoking:AgeAccelDiff, *p* = 0.845

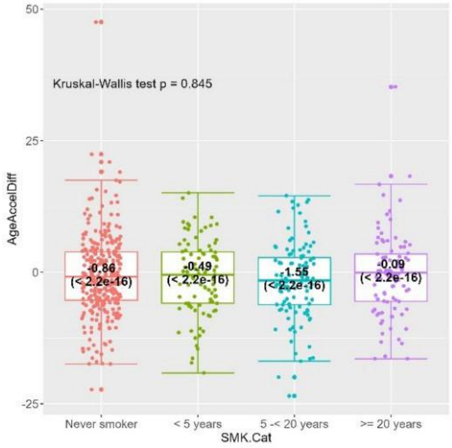

F. Years of regular smoking: IEAA, *p* = 0.439

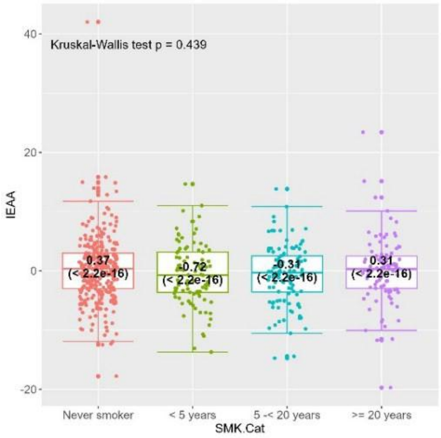

© 2025. Jung SY et al. Published online at http://www.aginganddisease.org/EN/10.14336/AD.2025.0099

Supplementary Data

Overall

G. HEI-2015, whole fruits: DNAmAge, $p = 0.242$

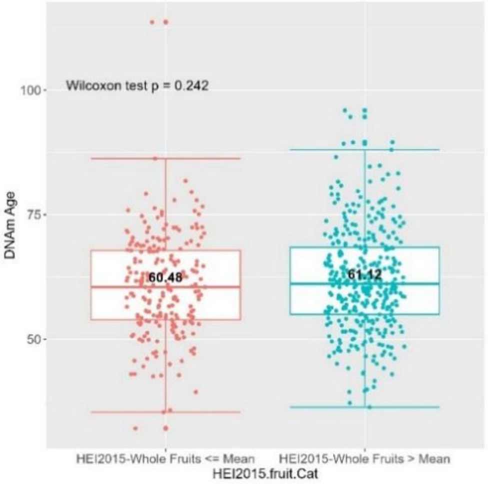

H. HEI-2015, whole fruits:AgeAccelDiff, $p = 0.342$

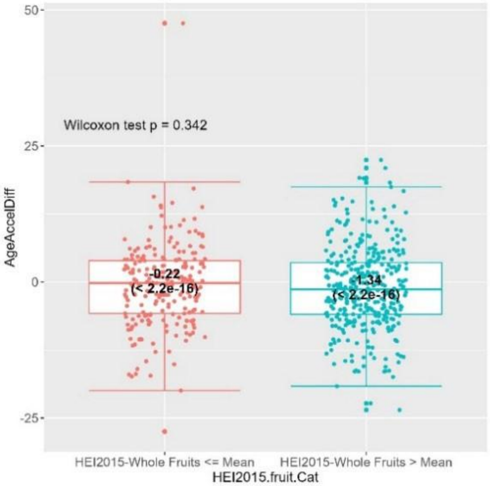

I. HEI-2015, whole fruits: IEAA, $p = 0.739$

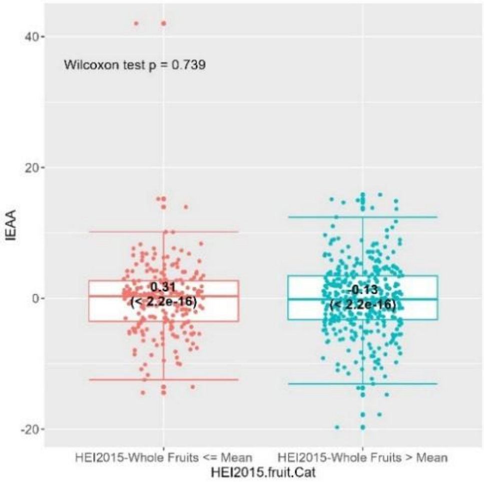

Among participants without CRC

J. HEI-2015, fatty acids: DNAmAge, $p = 0.07$

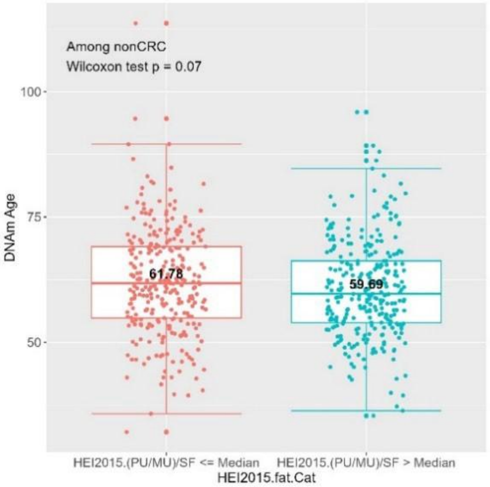

K. HEI-2015, fatty acids: AgeAccelDiff, $p = 0.056$

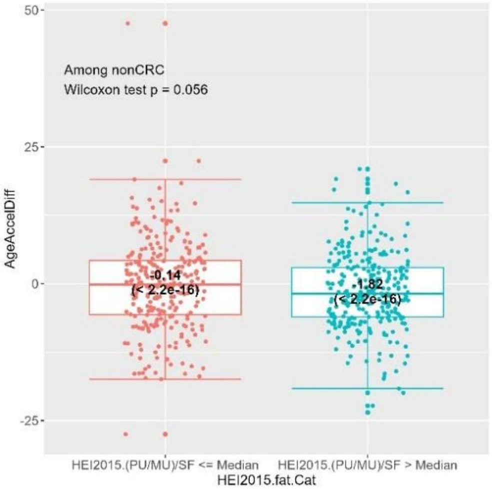

L. HEI-2015, fatty acids: IEAA, $p = 0.438$

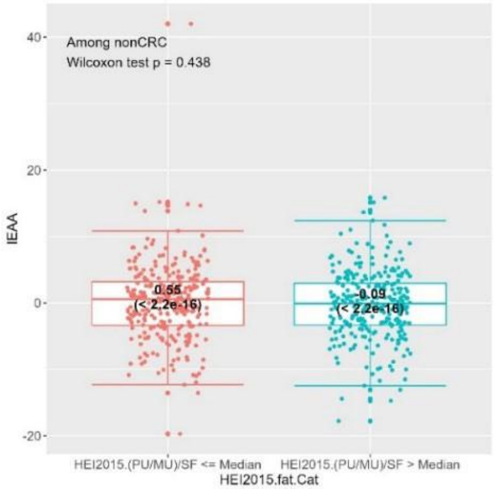

© 2025. Jung SY et al. Published online at http://www.aginganddisease.org/EN/10.14336/AD.2025.0099

Supplementary Data

Among patients with CRC

M. HEI-2015, fatty acids: DNAmAge, $p = 0.456$

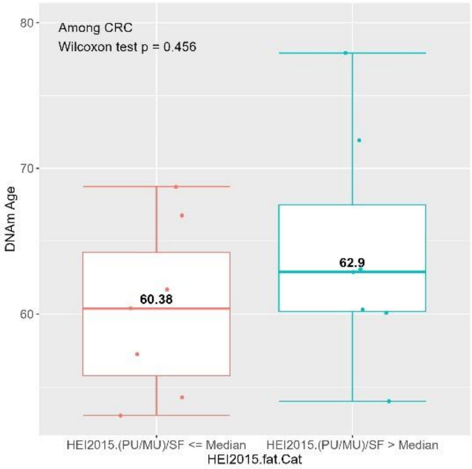

N. HEI-2015, fatty acids:AgeAccelDiff, $p = 0.259$

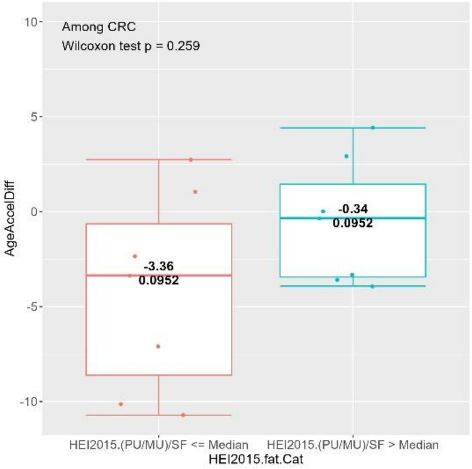

O. HEI-2015, fatty acids: IEAA, $p = 0.318$

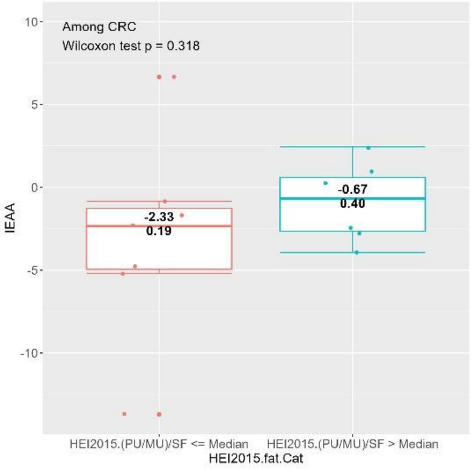

Overall

P. Physical activity: DNAmAge, $p = 0.013$

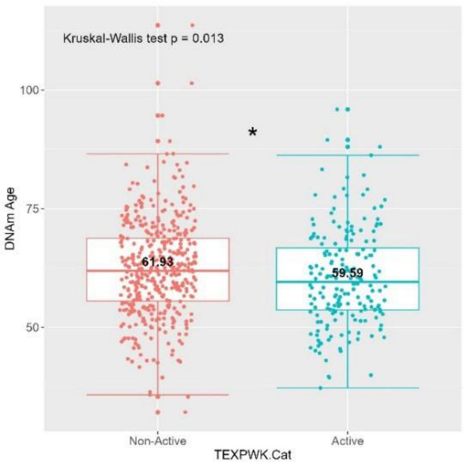

Q. Physical activity: AgeAccelDiff, $p = 0.004$

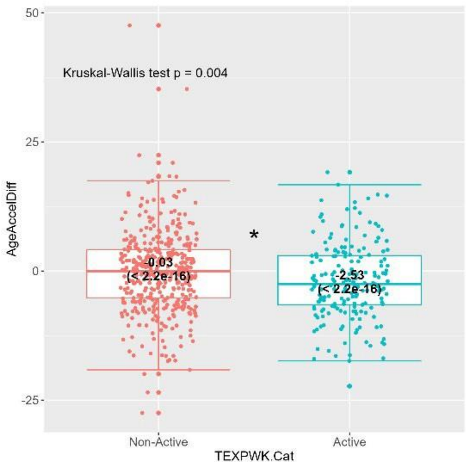

R. Physical activity: IEAA, $p = 0.011$

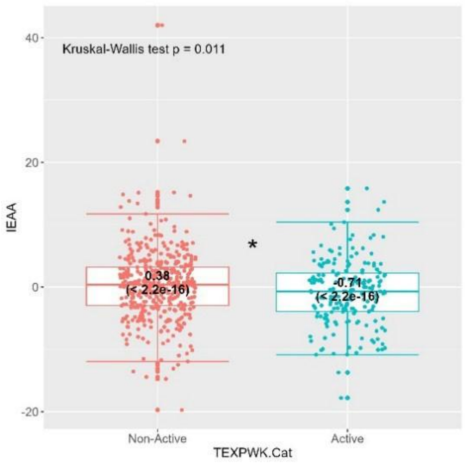

© 2025. Jung SY et al. Published online at http://www.aginganddisease.org/EN/10.14336/AD.2025.0099

Supplementary Data

Overall

S. Oophorectomy: DNAmAge, $p = 0.524$

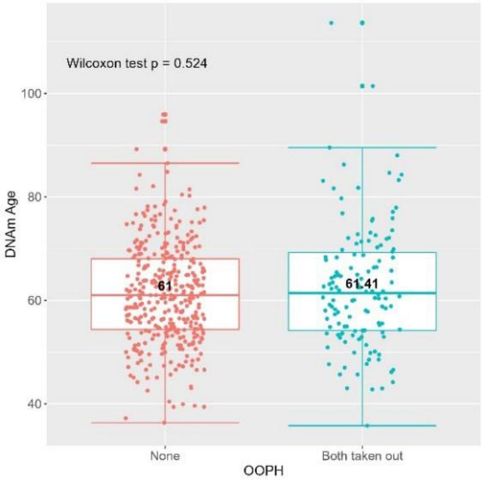

T. Oophorectomy: AgeAccelDiff, $p = 0.379$

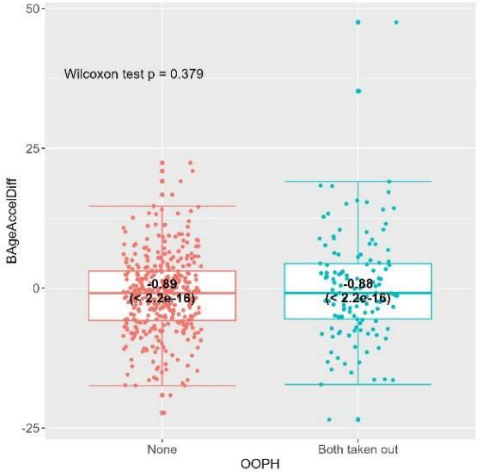

U. Oophorectomy: IEAA, $p = 0.857$

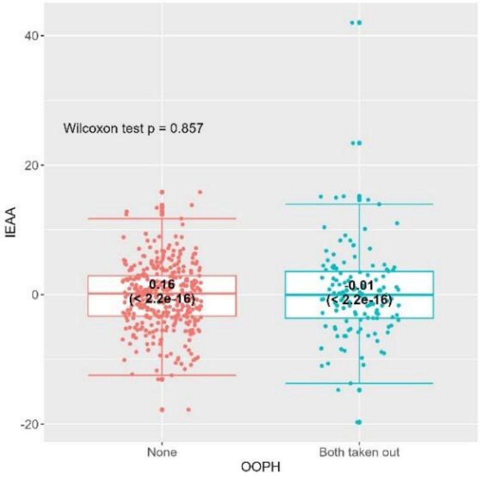

Overall

V. E only: DNAmAge, $p = 0.096$

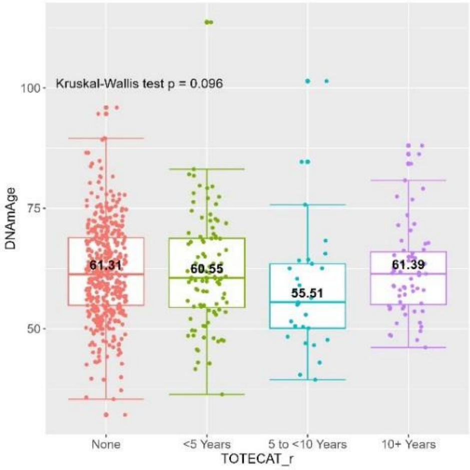

W. E only: AgeAccelDiff, $p = 0.211$

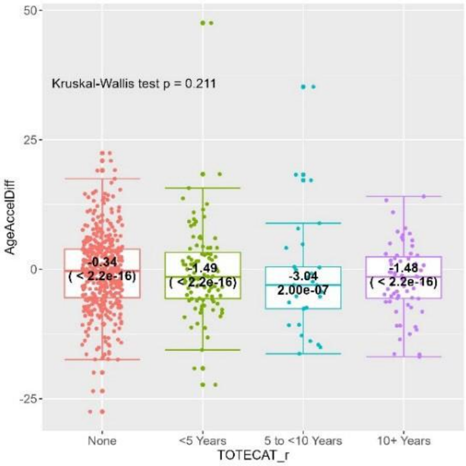

X. E only: IEAA, $p = 0.534$

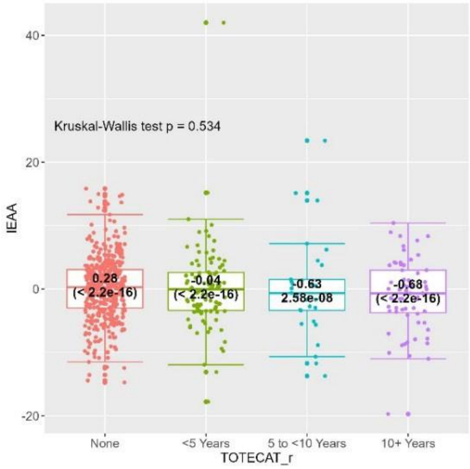

© 2025. Jung SY et al. Published online at http://www.aginganddisease.org/EN/10.14336/AD.2025.0099

Supplementary Data

Overall

Y. E plus P: DNAmAge, p = 0.051

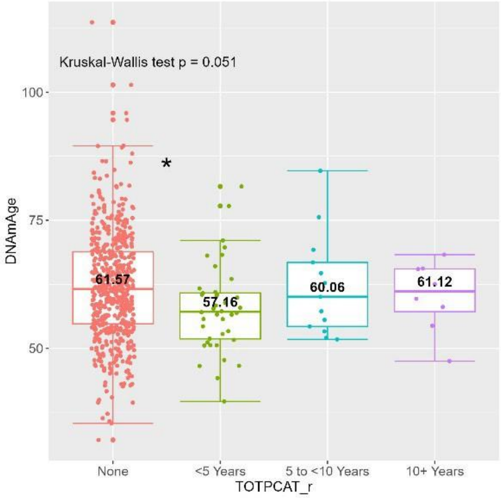

Z. E plus P: AgeAccelDiff, p = 0.857

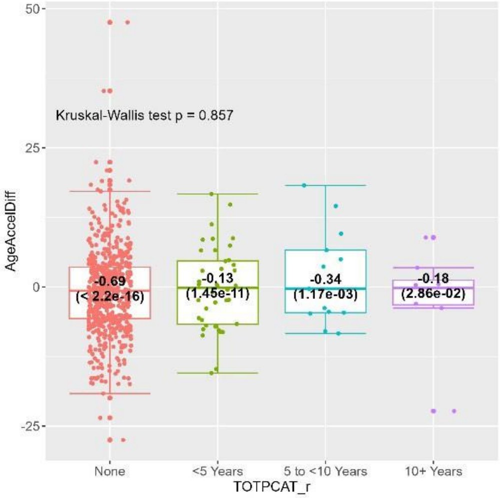

AA. E plus P: IEAA, p = 0.933

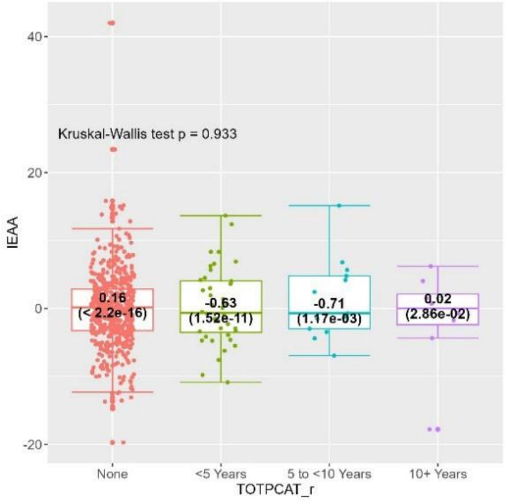

Supplementary Figure 1. Horvath's clock: distribution of DNAmAge/AgeAccelDiff/IEAA by selected CRC risk factors in overall participants. (AgeAccelDiff, epigenetic age acceleration as departure of DNAmAge from chronologic age; CRC, colorectal cancer; DNAmAge, DNA methylation–based marker of aging; E only, exogenous estrogen only; E plus P, E plus progestin; HEI-2015, Healthy Eating Index-2015; IEAA, intrinsic epigenetic age acceleration as residuals adjusted for cell composition.)

© 2025. Jung SY et al. Published online at http://www.aginganddisease.org/EN/10.14336/AD.2025.0099

Supplementary Data

Overall

A. Alcohol: DNAmAge, $p = 0.722$

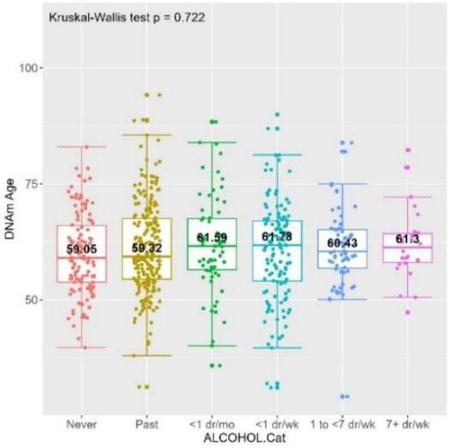

B. Alcohol: AgeAccelDiff, $p = 0.261$

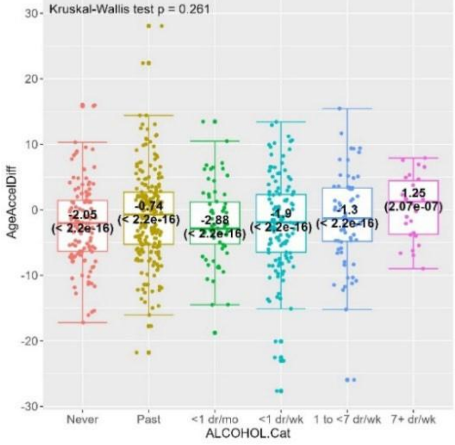

C. Alcohol: IEAA, $p = 0.745$

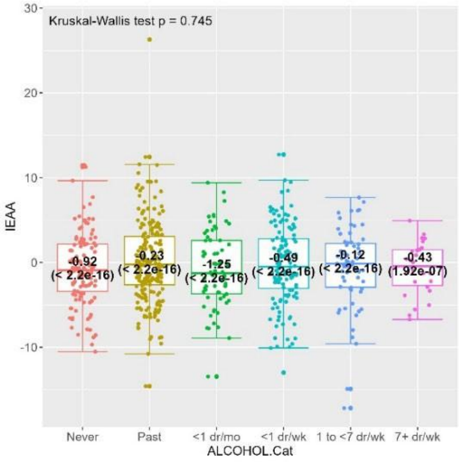

Overall

D. Years of regular smoking: DNAmAge, $p = 0.192$

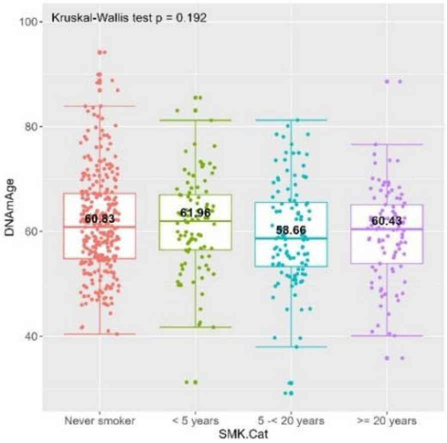

E. Years of regular smoking:AgeAccelDiff, $p = 0.072$

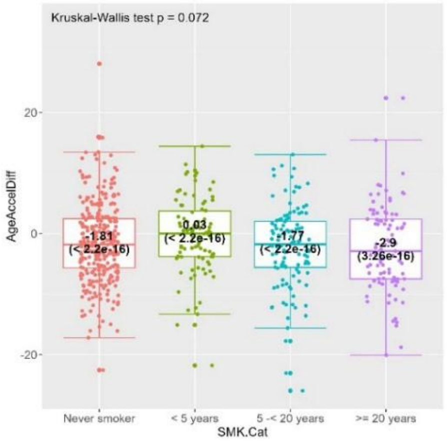

F. Years of regular smoking: IEAA, $p = 0.031$

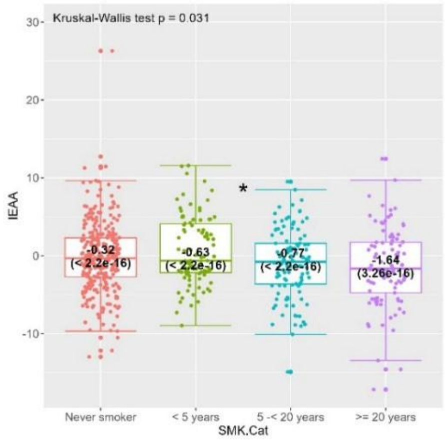

© 2025. Jung SY et al. Published online at http://www.aginganddisease.org/EN/10.14336/AD.2025.0099

Supplementary Data

Overall

G. HEI-2015, vegetables: DNAmAge, $p = 0.275$

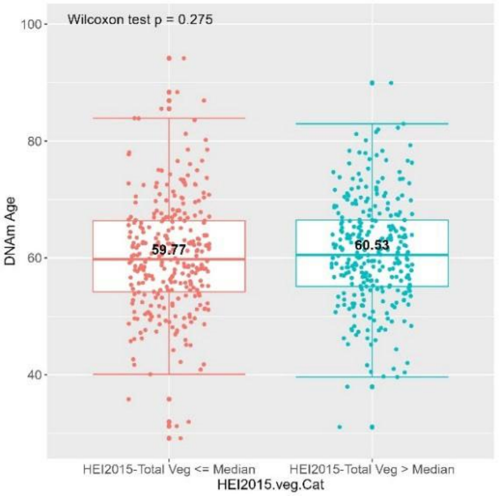

H. HEI-2015, vegetables:AgeAccelDiff, $p = 0.251$

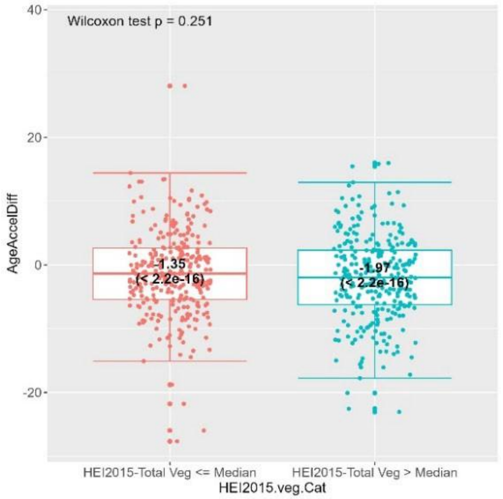

I. HEI-2015, vegetables: IEAA, $p = 0.173$

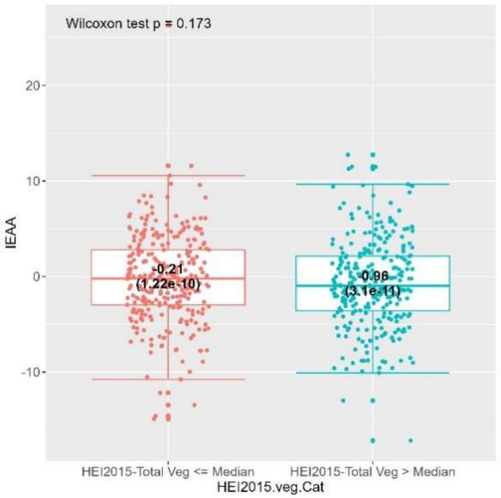

Among participants without CRC

J. HEI-2015, fatty acids: DNAmAge, $p = 0.004$

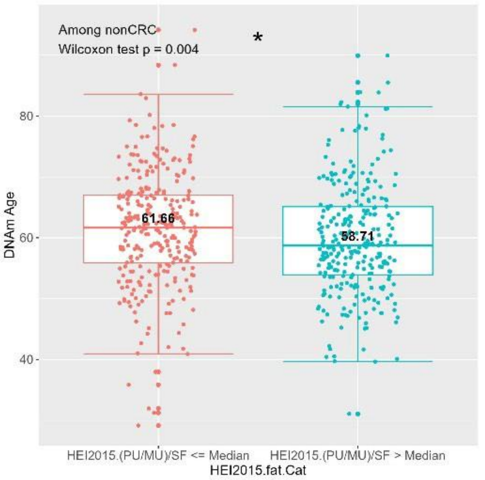

K. HEI-2015, fatty acids:AgeAccelDiff, $p = 0.015$

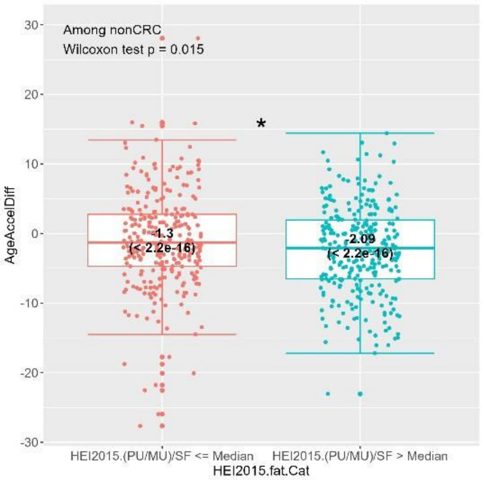

L. HEI-2015, fatty acids: IEAA, $p = 0.236$

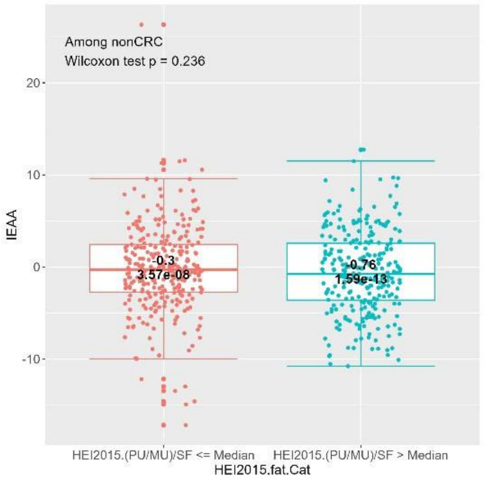

© 2025. Jung SY et al. Published online at http://www.aginganddisease.org/EN/10.14336/AD.2025.0099

Supplementary Data

Among patients with CRC

M. HEI-2015, fatty acids: DNAmAge, *p* = 0.71

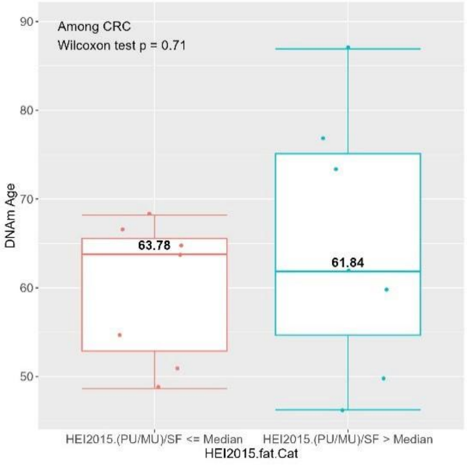

N. HEI-2015, fatty acids:AgeAccelDiff, *p* = 0.318

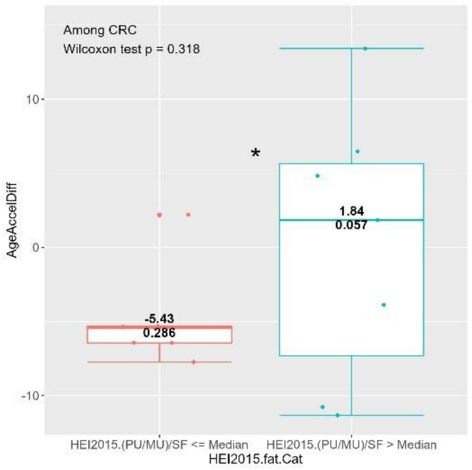

O. HEI-2015, fatty acids: IEAA, *p* = 0.128

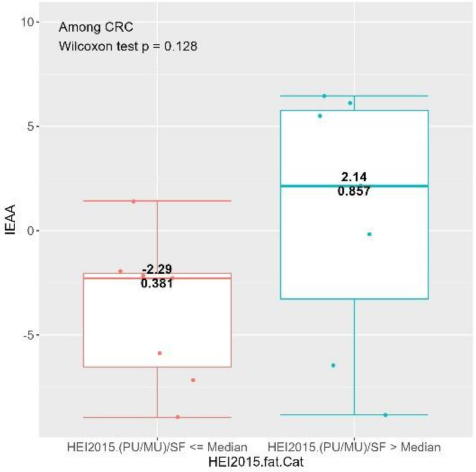

Overall

P. Physical activity: DNAmAge, *p* = 0.106

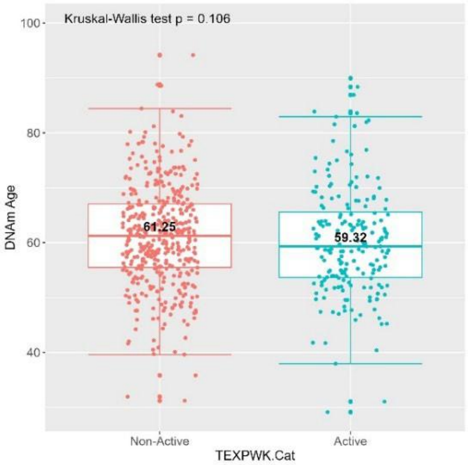

Q. Physical activity: AgeAccelDiff, *p* = 0.132

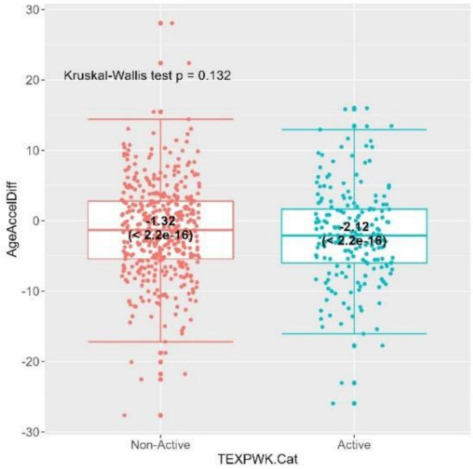

R. Physical activity: IEAA, *p* = 0.286

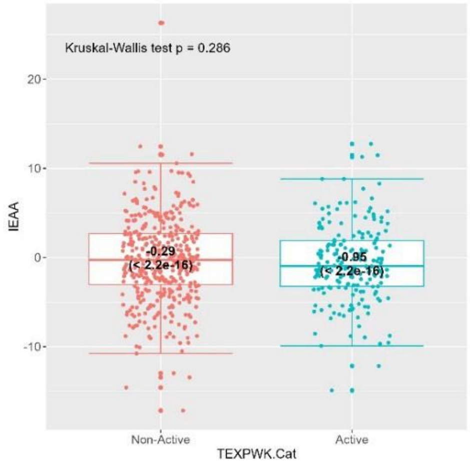

© 2025. Jung SY et al. Published online at http://www.aginganddisease.org/EN/10.14336/AD.2025.0099

Supplementary Data

Among patients with CRC

S. Oophorectomy: DNAmAge, $p = 0.024$

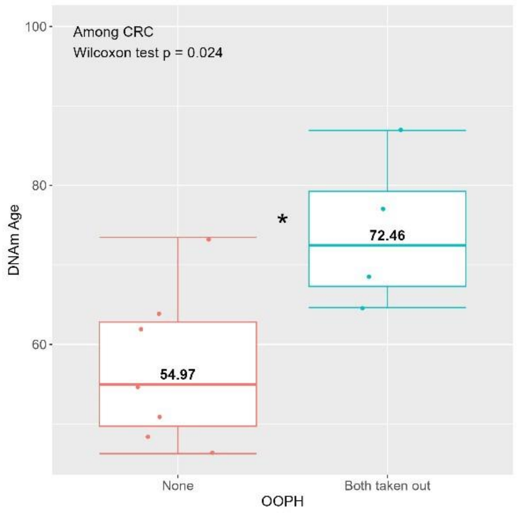

T. Oophorectomy: AgeAccelDiff, $p = 0.23$

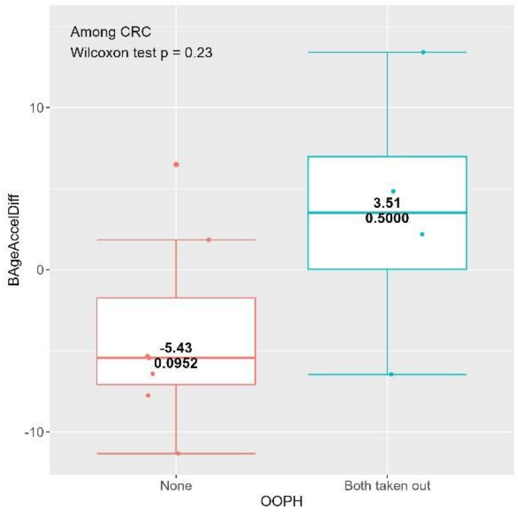

U. Oophorectomy: IEAA, $p = 0.927$

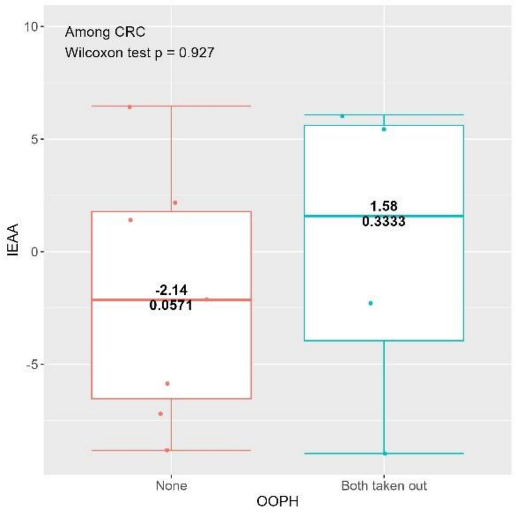

Among patients with CRC

V. E only: DNAmAge, $p = 0.686$

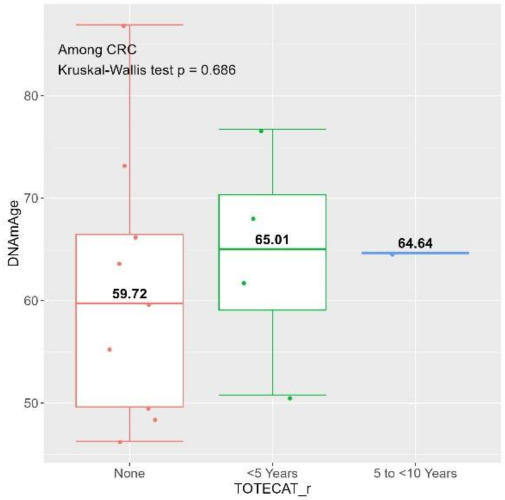

W. E only: AgeAccelDiff, $p = 0.359$

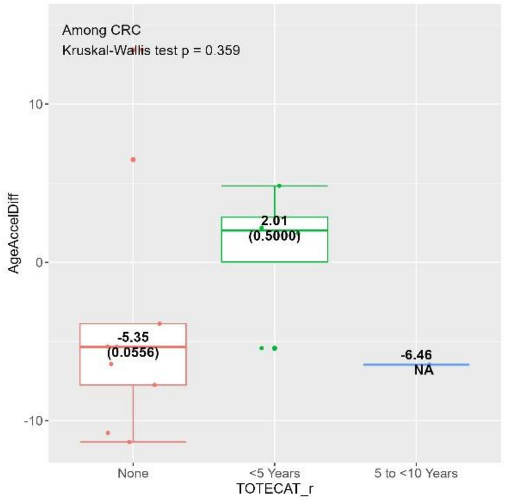

X. E only: IEAA, $p = 0.164$

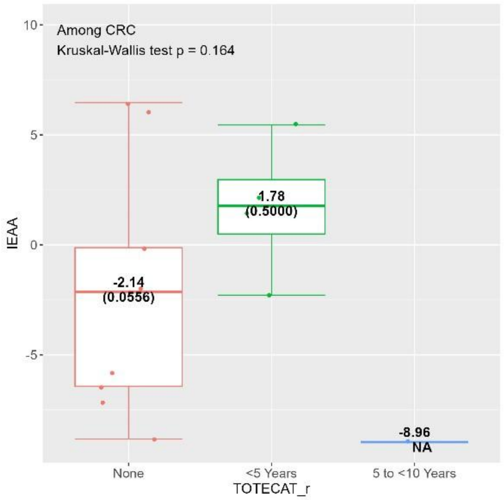

© 2025. Jung SY et al. Published online at http://www.aginganddisease.org/EN/10.14336/AD.2025.0099

Supplementary Data

Overall

Y. E only: DNAmAge, *p* = 0.153

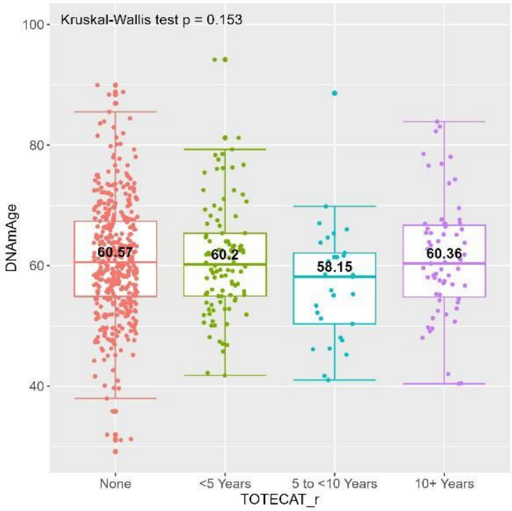

Z. E only: AgeAccelDiff, *p* = 0.195

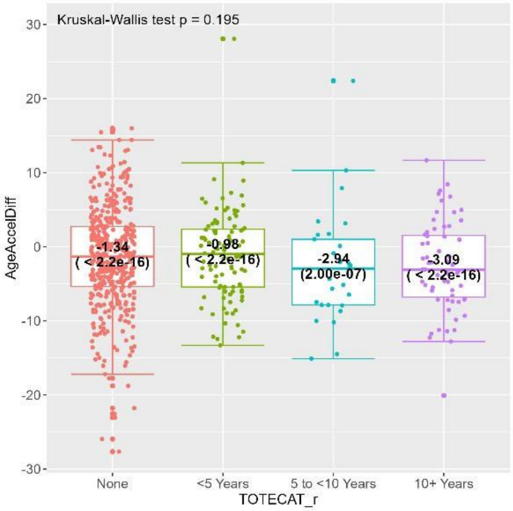

AA. E only: IEAA, *p* = 0.301

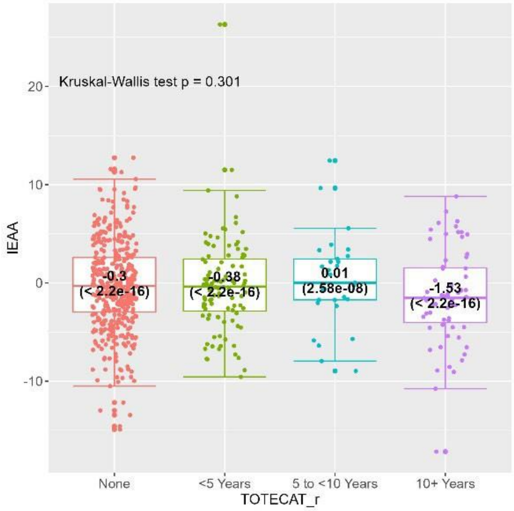

Overall

BB. E plus P: DNAmAge, *p* = 0.007

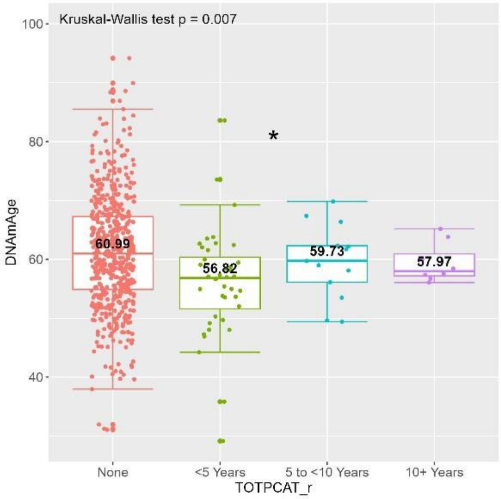

CC. E plus P:AgeAccelDiff, *p* = 0.911

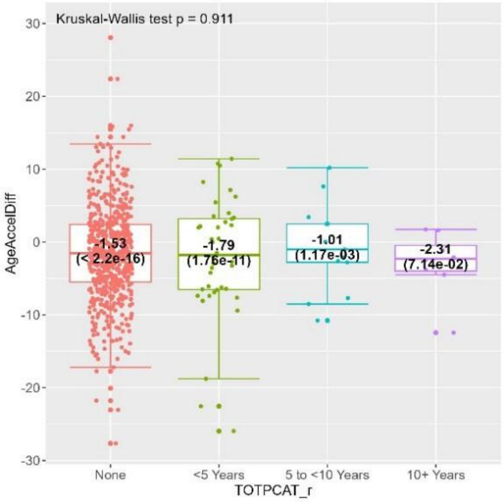

DD. E plus P: IEAA, *p* = 0.871

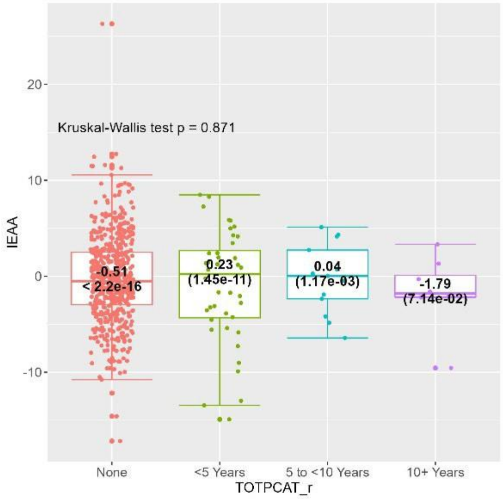

Supplementary Figure 2. Hannum's clock: distribution of DNAmAge/AgeAccelDiff/IEAA by selected CRC risk factors. (AgeAccelDiff, epigenetic age acceleration as departure of DNAmAge from chronologic age; CRC, colorectal cancer; DNAmAge, DNA methylation–based marker of aging; E only, exogenous estrogen only; E plus P, E plus progestin; HEI-2015, Health Eating Index-2015; IEAA, intrinsic epigenetic age acceleration as residuals adjusted for cell composition.)

© 2025. Jung SY et al. Published online at http://www.aginganddisease.org/EN/10.14336/AD.2025.0099

Supplementary Data

Overall

A. Type 2 diabetes: DNAmAge, *p* = 0.147

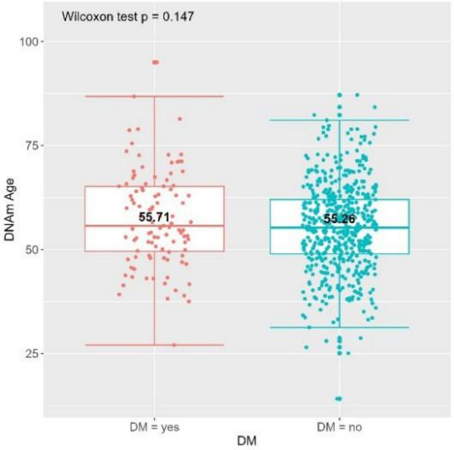

B. Type 2 diabetes: AgeAccelDiff, *p* = 0.198

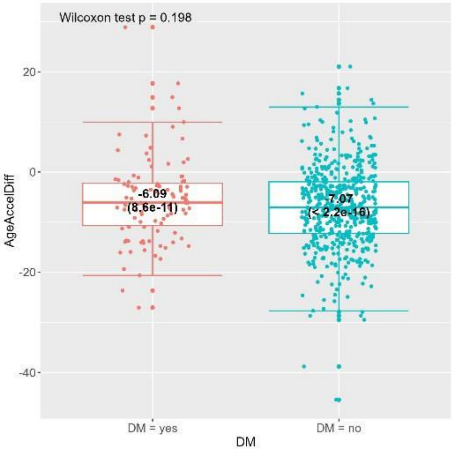

C. Type 2 diabetes: IEAA, *p* = 0.04

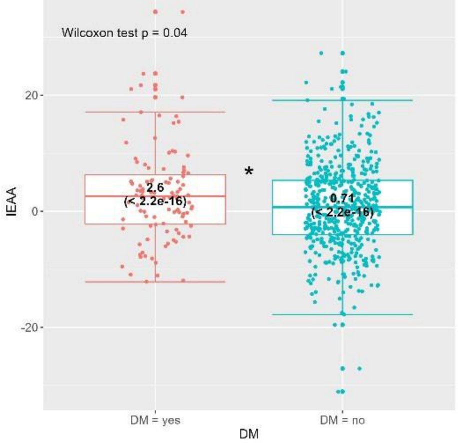

Overall

D. BMI: DNAmAge, *p* = 0.89

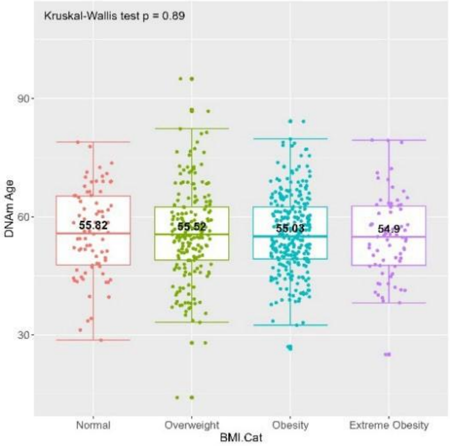

E. BMI: AgeAccelDiff, *p* = 0.078

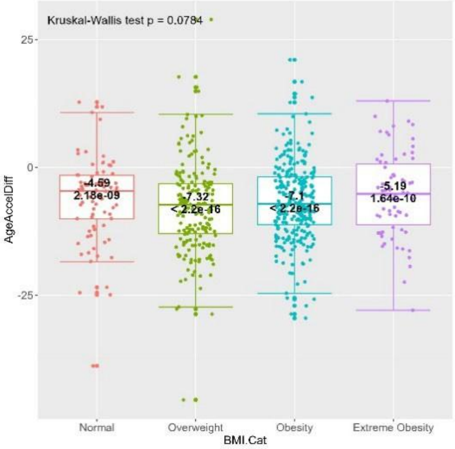

F. BMI: IEAA, *p* = 0.03

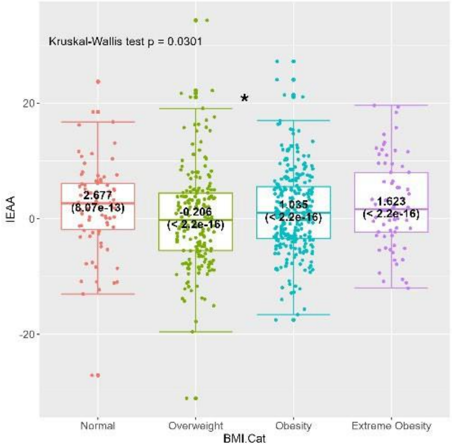

© 2025. Jung SY et al. Published online at http://www.aginganddisease.org/EN/10.14336/AD.2025.0099

Supplementary Data

Overall

G. WHR: DNAmAge, $p = 0.266$

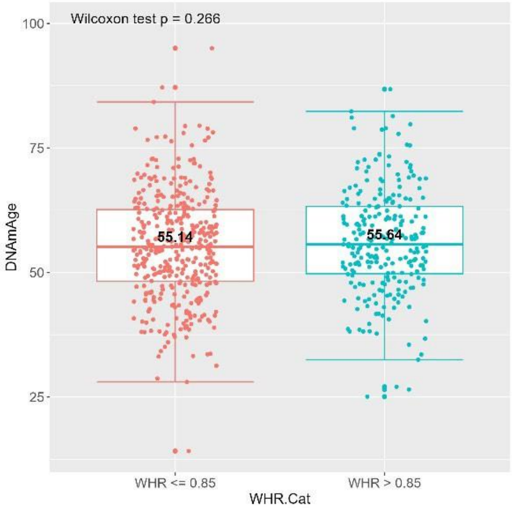

H. WHR: AgeAccelDiff, $p = 0.108$

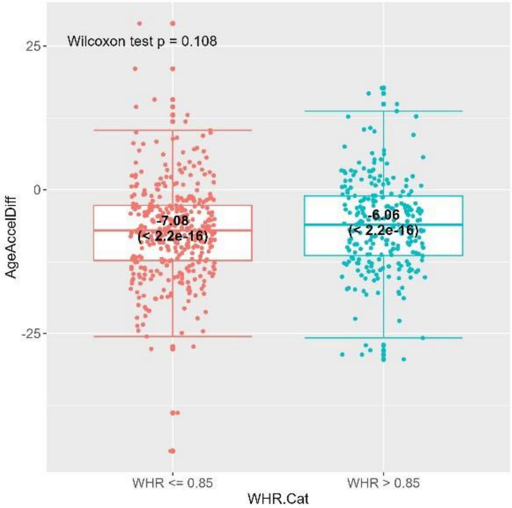

I. WHR: IEAA, $p = 0.044$

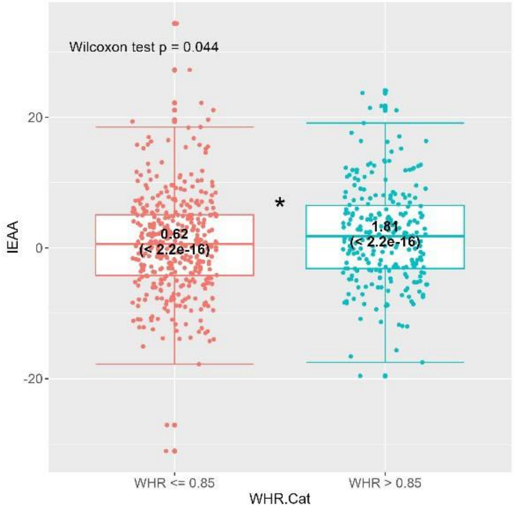

Overall

J. Alcohol: DNAmAge, $p = 0.499$

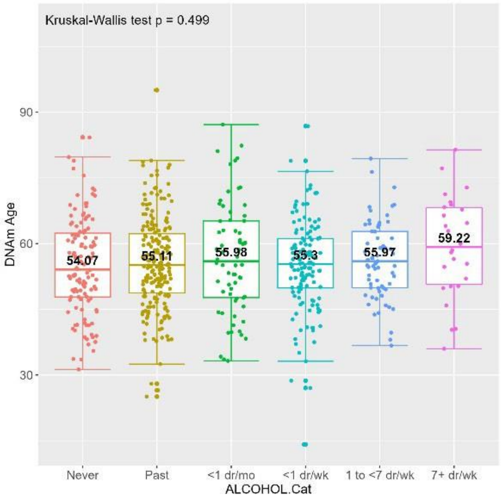

K. Alcohol: AgeAccelDiff, $p = 0.069$

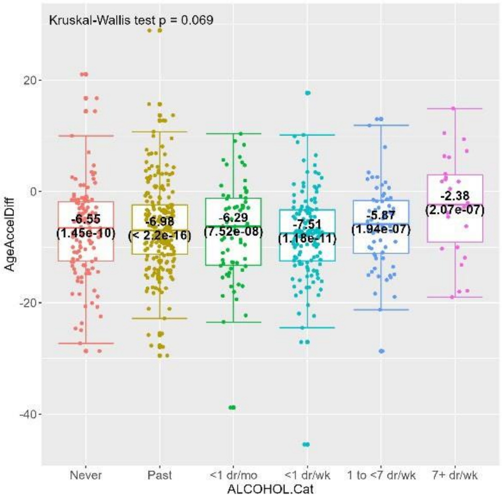

L. Alcohol: IEAA, $p = 0.45$

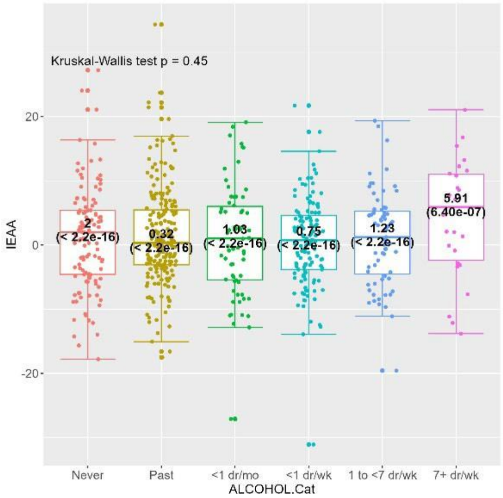

© 2025. Jung SY et al. Published online at http://www.aginganddisease.org/EN/10.14336/AD.2025.0099

Supplementary Data

Among patients with CRC

M. Years of regular smoking: DNAmAge, *p* = 0.758

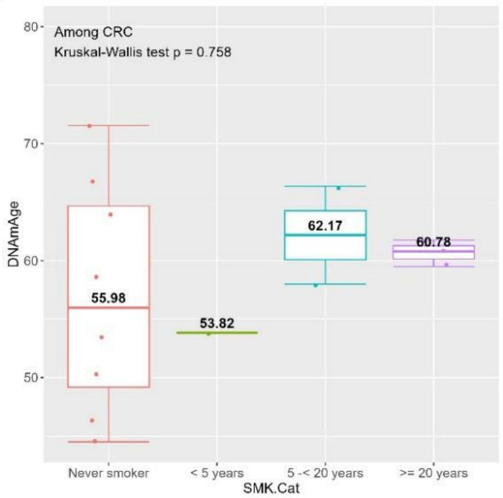

N. Years of regular smoking:AgeAccelDiff, *p* = 0.119

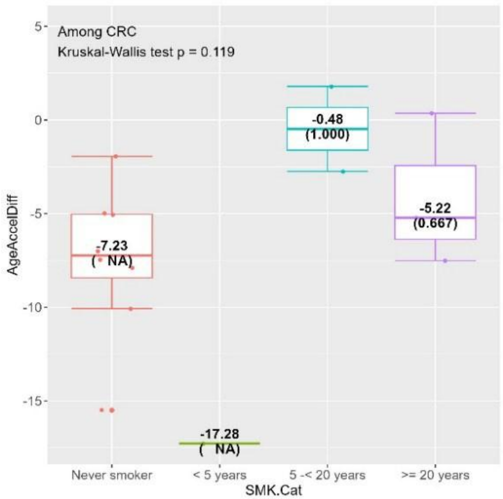

O. Years of regular smoking: IEAA, *p* = 0.079

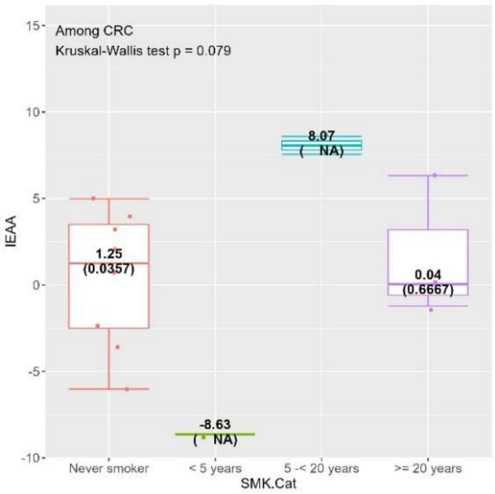

Overall

P. Years of regular smoking: DNAmAge, *p* = 0.254

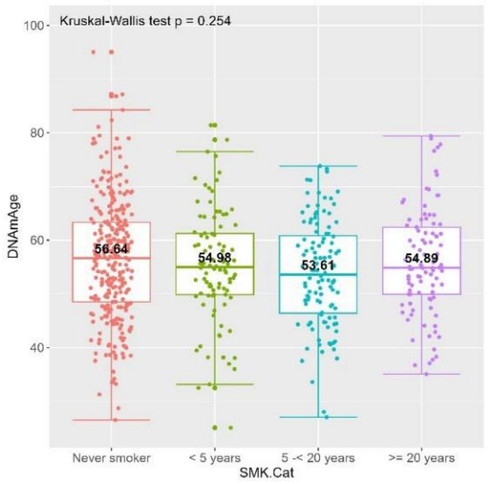

Q. Years of regular smoking:AgeAccelDiff, *p* = 0.536

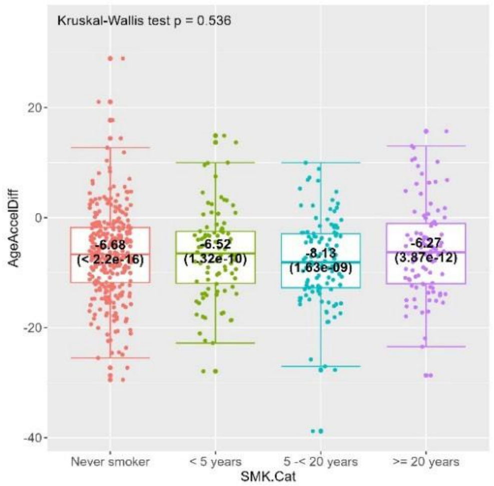

R. Years of regular smoking: IEAA, *p* = 0.283

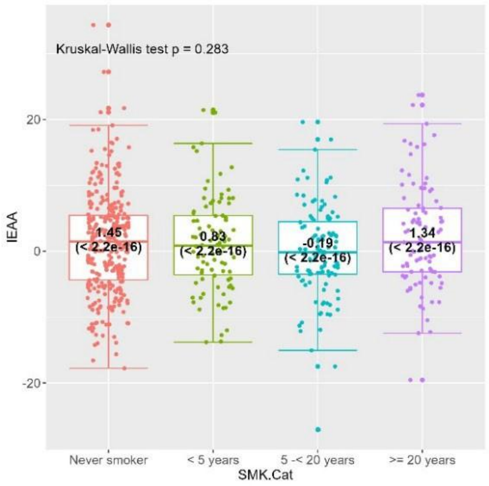

© 2025. Jung SY et al. Published online at http://www.aginganddisease.org/EN/10.14336/AD.2025.0099

Supplementary Data

Among patients with CRC

S. HEI-2015, whole fruits: DNAmAge, $p = 0.0175$

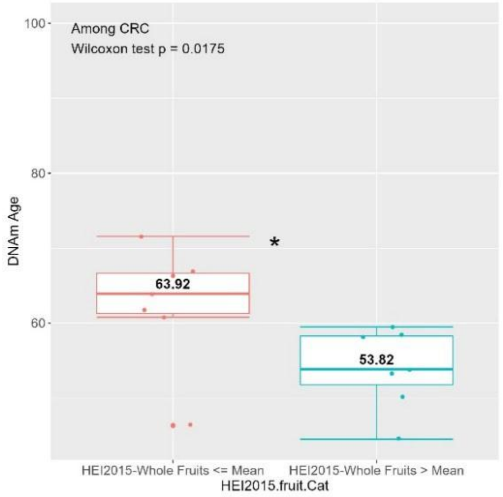

T. HEI-2015, whole fruits:AgeAccelDiff, $p = 0.318$

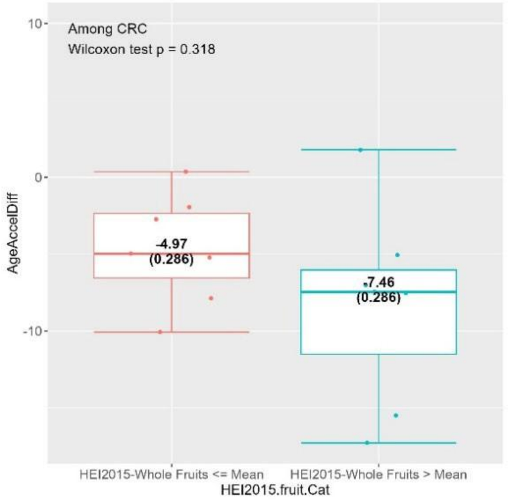

U. HEI-2015, whole fruits: IEAA, $p = 0.259$

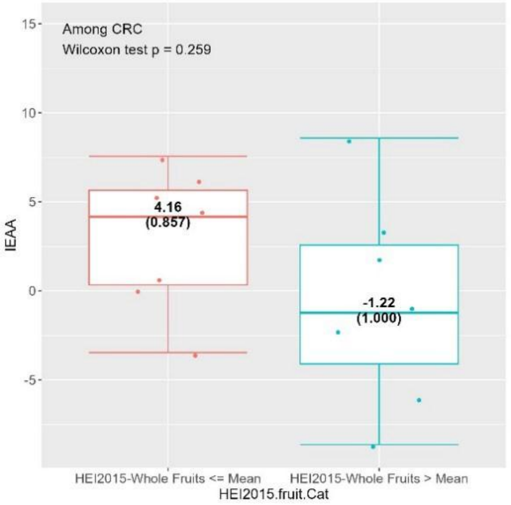

Among participants without CRC

V. HEI-2015, fatty acids: DNAmAge, $p = 0.022$

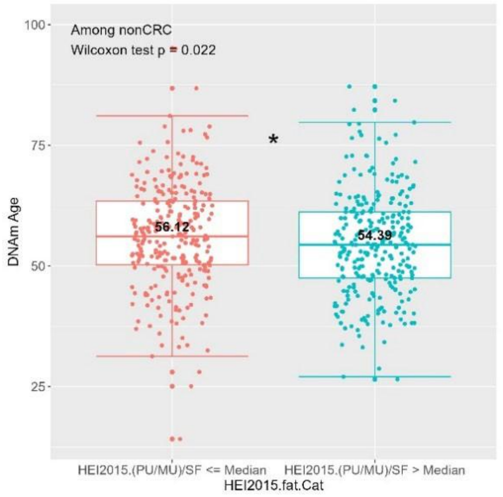

W. HEI-2015, fatty acids:AgeAccelDiff, $p = 0.01$

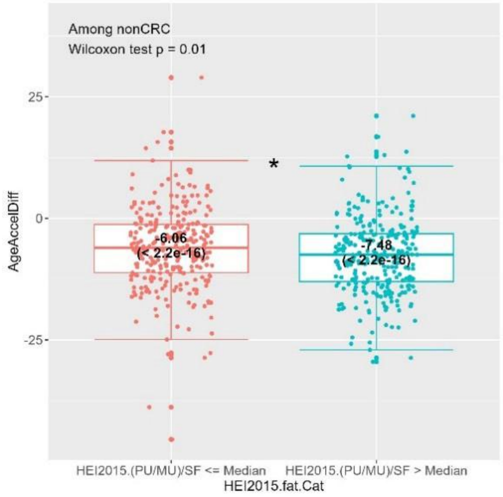

X. HEI-2015, fatty acids: IEAA, $p = 0.12$

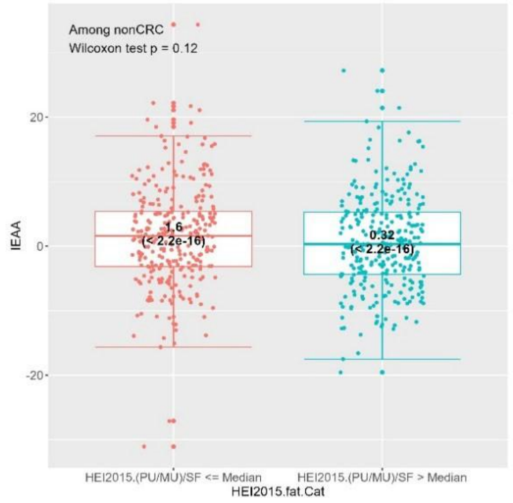

© 2025. Jung SY et al. Published online at http://www.aginganddisease.org/EN/10.14336/AD.2025.0099

Supplementary Data

Among patients with CRC

Y. HEI-2015, fatty acids: DNAmAge, *p* = 0.805

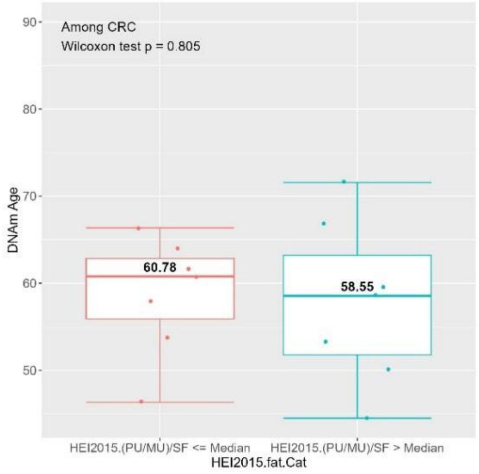

Z. HEI-2015, fatty acids:AgeAccelDiff, *p* = 0.902

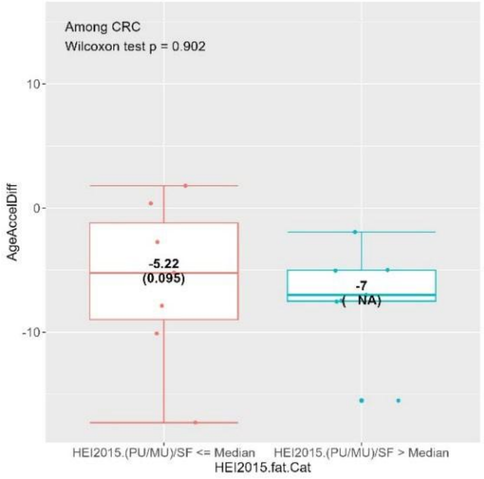

AA. HEI-2015, fatty acids: IEAA, *p* = 0.71

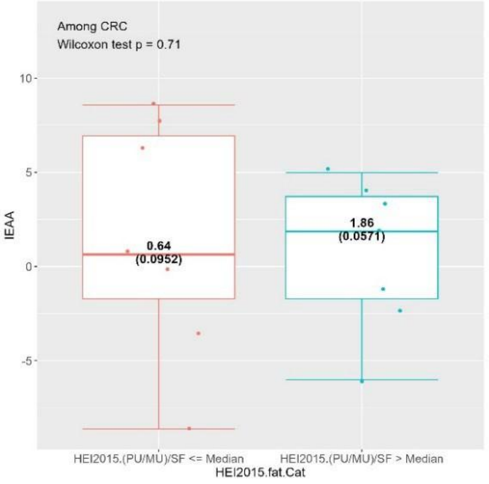

Overall

BB. Physical activity: DNAmAge, *p* = 0.036

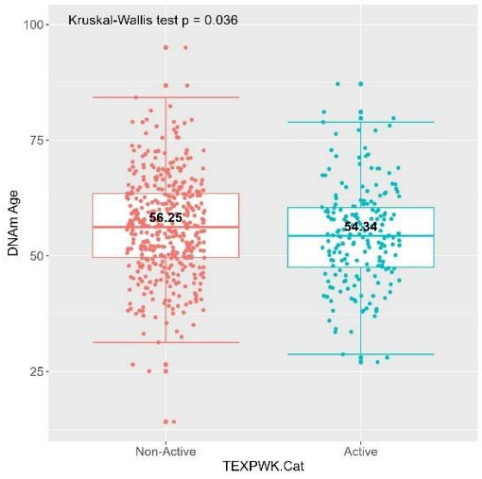

CC. Physical activity: AgeAccelDiff, *p* = 0.019

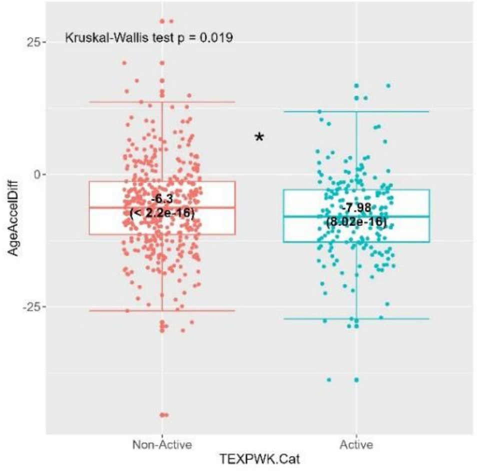

DD. Physical activity: IEAA, *p* = 0.029

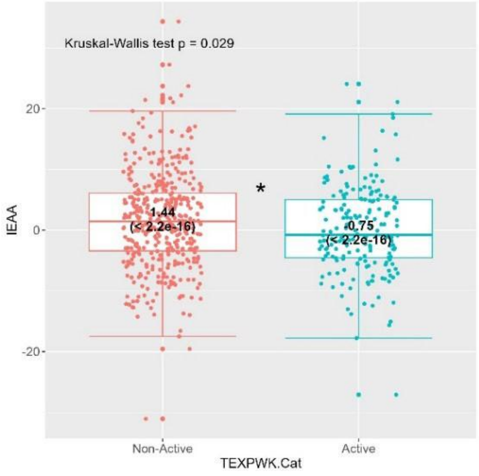

© 2025. Jung SY et al. Published online at http://www.aginganddisease.org/EN/10.14336/AD.2025.0099

Supplementary Data

Among patients with CRC

EE. E only: DNAmAge, *p* = 0.799

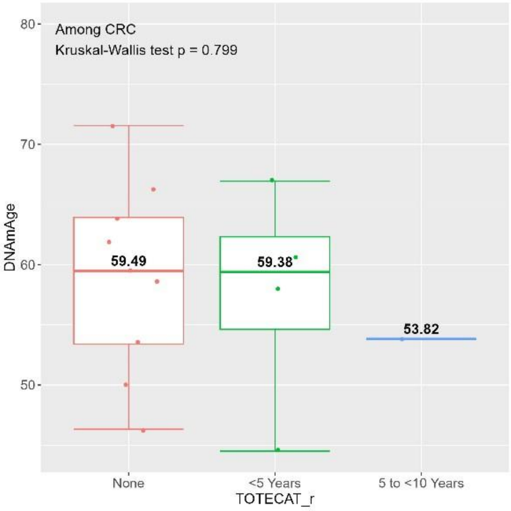

FF. E only: AgeAccelDiff, *p* = 0.262

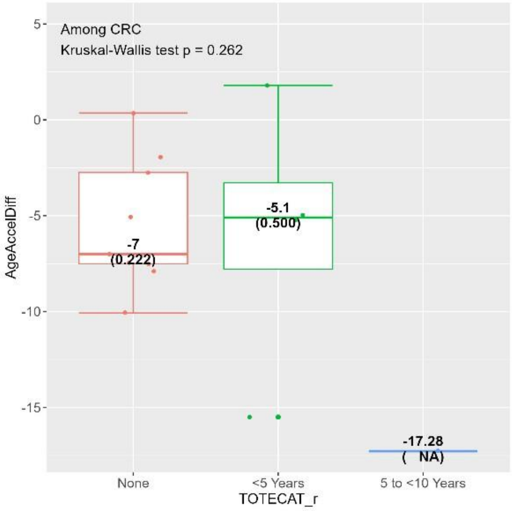

GG. E only: IEAA, *p* = 0.27

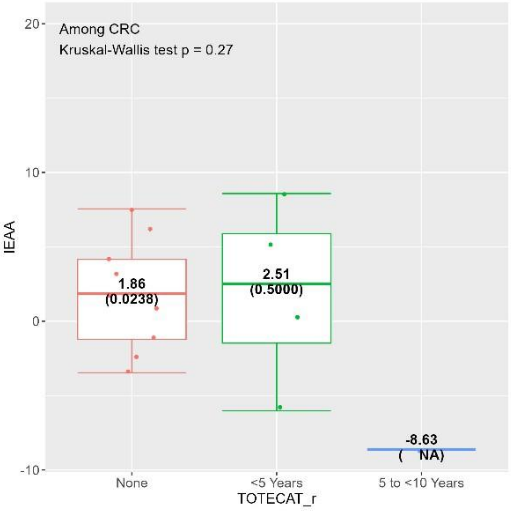

Overall

HH. E only: DNAmAge, *p* = 0.195

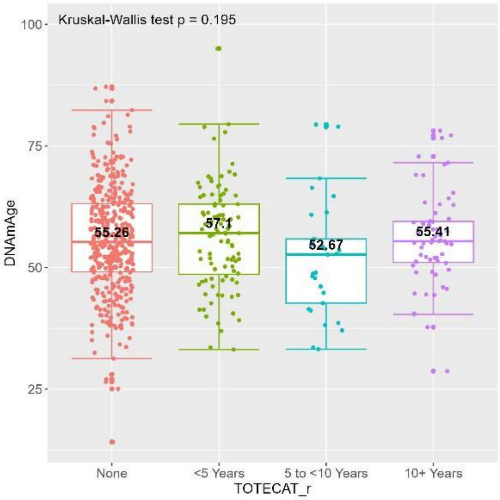

II. E only: AgeAccelDiff, *p* = 0.193

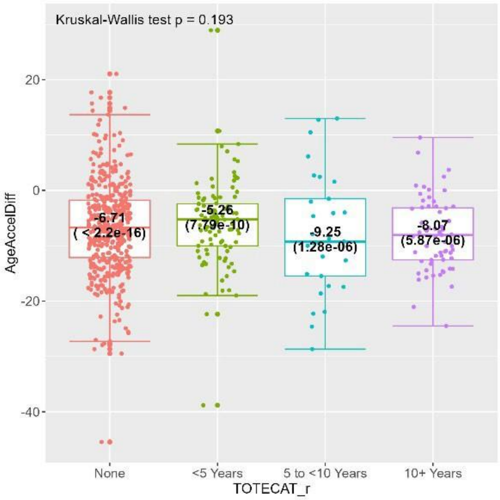

JJ. E only: IEAA, *p* = 0.098

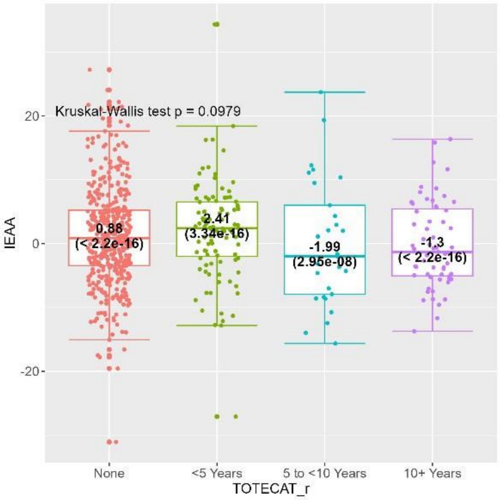

© 2025. Jung SY et al. Published online at http://www.aginganddisease.org/EN/10.14336/AD.2025.0099

Supplementary Data

Overall

KK. E plus P: DNAmAge, $p = 0.086$

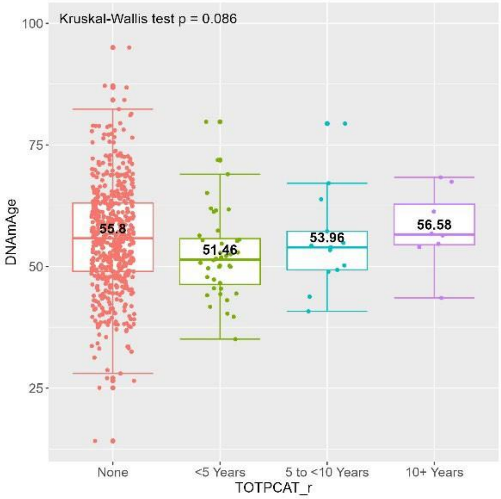

LL. E plus P: AgeAccelDiff, $p = 0.601$

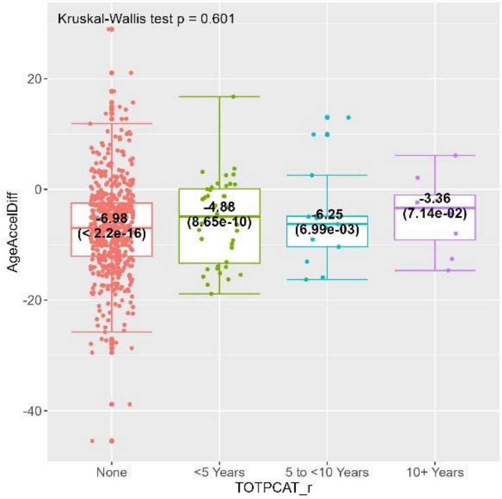

MM. E plus P: IEAA, $p = 0.917$

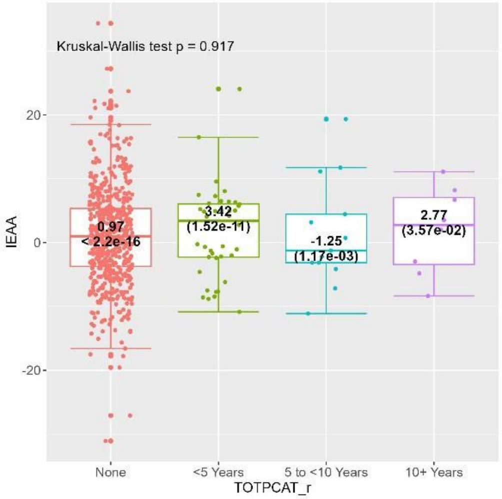

Supplementary Figure 3. Levine's clock: distribution of DNAmAge/AgeAccelDiff/IEAA by selected CRC risk factors. (AgeAccelDiff, epigenetic age acceleration as departure of DNAmAge from chronologic age; BMI, body mass index; CRC, colorectal cancer; DNAmAge, DNA methylation–based marker of aging; E only, exogenous estrogen only; E plus P, E plus progestin; HEI-2015, Health Eating Index-2015; IEAA, intrinsic epigenetic age acceleration as residuals adjusted for cell composition; WHR, waist-to-hip ratio.)

© 2025. Jung SY et al. Published online at http://www.aginganddisease.org/EN/10.14336/AD.2025.0099

Supplementary Data

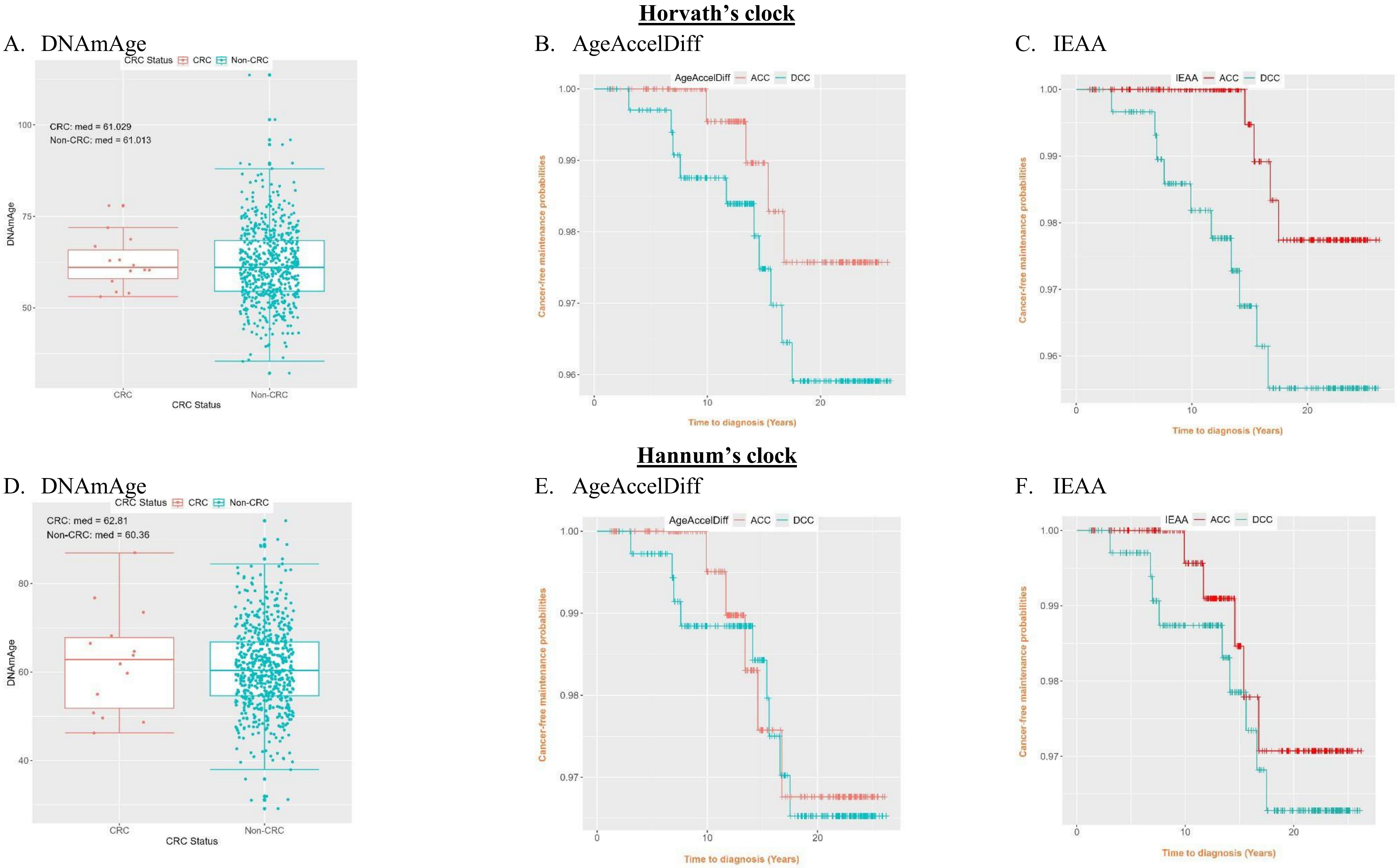

© 2025. Jung SY et al. Published online at http://www.aginganddisease.org/EN/10.14336/AD.2025.0099

Supplementary Data

Figure S4 (Continued)

Levine's clock

G. DNAmAge

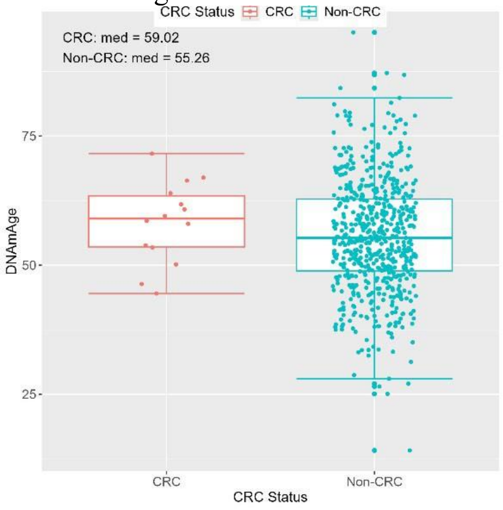

H. AgeAccelDiff

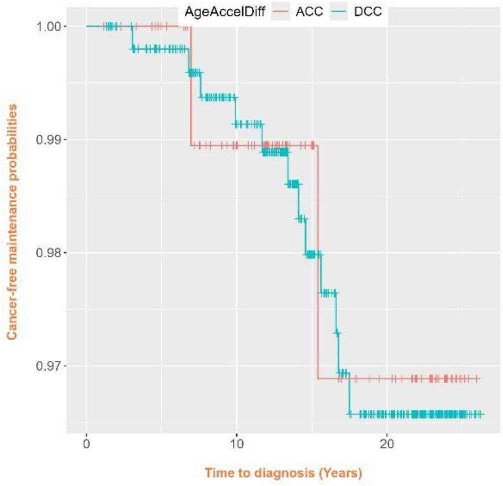

I. IEAA

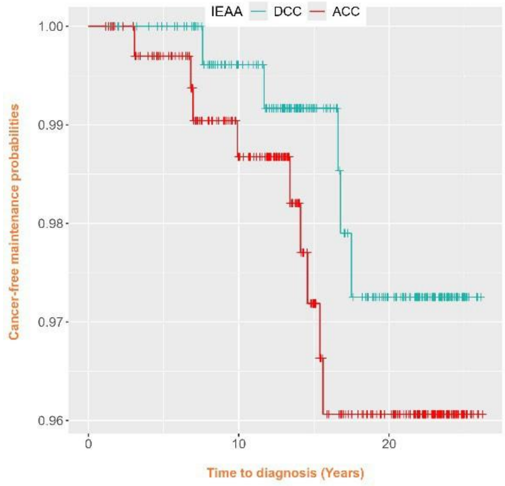

Supplementary Figure 4. Distribution of DNAmAge by CRC status and cancer-free probability curve of AgeAccelDiff/IEAA. (AgeAccelDiff, epigenetic age acceleration as departure of DNAmAge from chronologic age; ACC, acceleration, i.e., positive difference of DNAm age from chronologic age; CRC, colorectal cancer; DNAmAge, DNA methylation–based marker of aging; DCC, deceleration, i.e., negative difference of DNAm age from chronologic age; IEAA, intrinsic epigenetic age acceleration as residuals adjusted for cell composition.)

© 2025. Jung SY et al. Published online at http://www.aginganddisease.org/EN/10.14336/AD.2025.0099

Supplementary Data

< Correlation between DNAmAge/AgeAccelDiff/AgeAccelRes and chronologic age by CRC status >

<u>Overall</u>

A. DNAmAge

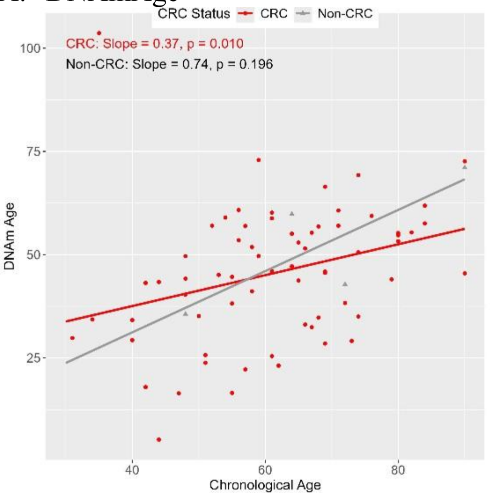

B. AgeAccelDiff

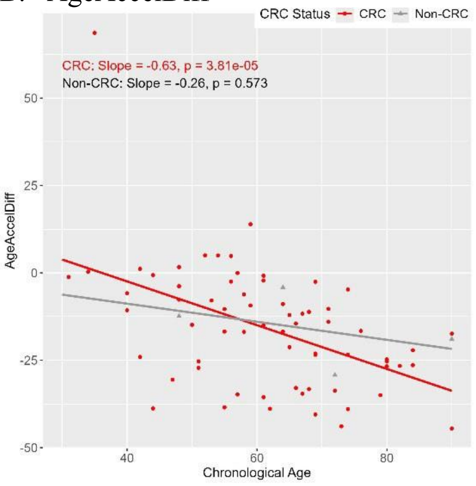

C. AgeAccelRes

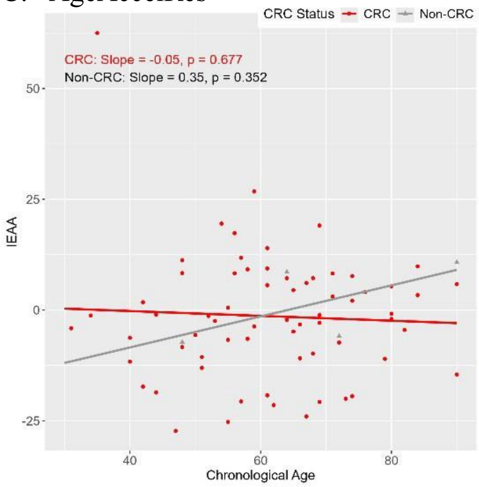

<u>Females only</u>

D. DNAmAge

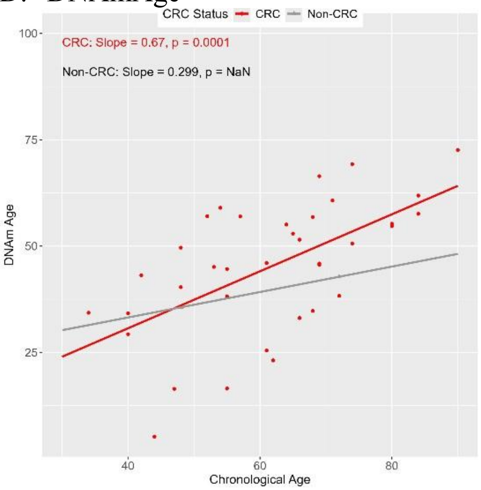

E. AgeAccelDiff

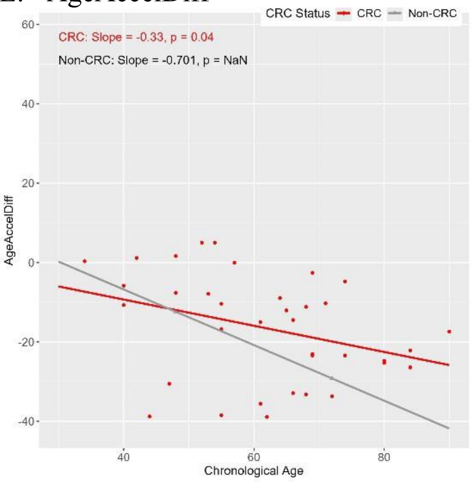

F. AgeAccelRes

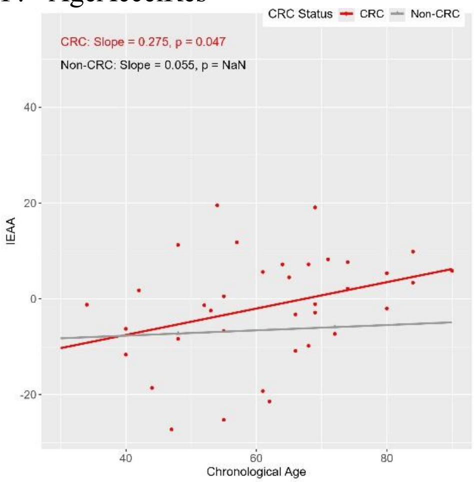

© 2025. Jung SY et al. Published online at http://www.aginganddisease.org/EN/10.14336/AD.2025.0099

Supplementary Data

< Distribution of DNAmAge/AgeAccelDiff/AgeAccelRes by CRC status >

<u>Overall</u>

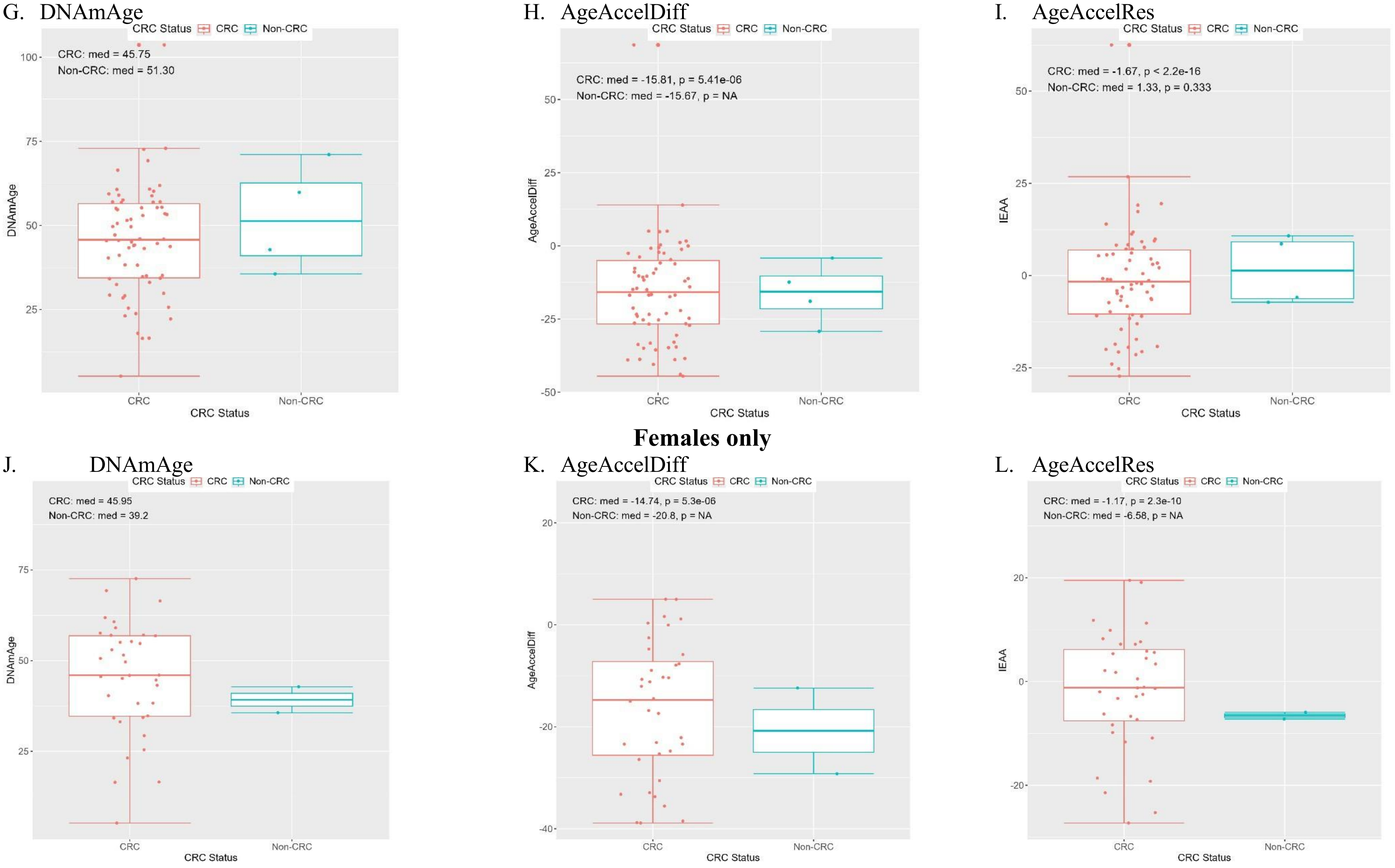

Supplementary Figure 5. TCGA, Horvath's clock: Validation tests. (AgeAccelDiff, epigenetic age acceleration as departure of DNAmAge from chronologic age; AgeAccelRes, epigenetic age acceleration as residuals by regressing DNAmAge on chronologic age; CRC, colorectal cancer; DNAmAge, DNA methylation–based marker of aging.)

© 2025. Jung SY et al. Published online at http://www.aginganddisease.org/EN/10.14336/AD.2025.0099

Supplementary Data

< Correlation between DNAmAge/AgeAccelDiff/AgeAccelRes and chronologic age by CRC status >

Overall

A. DNAmAge

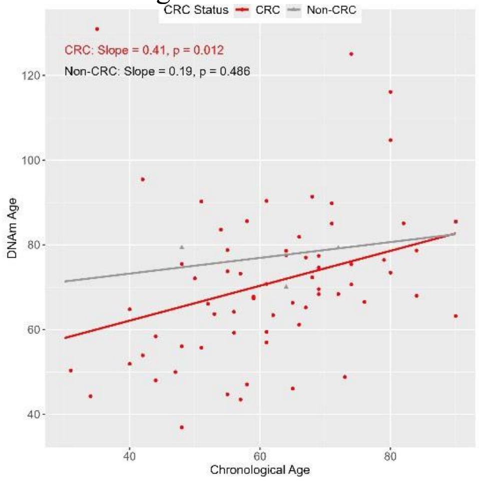

B. AgeAccelDiff

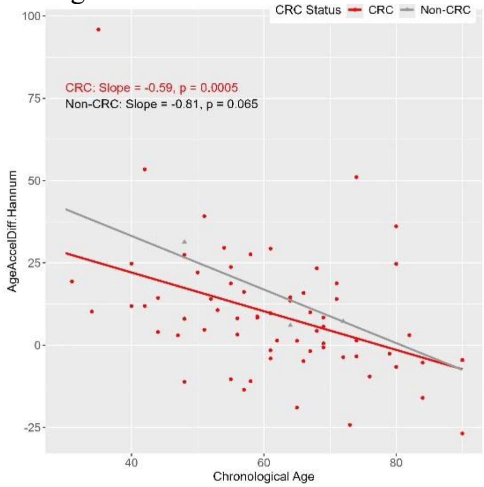

C. AgeAccelRes

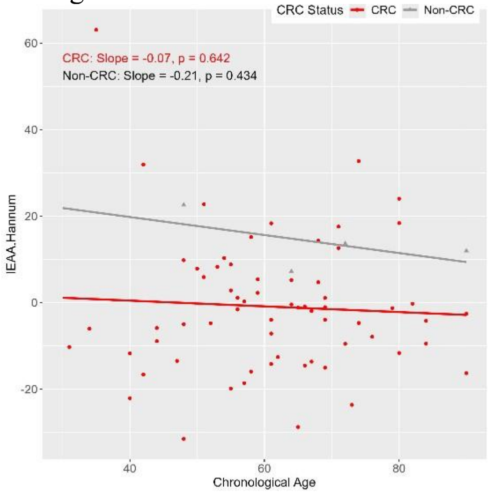

Females only

D. DNAmAge

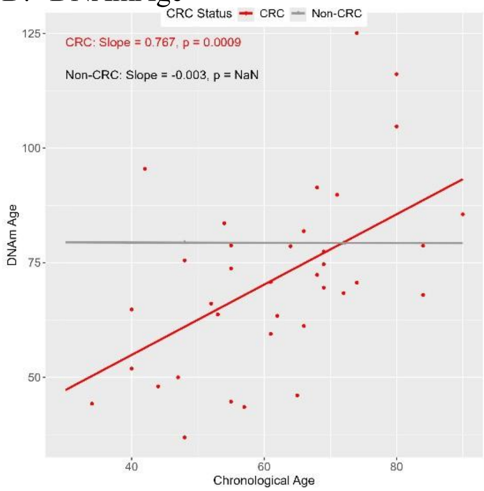

E. AgeAccelDiff

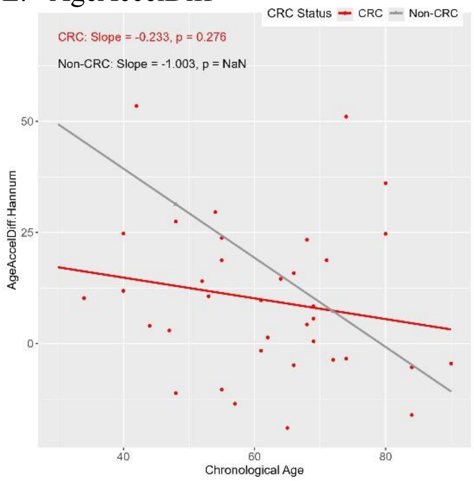

F. AgeAccelRes

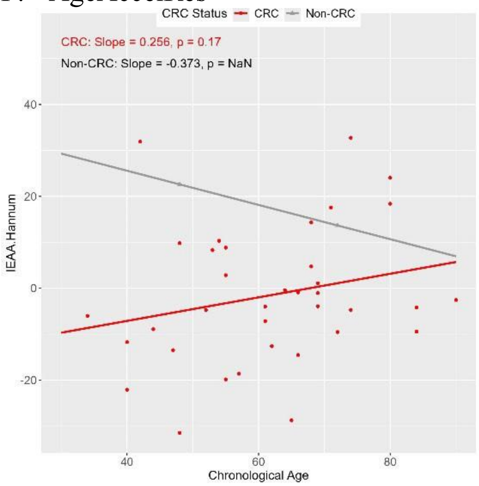

© 2025. Jung SY et al. Published online at http://www.aginganddisease.org/EN/10.14336/AD.2025.0099

Supplementary Data

< Distribution of DNAmAge/AgeAccelDiff/AgeAccelRes by CRC status >

<u>Overall</u>

G. DNAmAge H. AgeAccelDiff I. AgeAccelRes

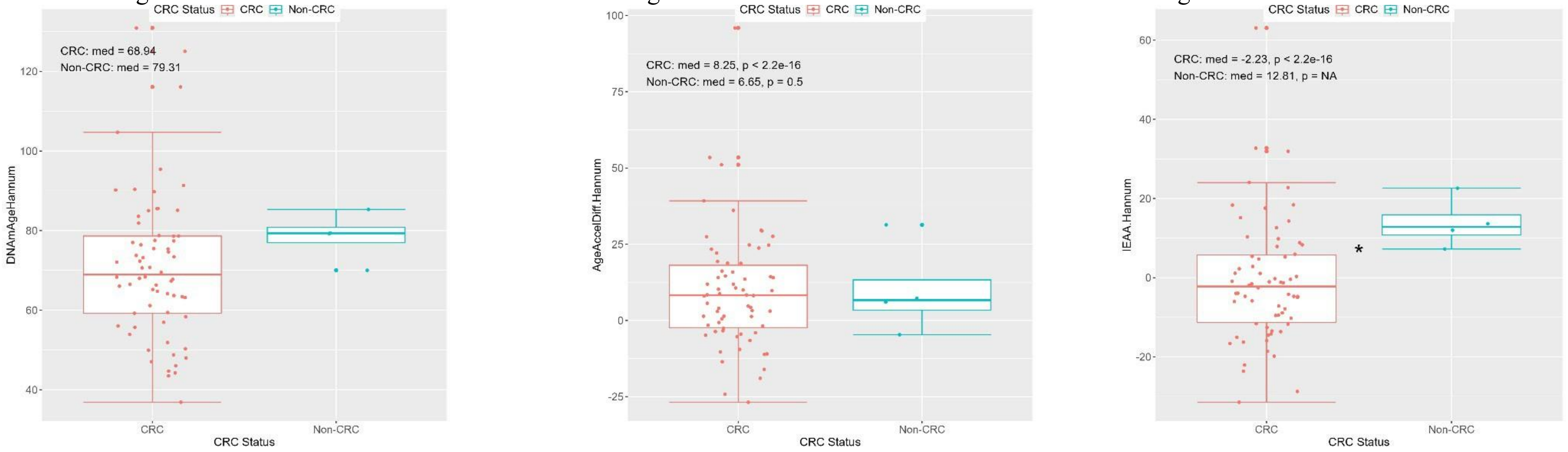

<u>Females only</u>

J. DNAmAge K. AgeAccelDiff L. AgeAccelRes

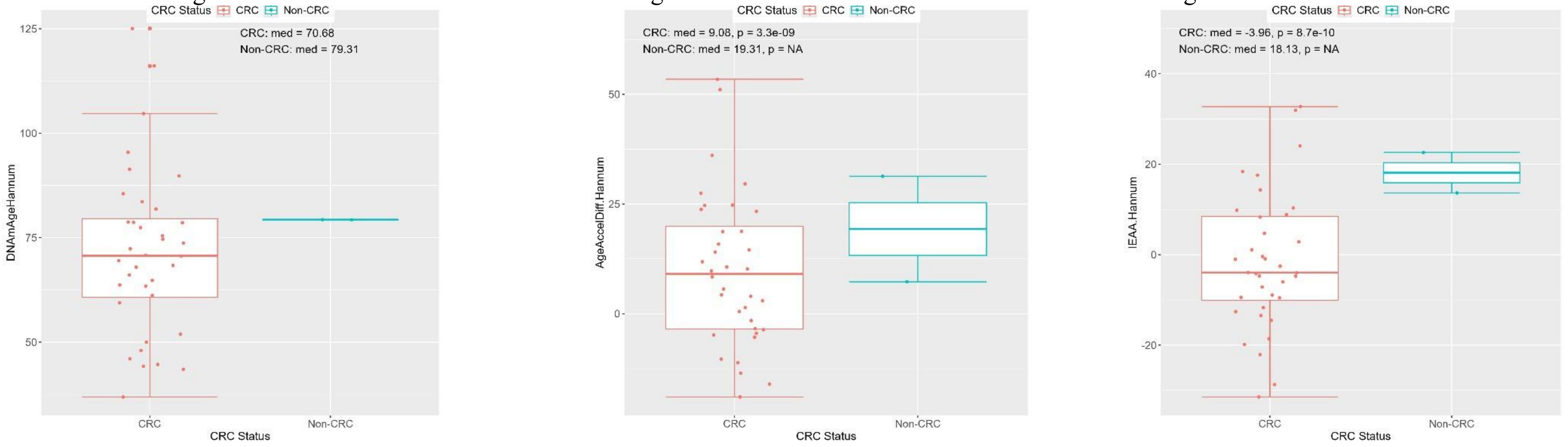

Supplementary Figure 6. TCGA, Hannum's clock: Validation tests. (AgeAccelDiff, epigenetic age acceleration as departure of DNAmAge from chronologic age; AgeAccelRes, epigenetic age acceleration as residuals by regressing DNAmAge on chronologic age; CRC, colorectal cancer; DNAmAge, DNA methylation–based marker of aging.)

© 2025. Jung SY et al. Published online at http://www.aginganddisease.org/EN/10.14336/AD.2025.0099

Supplementary Data

< Correlation between DNAmAge/AgeAccelDiff/AgeAccelRes and chronologic age by CRC status >

Overall

A. DNAmAge

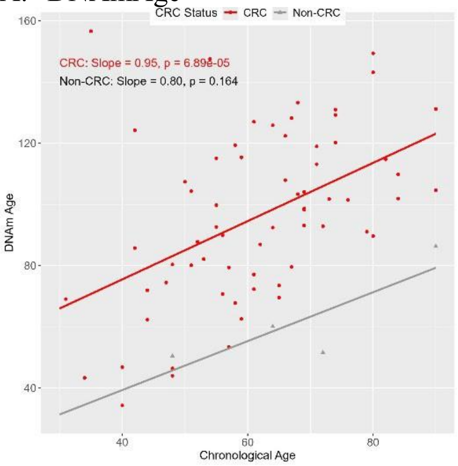

B. AgeAccelDiff

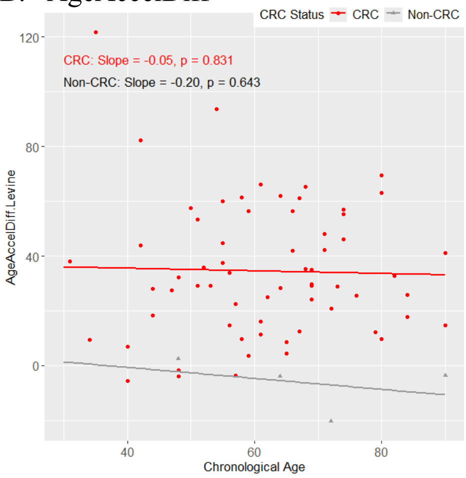

C. AgeAccelRes

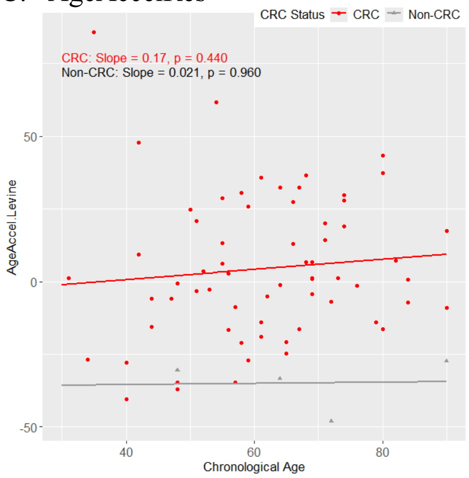

Females only

D. DNAmAge

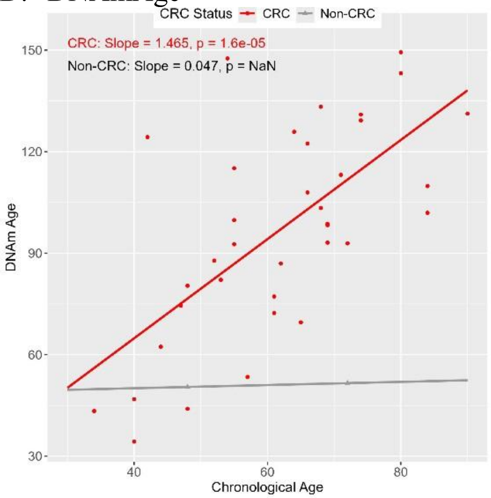

E. AgeAccelDiff

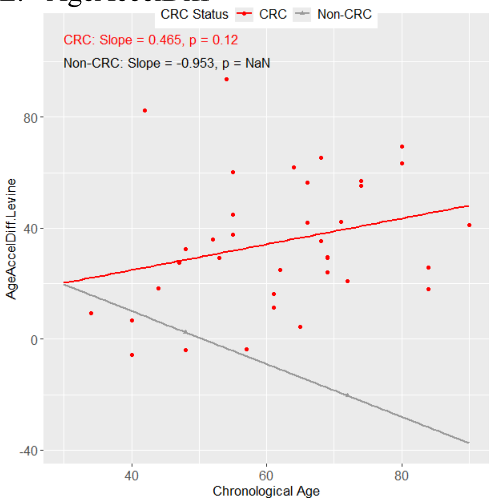

F. AgeAccelRes

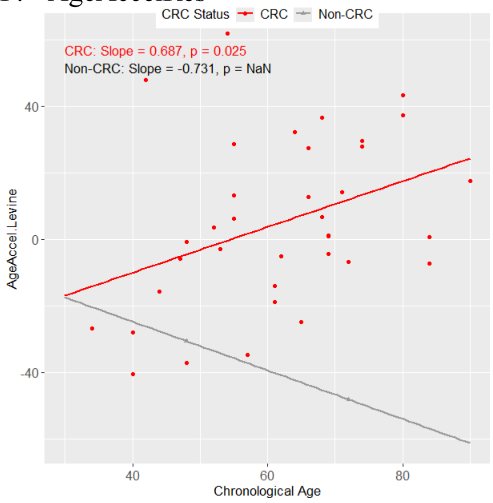

© 2025. Jung SY et al. Published online at http://www.aginganddisease.org/EN/10.14336/AD.2025.0099

Supplementary Data

< Distribution of DNAmAge/AgeAccelDiff/AgeAccelRes by CRC status >

Overall

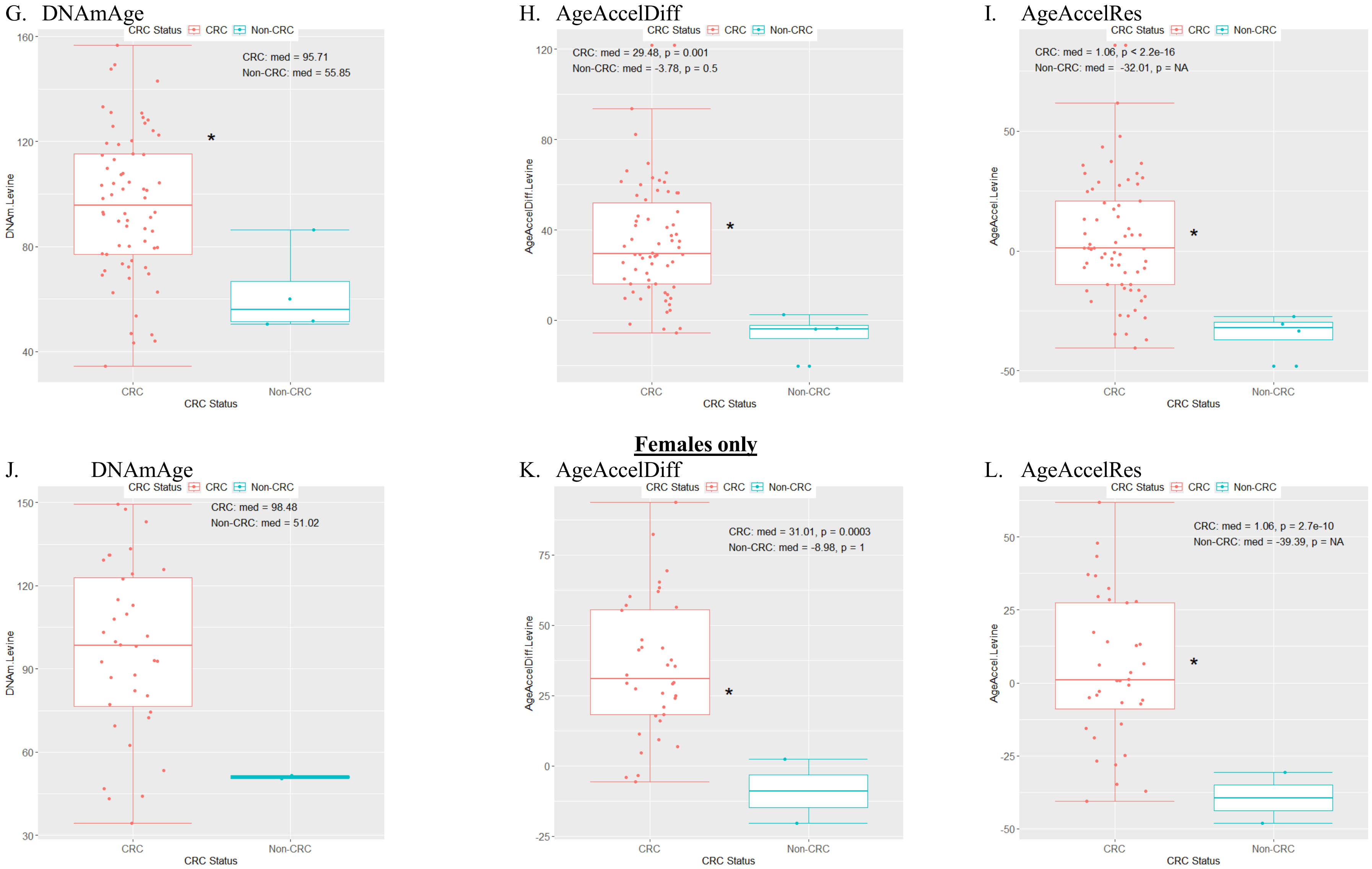

Supplementary Figure 7. TCGA, Levine's clock: Validation tests. (AgeAccelDiff, epigenetic age acceleration as departure of DNAmAge from chronologic age; AgeAccelRes, epigenetic age acceleration as residuals by regressing DNAmAge on chronologic age; CRC, colorectal cancer; DNAmAge, DNA methylation–based marker of aging.)

© 2025. Jung SY et al. Published online at http://www.aginganddisease.org/EN/10.14336/AD.2025.0099

Supplementary Data

< Correlation between DNAmAge/AgeAccelDiff/AgeAccelRes and chronologic age by CRC status >

Tumor tissues vs. adjacent normal tissues

A. DNAmAge

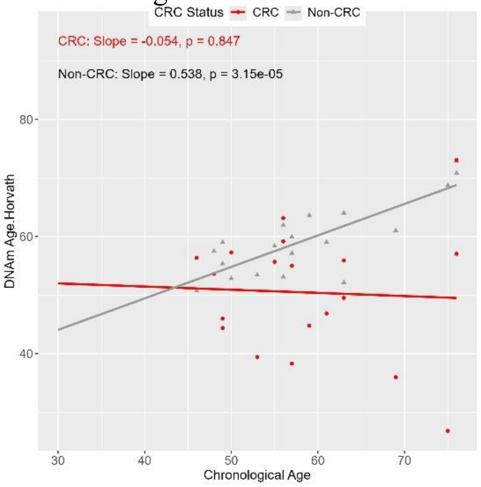

B. AgeAccelDiff

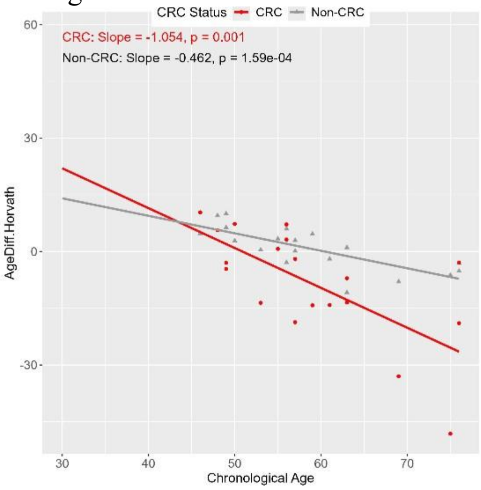

C. AgeAccelRes

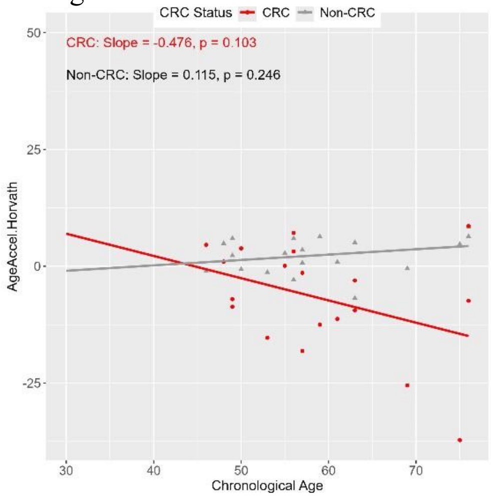

Tumor tissues vs. normal tissues from participants without cancer

D. DNAmAge

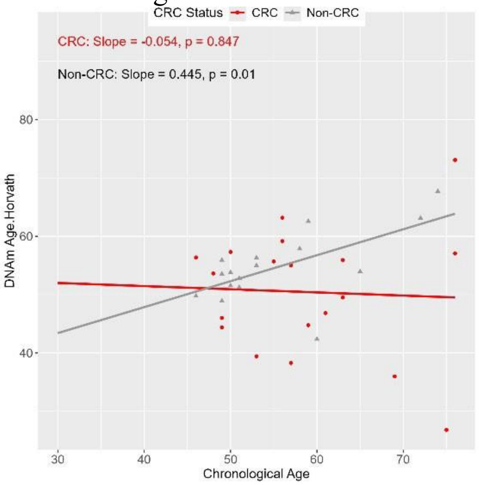

E. AgeAccelDiff

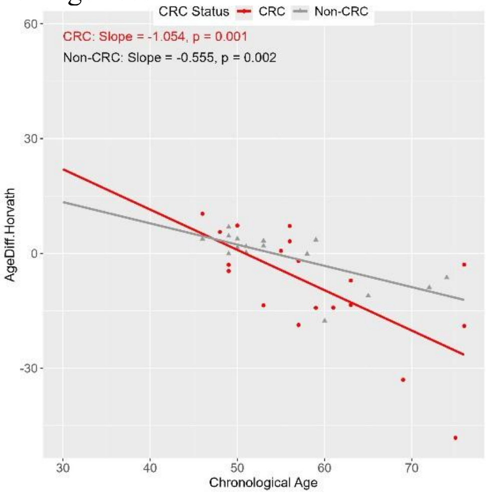

F. AgeAccelRes

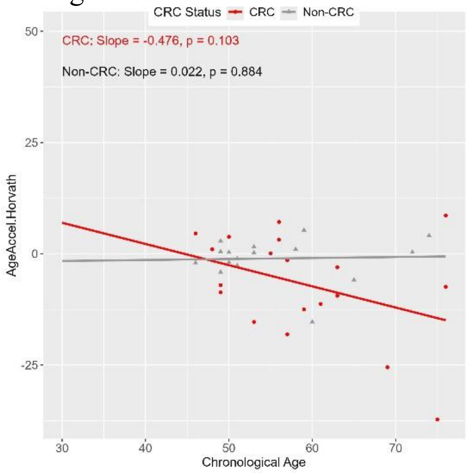

© 2025. Jung SY et al. Published online at http://www.aginganddisease.org/EN/10.14336/AD.2025.0099

Supplementary Data

< Distribution of DNAmAge/AgeAccelDiff/AgeAccelRes by CRC status >

Tumor tissues vs. adjacent normal tissues

G. DNAmAge H. AgeAccelDiff I. AgeAccelRes

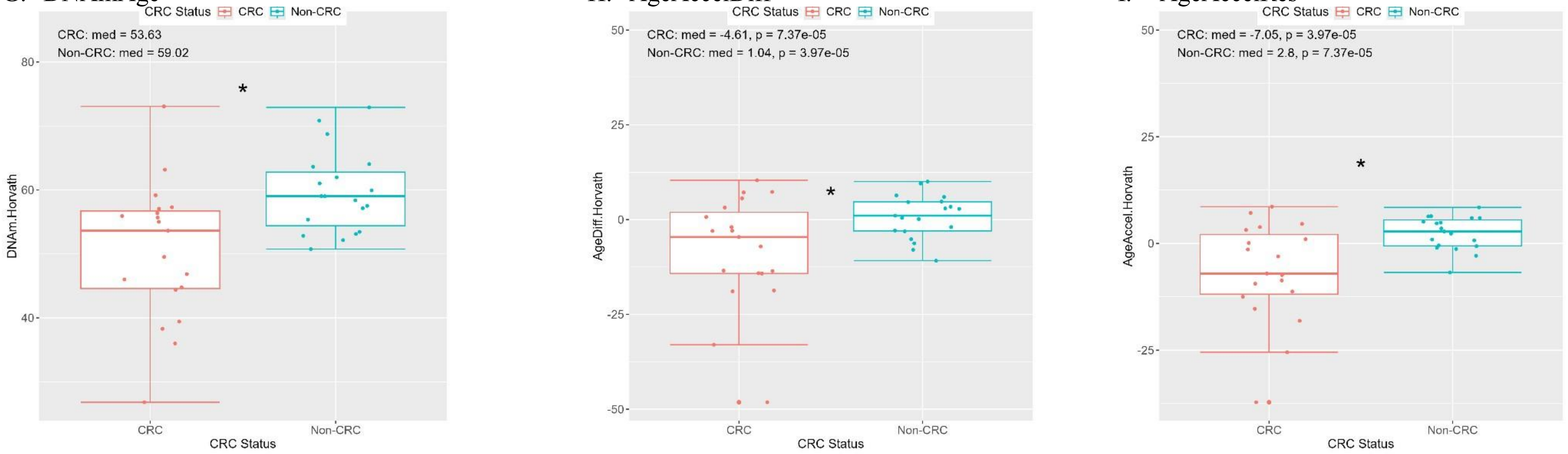

Tumor tissues vs. normal tissues from participants without cancer

J. DNAmAge K. AgeAccelDiff L. AgeAccelRes

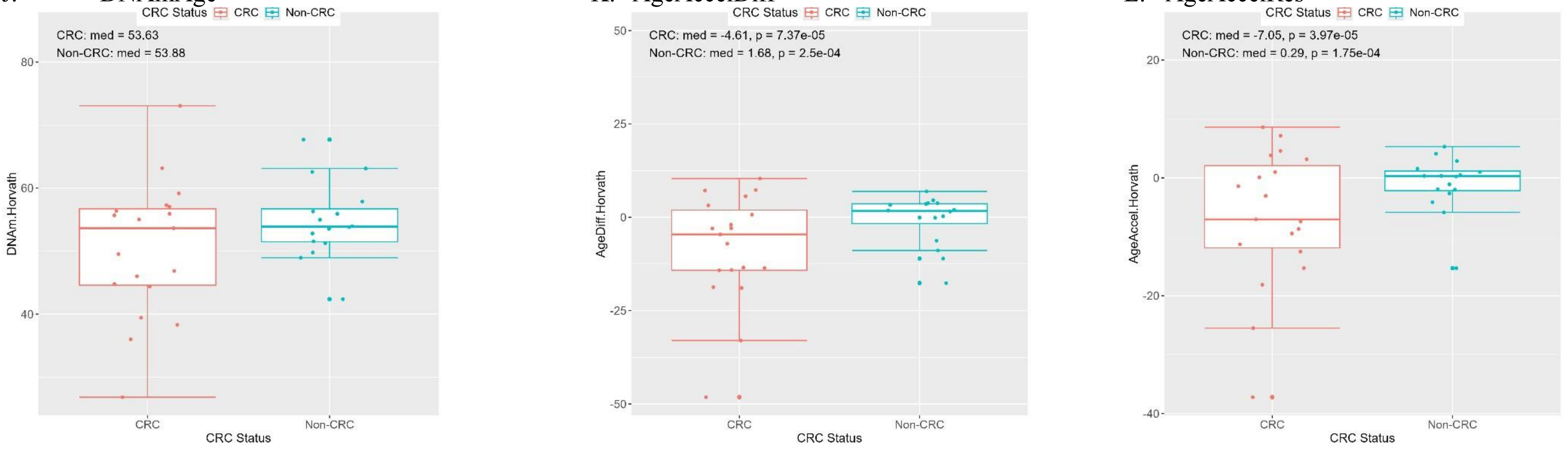

Supplementary Figure 8. GSE199057, Horvath's clock: Validation tests. Results from *females only.* (AgeAccelDiff, epigenetic age acceleration as departure of

© 2025. Jung SY et al. Published online at http://www.aginganddisease.org/EN/10.14336/AD.2025.0099

DNAmAge from chronologic age; AgeAccelRes, epigenetic age acceleration as residuals by regressing DNAmAge on chronologic age; CRC, colorectal cancer; DNAmAge, DNA methylation–based marker of aging.)

© 2025. Jung SY et al. Published online at http://www.aginganddisease.org/EN/10.14336/AD.2025.0099

Supplementary Data

< Correlation between DNAmAge/AgeAccelDiff/AgeAccelRes and chronologic age by CRC status >

<u>Tumor tissues vs. adjacent normal tissues</u>

A. DNAmAge

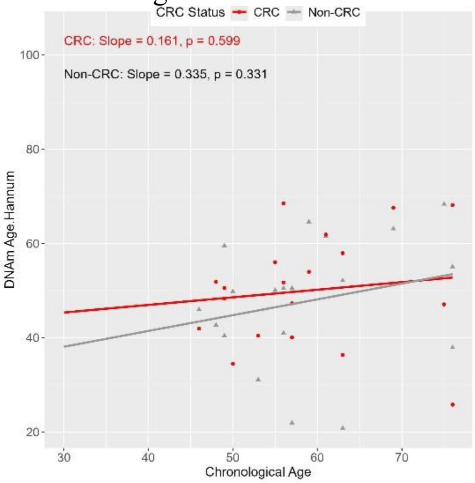

B. AgeAccelDiff

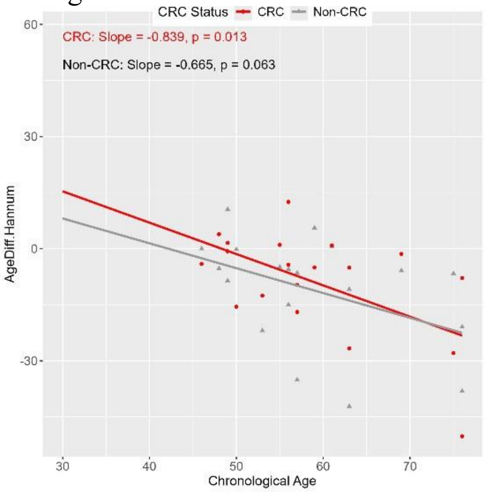

C. AgeAccelRes

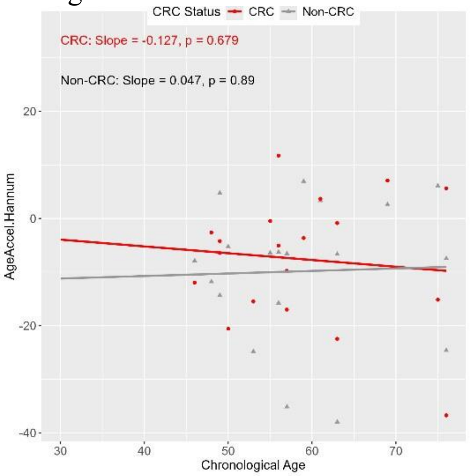

<u>Tumor tissues vs. normal tissues from participants without cancer</u>

D. DNAmAge

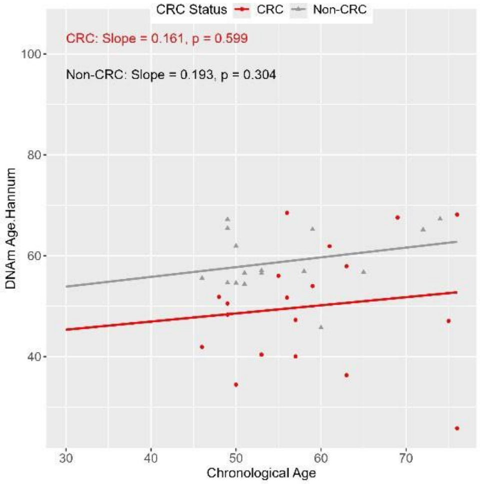

E. AgeAccelDiff

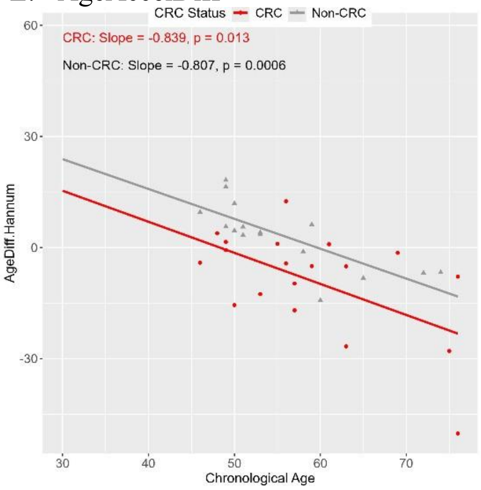

F. AgeAccelRes

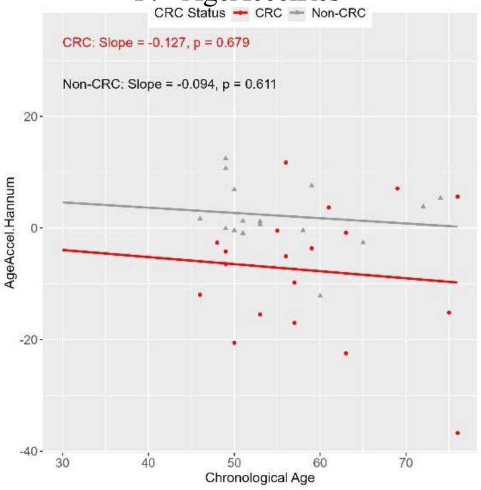

© 2025. Jung SY et al. Published online at http://www.aginganddisease.org/EN/10.14336/AD.2025.0099

Supplementary Data

< Distribution of DNAmAge/AgeAccelDiff/AgeAccelRes by CRC status >

Tumor tissues vs. adjacent normal tissues

G. DNAmAge

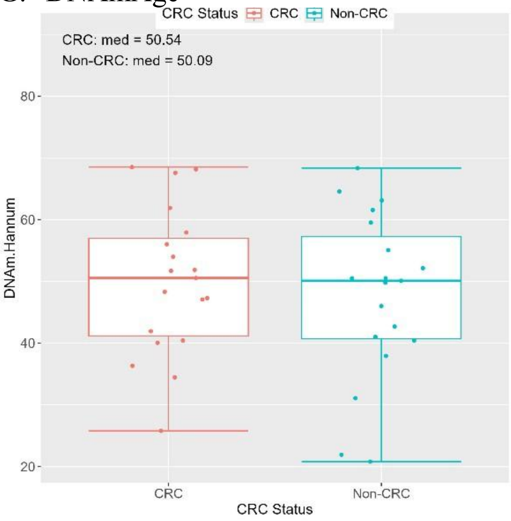

H. AgeAccelDiff

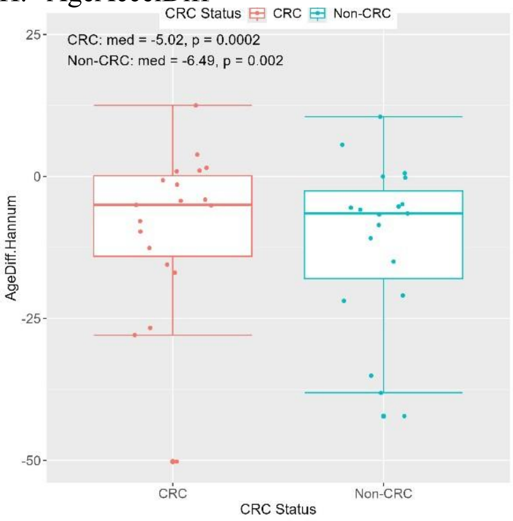

I. AgeAccelRes

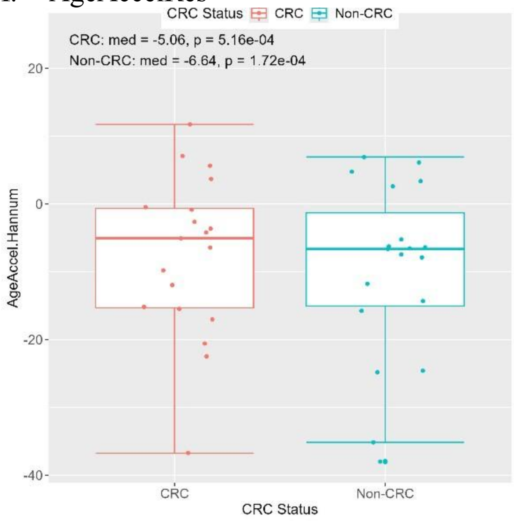

Tumor tissues vs. normal tissues from participants without cancer

J. DNAmAge

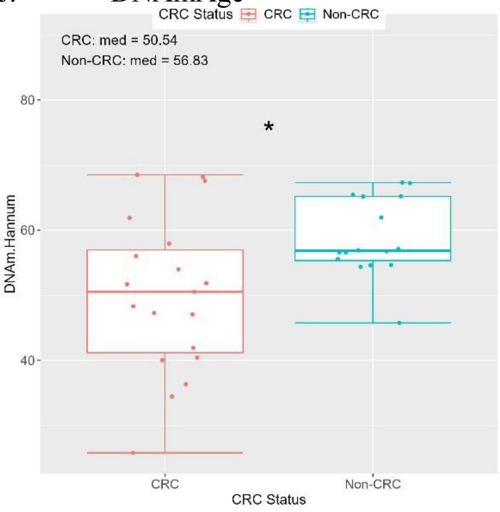

K. AgeAccelDiff

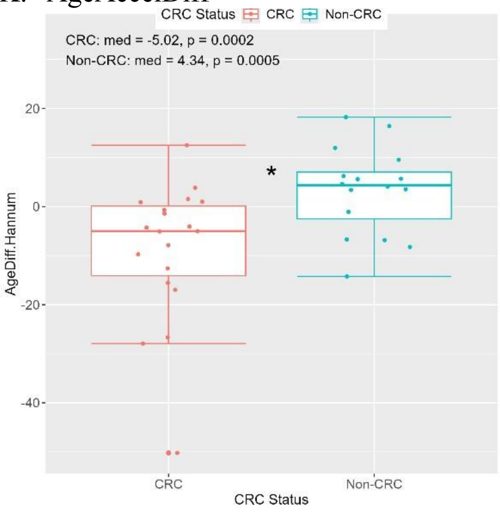

L. AgeAccelRes

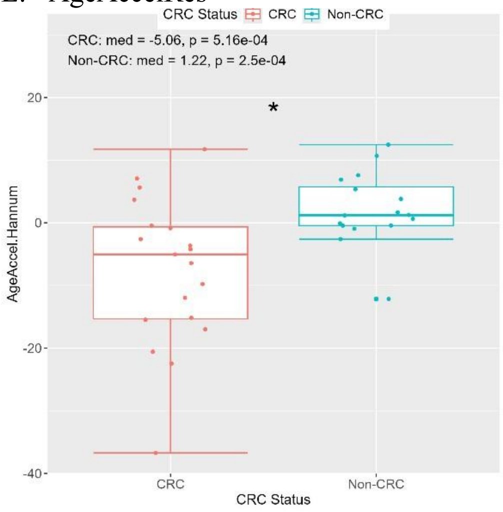

Supplementary Figure 9. GSE199057, Hannum's clock: Validation tests. Results from *females only*. (AgeAccelDiff, epigenetic age acceleration as departure of

© 2025. Jung SY et al. Published online at http://www.aginganddisease.org/EN/10.14336/AD.2025.0099

DNAmAge from chronologic age; AgeAccelRes, epigenetic age acceleration as residuals by regressing DNAmAge on chronologic age; CRC, colorectal cancer; DNAmAge, DNA methylation–based marker of aging.)

© 2025. Jung SY et al. Published online at http://www.aginganddisease.org/EN/10.14336/AD.2025.0099

Supplementary Data

< Correlation between DNAmAge/AgeAccelDiff/AgeAccelRes and chronologic age by CRC status >

Tumor tissues vs. adjacent normal tissues

A. DNAmAge

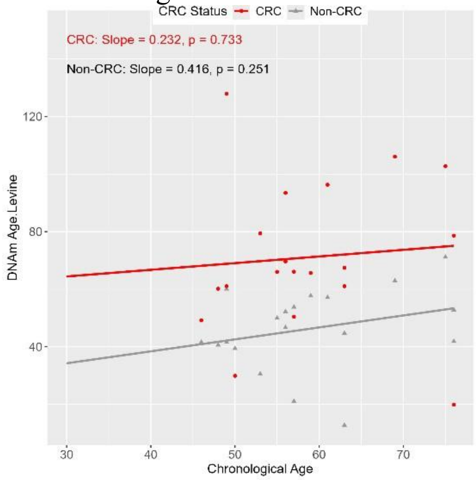

B. AgeAccelDiff

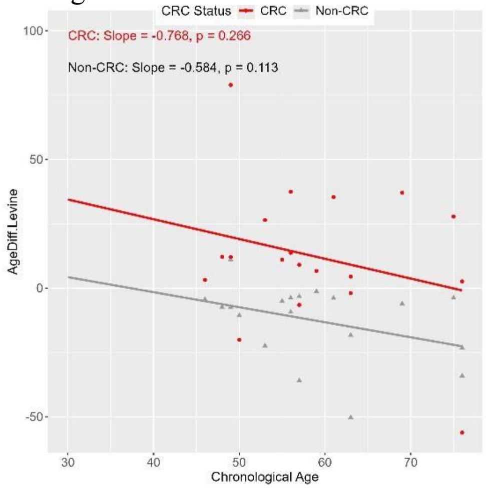

C. AgeAccelRes

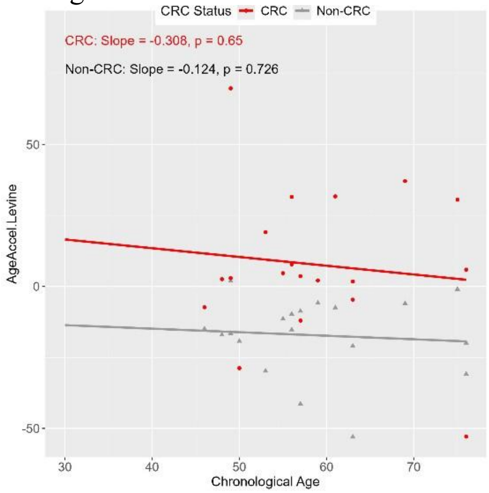

Tumor tissues vs. normal tissues from participants without cancer

D. DNAmAge

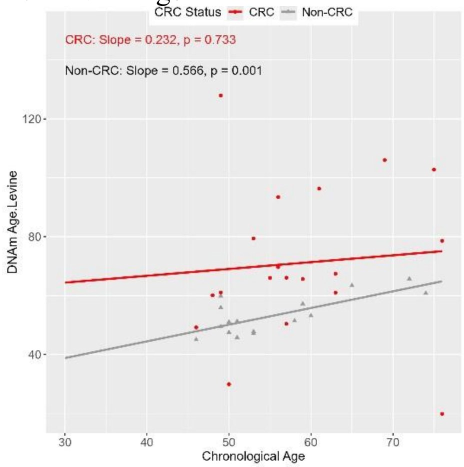

E. AgeAccelDiff

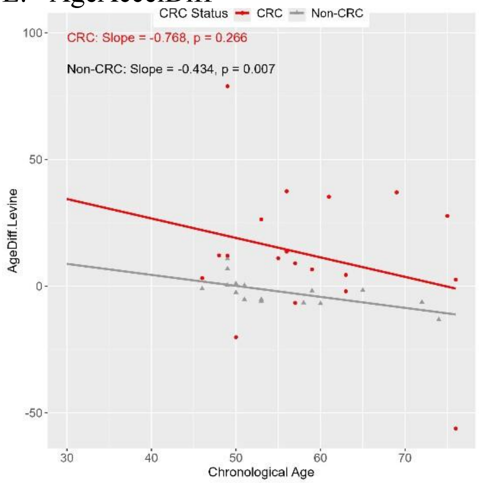

F. AgeAccelRes

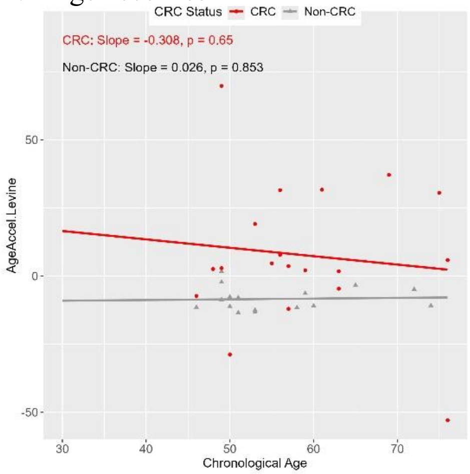

© 2025. Jung SY et al. Published online at http://www.aginganddisease.org/EN/10.14336/AD.2025.0099

SUPPLEMENTARY DATA

< Distribution of DNAmAge/AgeAccelDiff/AgeAccelRes by CRC status >

Tumor tissues vs. adjacent normal tissues

G. DNAmAge

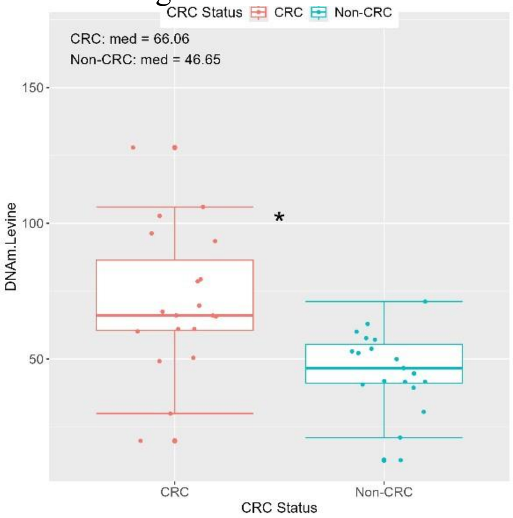

H. AgeAccelDiff

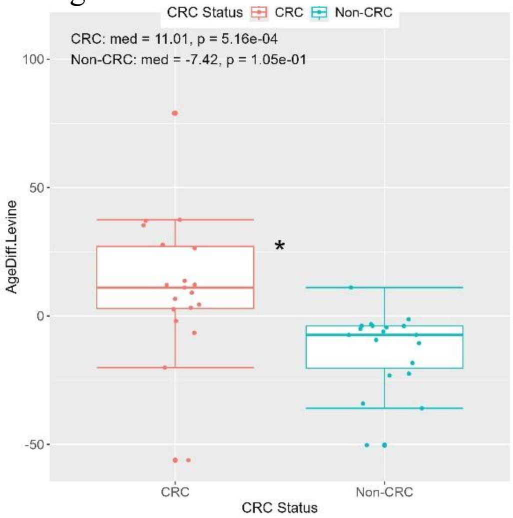

I. AgeAccelRes

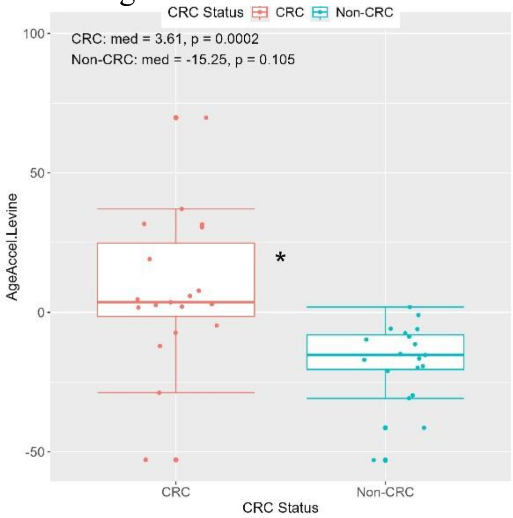

Tumor tissues vs. normal tissues from participants without cancer

J. DNAmAge

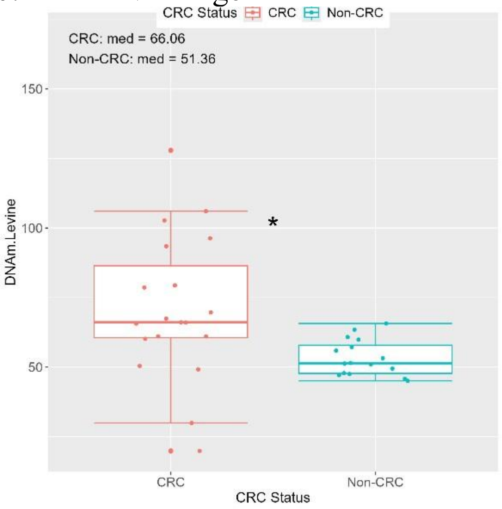

K. AgeAccelDiff

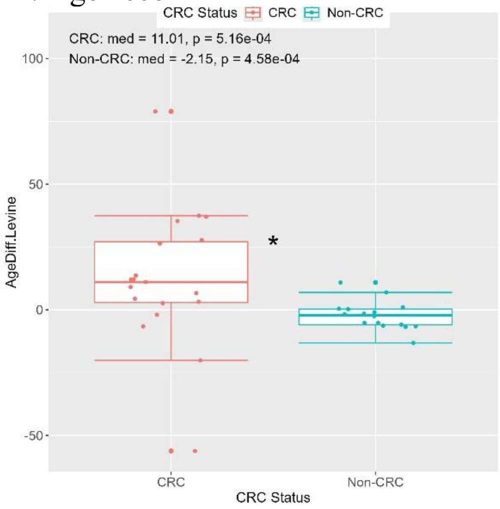

L. AgeAccelRes

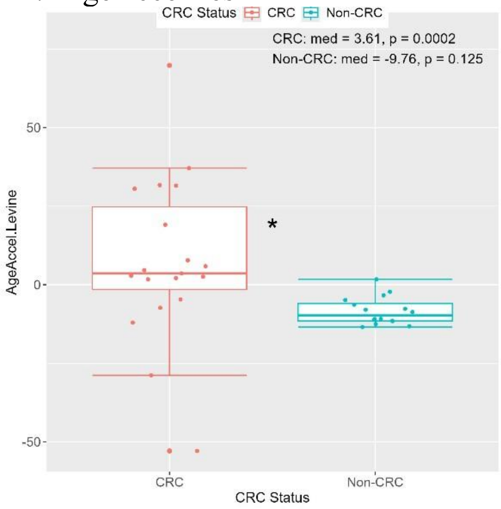

© 2025. Jung SY et al. Published online at http://www.aginganddisease.org/EN/10.14336/AD.2025.0099

SUPPLEMENTARY DATA

Supplementary Figure 10. GSE199057, Levine's clock: Validation tests. Results from *females only*. (AgeAccelDiff, epigenetic age acceleration as departure of DNAmAge from chronologic age; AgeAccelRes, epigenetic age acceleration as residuals by regressing DNAmAge on chronologic age; CRC, colorectal cancer; DNAmAge, DNA methylation–based marker of aging.)

© 2025. Jung SY et al. Published online at http://www.aginganddisease.org/EN/10.14336/AD.2025.0099
